# Gene expression profile in patients with Gaucher disease indicates activation of inflammatory processes

**DOI:** 10.1038/s41598-019-42584-1

**Published:** 2019-04-15

**Authors:** Agnieszka Ługowska, Katarzyna Hetmańczyk-Sawicka, Roksana Iwanicka-Nowicka, Anna Fogtman, Jarosław Cieśla, Joanna Karolina Purzycka-Olewiecka, Dominika Sitarska, Rafał Płoski, Mirella Filocamo, Susanna Lualdi, Małgorzata Bednarska-Makaruk, Marta Koblowska

**Affiliations:** 1 Department of Genetics, Institute of Psychiatry and Neurology, Warsaw, Poland; 2 Laboratory of Microarray Analysis, Institute of Biochemistry and Biophysics, Polish Academy of Sciences, Warsaw, Poland; 3 Laboratory of Systems Biology, Faculty of Biology, University of Warsaw, Warsaw, Poland; 4 Institute of Biochemistry and Biophysics, Polish Academy of Sciences, Warsaw, Poland; 5 Department of Medical Genetics, Warsaw Medical University, Warsaw, Poland; 6 Laboratorio di Genetica Molecolare e Biobanche, Istituto G. Gaslini, L.go G. Gaslini -16147, Genova, Italy

## Abstract

Gaucher disease (GD) is a rare inherited metabolic disease caused by pathogenic variants in the *GBA1* gene. So far, the pathomechanism of GD was investigated mainly in animal models. In order to delineate the molecular changes in GD cells we analysed gene expression profile in cultured skin fibroblasts from GD patients, control individuals and, additionally, patients with Niemann-Pick type C disease (NPC). We used expression microarrays with subsequent validation by qRT-PCR method. In the comparison GD patients vs. controls, the most pronounced relative fold change (rFC) in expression was observed for genes *IL13RA2* and *IFI6* (up-regulated) and *ATOH8* and *CRISPLD2* (down-regulated). Products of up-regulated and down-regulated genes were both enriched in genes associated with immune response. In addition, products of down-regulated genes were associated with cell-to-cell and cell-to-matrix interactions, matrix remodelling, PI3K-Akt signalling pathway and a neuronal survival pathway. Up-regulation of *PLAU*, *IFIT1*, *TMEM158* and down-regulation of *ATOH8* and *ISLR* distinguished GD patients from both NPC patients and healthy controls. Our results emphasize the inflammatory character of changes occurring in human GD cells indicating that further studies on novel therapeutics for GD should consider anti-inflammatory agents.

## Introduction

Gaucher disease (GD) is one of rare inherited metabolic disorders with the incidence ranging from 1:40 000 to 1: 60 000 among live births in the general population and about 1:800 among the Ashkenazi Jews^1,2^.

In majority of cases GD is caused by the deficient activity of a lysosomal hydrolase – acid beta-glucosidase, also called glucocerebrosidase [EC 3.2.1.45], due to pathogenic variants in the glucocerebrosidase gene (*GBA1*; MIM #606463; GenBank accession #J03059.1). During the disease course non-degraded substrates accumulate in macrophages of liver, spleen, bone marrow (characteristic Gaucher cells in the bone marrow), and other organs. The most abundant stored material is glucocerebroside (glucosylceramide; GlcCer). Other compounds which are accumulated to a lesser extent include glucosylsphingosine (lyso-Gl-1) which has been recently used as a primary diagnostic and follow-up monitoring biomarker in GD^3,4^. Very rarely GD results from the lack of functionally active saposin C (SapC) protein encoded by part of prosaposin gene (*PSAP*). Saposin C acts as a cofactor of glucocerebrosidase and patients affected with GD caused by its deficiency display accumulation of GlcCer and elevation of chitotriosidase activity but normal glucocerebrosidase activity. Both neuronopathic and non-neuronopathic types of GD caused by SapC deficiency have been described^5–7^.

Conventionally, three main clinical phenotypes of GD are distinguished depending on the age of onset and clinical severity: type 1 – non-neuronopatic (MIM# 230800), type 2 – neuronopathic severe (MIM# 230900), and type 3 – neuronopathic with milder course (MIM# 231000). However, it has been observed that a continuum of clinical phenotypes exists between types 2 and 3 of GD and although type 1 GD is potentially considered as non-neuronopathic there are individuals who develop neurological symptoms such as Parkinsonism, seizures, oligophrenia or sensorineural hearing loss^8–10^.

Although the identity of material stored in activated macrophages is known, the pathomechanism linking its accumulation with the clinical manifestations remains unexplained. Recently, importance is attached to intoxication with lysolipids, especially with glucosylsphingosine (lyso-Gl-1 or lyso-glucosylceramide) which leads to activation of inflammatory processes^11–13^. Lyso-Gl-1 influences neurons, lymphocytes and macrophages by down-regulation of *Bcl2* and up-regulation of *Bax* genes, and its derivative – sphingosine - influences bones^14,15^.

Since GD is a rare disease and collection of samples takes many years we decided to use in our experiments the lines of skin fibroblasts frozen in liquid nitrogen. Fibroblasts are not considered to have significant GlcCer or other substrates accumulation as compared to macrophages but the lipid storage is still measurable and elevated. In particular, Fuller *et al*. showed that in cultured GD skin fibroblasts there was an accumulation of GlcCer as well as ceramide, dihexosylceramide, trihexosylceramide, sphingomyelin, phosphatidylcholine, phosphatidylglycerol, and phosphatidylinositol which all most probably affected cellular pathways^16^.

During our experiments it was not possible to obtain blood or other samples from living GD patients not treated with enzyme replacement therapy. Thus, we were unable to compare gene expression profiles in other cells than cultured skin fibroblasts and to check the usefulness of new potential biomarkers. In order to compare GD with other lysosomal storage diseases (LSD), we additionally studied samples from patients with Niemann-Pick disease type C (NPC). The rationale for this was the generally observed overlap in clinical symptoms between the two diseases at their onset.

So far, only Moran *et al*.^17^ performed global gene expression study on tissues from GD patients. They showed increased activities of cathepsins B, K, and S in 4 GD spleens suggesting that enhanced expression of cysteine proteinases promotes tissue destruction. In particular, it was proposed that cathepsin K was involved in the breakdown of the extracellular matrix^17^. Additionally, Moran *et al*.^17^ found elevated levels of transcripts encoding the pulmonary and activation-regulated chemokine (PARC) and gpNMB protein.

The aim of our study was to further delineate molecular pathology in human GD cells by the analysis of gene expression profile using genomewide technologies. At the first step we performed microarray gene expression studies whose results were subsequently confirmed by quantitative real-time PCR (qRT-PCR) experiments. We have found that products of up-regulated genes were engaged in immune response, while products of down-regulated genes were involved also in immune response, in cell-to-cell and cell-to-matrix interactions, matrix remodelling, PI3K-Akt signalling pathway, and displayed a function in a neuronal survival pathway.

Another aim was to identify biomarker (s) potentially useful for diagnosis of GD, for monitoring the effects of enzyme replacement or substrate deprivation therapies and/-or for distinguishing patients affected with GD and NPC at an early disease stage. Currently existing common diagnostic biomarkers such as chitotriosidase, CCL-18, lyso-Gl1, lyso-SM, and lyso-SM-509 are not informative in every patient and have limitations (see the Discussion).

## Results

### Gene expression

Both, microarray studies and qRT-PCR analyses were performed on RNA samples isolated from cultured skin fibroblasts taken of 5 healthy individuals, 5 patients with GD, and 5 patients with NPC due to pathogenic mutations in the *NPC1* gene. Each cDNA sample used in qRT-PCR experiments was analysed in 3 solutions (biological repeats) and each solution was analysed in duplicates (technical repeats), for more details, please see the ‘Methods’ part.

To identify gene expression profiles characteristic of GD individuals vs. controls, the expression pattern of more than 25 000 genes with more than 47 000 probes (Illumina Inc., CA, USA) were analysed on the HumanHT-12 v4.0 Expression BeadChip arrays. We found up-regulation of 172 genes and down-regulation of 83 genes in GD patients vs. controls (see Supplementary Table S1 in Supplementary Information). Comparison of the expression profile of GD with NPC patients showed 256 genes with increased expression and 68 genes with decreased expression (Supplementary Table S2).

#### Comparison - GD patients vs. controls

**Figure 1:**
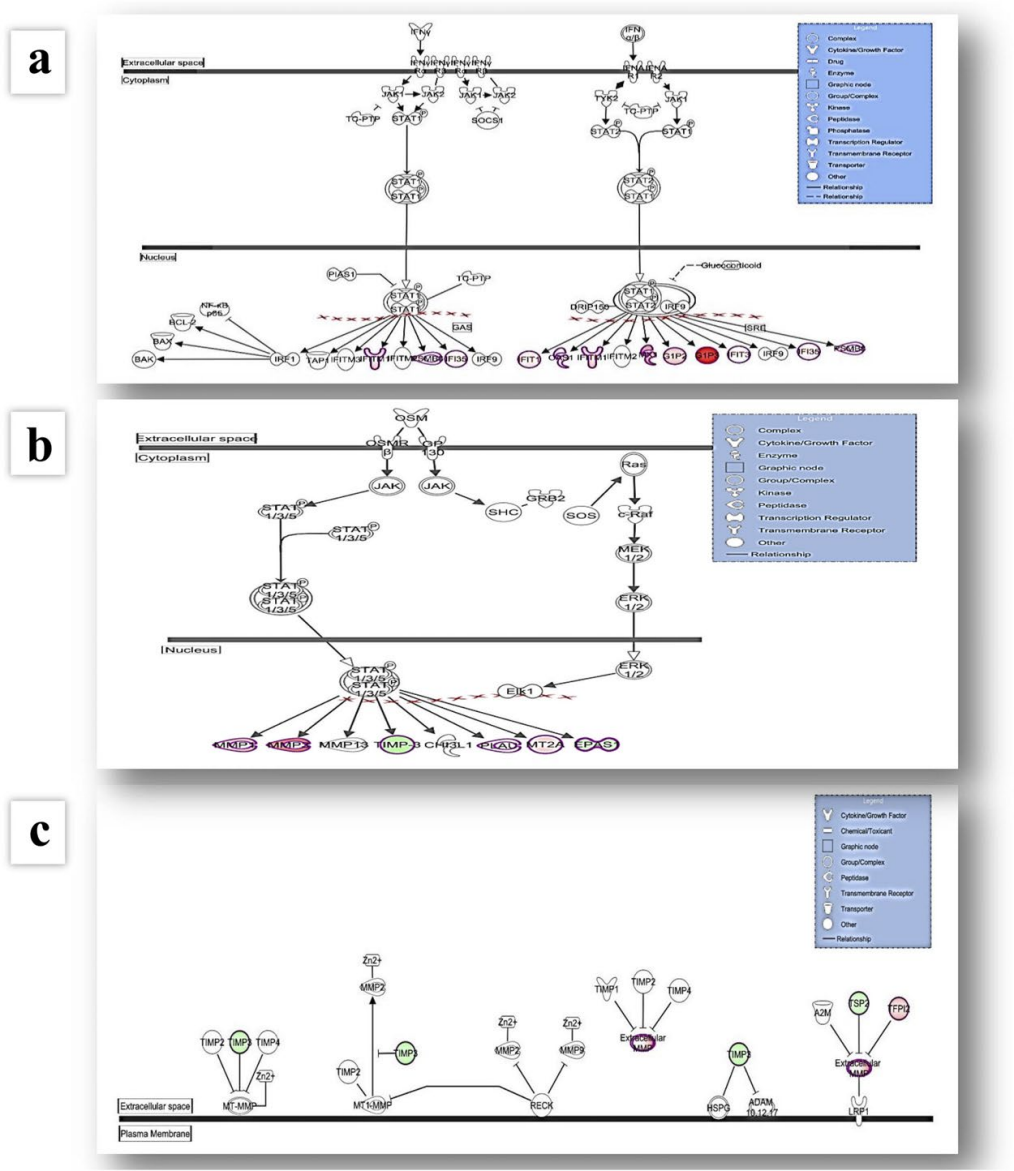
Graphical representation of the Interferon Signalling (**a**), Oncostatin M Signalling (**b**) and Inhibition of Matrix Metalloproteases (**c**) pathways. The networks were generated through the use of IPA (QIAGEN Inc., https://www.qiagenbio-informatics.com/products/ingenuity-pathway-analysis). Figures represent cellular canonical pathways with most differentially expressed genes after analyses of results obtained for samples of Gaucher patients vs. controls and Gaucher patients vs. Niemann-Pick type C patients. Genes with up-regulated expression are represented in red, while genes with down-regulated expression in green.

Ingenuity Pathway Analysis (IPA) revealed that genes differentially expressed in GD were associated with the following cellular canonical pathways: Interferon Signalling, Activation of IRF (interferon regulatory transcription factor) by Cytosolic Pattern Recognition Receptors, Protein Ubiquitination Pathway, Oncostatin M Signalling, Coagulation System, Hepatic Fibrosis/Hepatic Stellate Cell Activation, Adipogenesis pathway, Parkinson’s Signalling, Glioma Invasiveness Signalling, VDR/RXR Activation, Inhibition of Matrix Metalloproteases, LXR/RXR Activation, Glucocorticoid Receptor Signalling, see Table 1, Fig. 1, Supplementary Table S3.

**Table 1:** Results of microarray studies after IPA analysis.

Ingenuity (IPA) Canonical Pathways	Molecules
**(A) GD patients vs. controls**	
Interferon Signalling	IFIT3, IFIT1**, OAS1, **IFI6**, IRF9, STAT1, TAP1, **ISG15**
Activation of IRF by Cytosolic Pattern Recognition Receptors	IFIH1, IRF9, STAT1, **IFIT2**, **ISG15**
Protein Ubiquitination Pathway	**UCHL1**, B2M, PSME1, DNAJB4, TAP1, UBE2L6, UCHL3
Oncostatin M Signalling	**PLAU**, STAT1
Coagulation System	**PLAU**, TFPI
Hepatic Fibrosis/Hepatic Stellate Cell Activation	LY96, COL4A5, **IGFBP5**, STAT1
Adipogenesis pathway	LPIN1, SMAD9, **TXNIP**
Parkinson’s Signalling	**UCHL1**
Glioma Invasiveness Signalling	RND3, **PLAU**
VDR/RXR Activation	**IGFBP5**, KLF4
Inhibition of Matrix Metalloproteases	**THBS2**
LXR/RXR Activation	LY96, **MYLIP**
Glucocorticoid Receptor Signalling	BCL2L1, **PLAU**, STAT1
**(B) GD patients vs. NPC patients**	
Interferon Signalling	IFIT3,**IFIT1**,OAS1,**MX1**,STAT1,TAP1,**ISG15**
Protein Ubiquitination Pathway	PSMA6,DNAJB4,HSPA9,PSME2,TAP1,UCHL3, **UCHL1**,USO1, HSP90B1,HLA-C,PSMA4,HSPB7, PSMD14,PSMC2,UBE2E1
Activation of IRF by Cytosolic Pattern Recognition Receptors	STAT1,NFKB1,IFIT2,**ISG15**
Glioma Invasiveness Signalling	RND3,RHOB,MMP2,**PLAU**
Oncostatin M Signalling	**PLAU**,STAT1
Coagulation System	PROS1,**PLAU**
IL-17A Signalling in Fibroblasts	FOS***, NFKB1
Prostanoid Biosynthesis	**PTGIS**
Glucocorticoid Receptor Signalling	FOS,HSP90B1,HSPA9,POLR2H,**PLAU**,STAT1,NFKB1
Parkinson’s Signalling	**UCHL1**

Cellular canonical pathways with most differentially expressed genes. Comparison ‘GD patients vs. controls’ (part A) and comparison ‘GD patients vs. NPC patients’ (part B)*. ^*^Data were analysed through the use of IPA (QIAGEN Inc., https://www.qiagenbioinformatics.com/products/ingenuity-pathway-analysis).

**Bolded and underlined are genes with most enhanced or inhibited expression after microarray study. *** and 64 other pathways in which FOS was involved, see Supplementary Table S4 part A (all canonical pathways with changed expression).

In Table 2 we show genes differentially expressed in GD patients in comparison to controls and NPC patients.

**Table 2:** Gene profile characteristic of GD patients in comparison to controls and NPC patients.

Comparison			
GD patients vs.cControls		GD patients vs. NPC patients	
Genes with Changed Expression			
UP-regulated	Fold change	UP-regulated	Fold change
*SERPINB2*	3,675	MX1*	3,139
***PLAU***	**3,242**	***TMEM158***	**2,786**
IL13RA2*	2,879	*UCHL1*	2,727
IFI6*	2,769	*ISG15*	2,637
*TXNIP*	2,605	PLAU*	**2,591**
*IFIT2*	2,568	*FOS*	2,508
***IFIT1***	**2,555**	*MLLT11*	2,473
*NPTX1*	2,473	***IFIT1***	**2,384**
***TMEM158***	**2,457**	*PLOD2*	2,380
*WLS*	2,362	*IGF2BP3*	2,256
**DOWN-regulated**	**Fold change**	**DOWN-regulated**	**Fold change**
*IGFBP5*	−4,027	*PTGIS*	−3,661
CRISPLD2*	−2,689	ATOH8*	**−3,150**
*THBS2*	−2,445	MXRA5*	−2,926
ATOH8*	**−2,354**	MN1*	−2,837
*NNMT*	−2,323	*FOXQ1*	−2,743
*CNN1*	−2,103	*CRIP1*	−2,648
***ISLR***	**−2,070**	*COMP*	−2,573
*C1QTNF5*	−2,023	*HSPB7*	−2,478
*CSRP1*	−1,998	*TSHZ2*	−2,414
*MYLIP*	−1,988	***ISLR***	**−2,250**

Results of microarray studies were analysed with IPA program. Genes with changed expression, characteristic of GD patients are bolded and underlined. Genes with asterisks displayed the most remarkable enhancement or inhibition after qRT-PCR analyses.

The results of microarray studies were validated by the quantitative real time PCR (qRT-PCR). We have chosen 5 up-regulated genes (*SERPINB2, IL13RA2, PLAU, IFI6, TXNIP*) and 5 down-regulated genes (*IGFBP5, CRISPLD2, THBS2, ATOH8, NNMT*) for quantitative studies.

**Figure 2:**
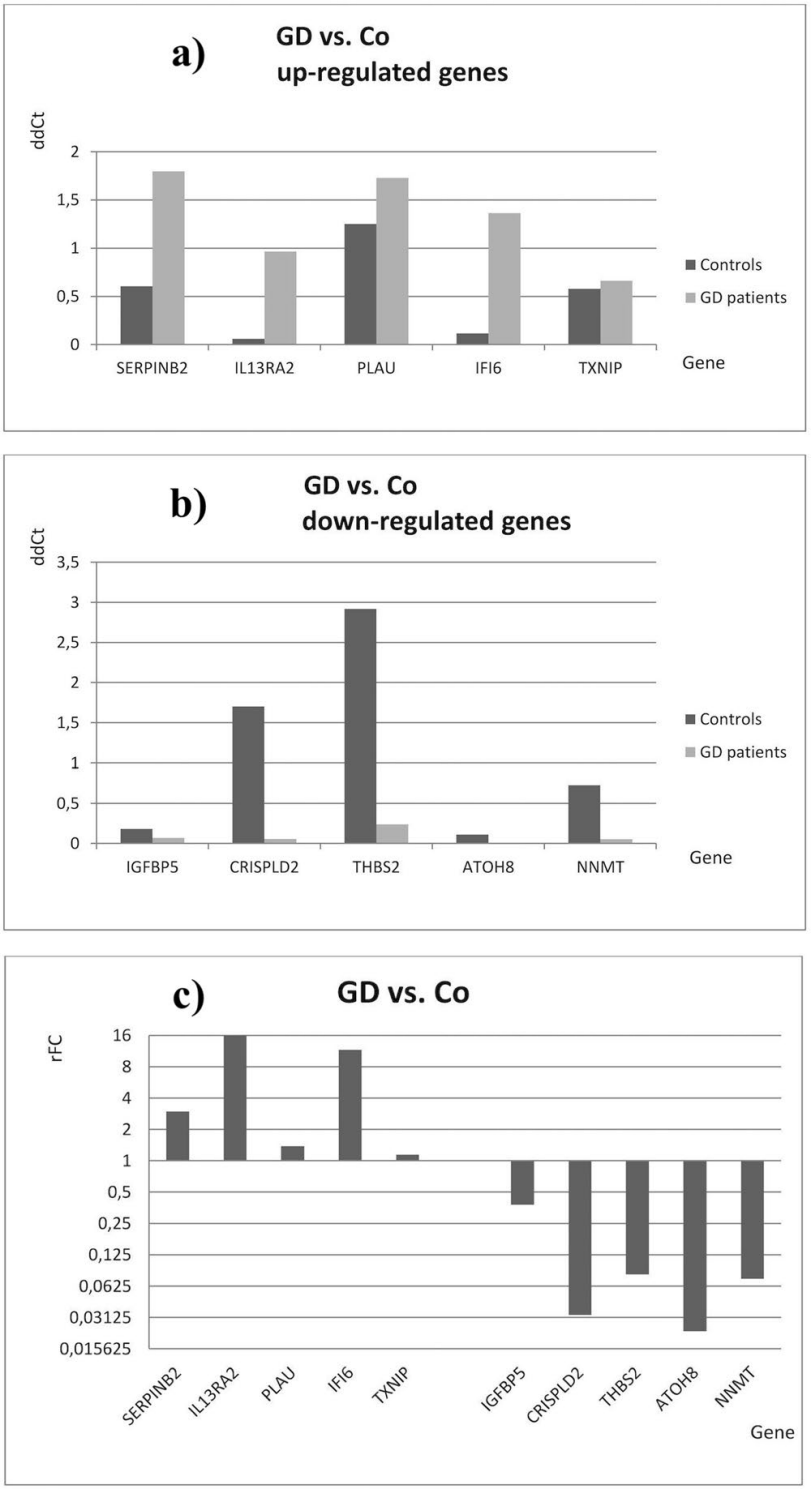
Medians of ddCt and relative fold change (rFC) values in GD patients after qRT-PCR analyses (comparison of GD patients vs. control persons). Data represent medians of ddCt obtained after qRT-PCR (n = 5). (**a**) Medians of ddCt - up-regulated genes, (**b**) medians of ddCt - down-regulated genes and relative expression (**c**) rFC (fold change of Controls) after analysis of medians of ddCt obtained in Gaucher disease (GD) patients and controls (Co). Gene expression was recognized as up-regulated when rFC was >1 and down-regulated when rFC was <1.

In the comparison of GD patients vs. controls the most pronounced relative fold change (rFC) was observed for the *IL13RA2* and *IFI6* (up-regulated expression, rFC 16 and 11, respectively) and for *ATOH8* and *CRISPLD2* (down-regulated expression, rFC 0.02 and 0.03, respectively, Fig. 2).

#### Comparison – GD patients vs. NPC patients

After microarray studies and IPA, cellular canonical pathways with most differentially changed gene expression included: Interferon Signalling, Protein Ubiquitination Pathway, Activation of IRF by Cytosolic Pattern Recognition Receptors, Glioma Invasiveness Signalling, Oncostatin M Signalling, Coagulation System, IL-17A Signalling in Fibroblasts, Prostanoid Biosynthesis, Glucocorticoid Receptor Signalling, Parkinson’s Signalling, and 64 other pathways in which FOS was involved, see Table 1, Fig. 1, Supplementary Table S4. Table 2 summarizes results obtained for genes with altered expression after microarray analysis for the comparison of GD and NPC patients.

In the next step, we have chosen 5 up-regulated genes (*MX1, TMEM158, UCHL1, ISG15, PLAU*) and 5 down-regulated genes (*PTGIS, ATOH8, MXRA5, MN1, FOXQ1*) for the qRT-PCR studies.

**Figure 3:**
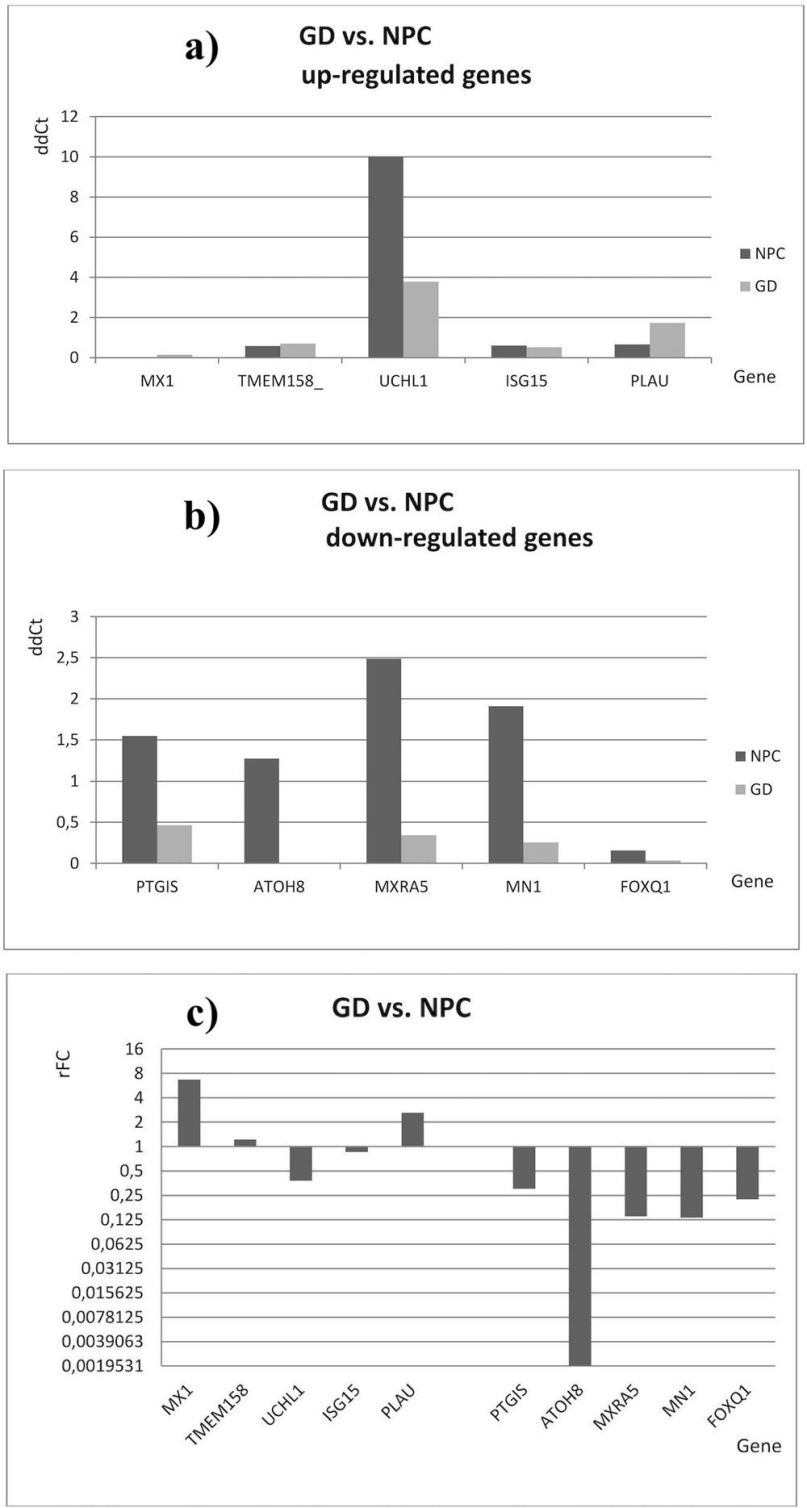
Medians of ddCt and relative fold change (rFC) values in GD patients after qRT-PCR analyses (comparison of GD patients vs. NPC patients). Data represent medians of ddCt obtained after qRT-PCR (n = 5). (**a**) Medians of ddCt - up-regulated genes, (**b**) medians of ddCt - down-regulated genes and relative expression (**c**) rFC (fold change of NPC patients) after analysis of medians of ddCt obtained in Gaucher disease (GD) and Niemann-Pick type C (NPC) patients. Gene expression was recognized as up-regulated when rFC was >1 and down-regulated when rFC was <1.

In the comparison of GD patients vs. NPC patients the most remarkable relative fold change (rFC) in expression after qRT-PCR analysis was observed for *MX1* and *PLAU* genes with up-regulated expression (rFC 6.7 and 2.6, respectively) and for *ATOH8*, *MXRA5* and *MN1* with down-regulated expression (rFC 0.02, 0.14 and 0.13, respectively), see Fig. 3.

#### Summary of microarray and qRT-PCR data analyses

**Figure 4:**
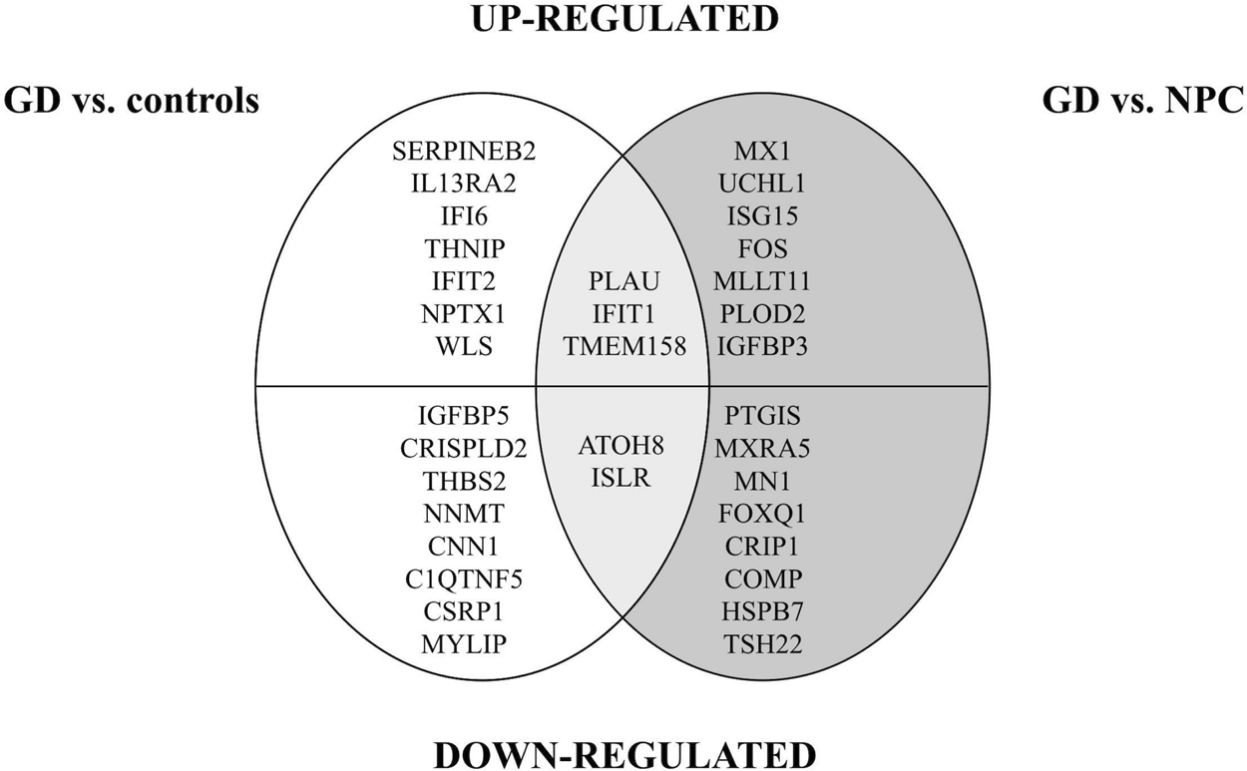
Genes with changed expression in GD patients in comparison with controls and NPC patients after microarray study.  Up- and down-regulated genes: analysis GD patients vs. controls  analysis GD vs. NPC patients  overlap. GD – Gaucher disease patients, NPC – Niemann-Pick type C patients.

Microarray experiments showed an up-regulation of *PLAU*, *IFIT1*, *TMEM158* and down-regulation of *ATOH8* and *ISLR* in GD patients in comparison to NPC patients and controls (when analysed concomitantly); see Table 2 and Fig. 4.

Generally, genes which were found up-regulated in microarray experiments were involved in PLAU signalling pathway as well as in Jak-Stat (*PLAU*) and Ras (*TMEM158*) pathways, cytokine signalling in immune system (*IFIT1*) and in neuronal survival pathway (*TMEM158*). Genes which were down-regulated were involved in the cellular response to elevated platelet cytosolic Ca^2+^ (*ISLR*) and in the processes modulating endothelial cell differentiation as well as specification and differentiation of neuronal cell lineages in the brain (*ATOH8*)^18^.

The qRT-PCR analyses indicated that in GD patients vs. controls the most pronounced expression up-regulation was found for *IL13RA2* and *IFI6* (rFC 16 and 11, respectively) whereas the highest down-regulation was observed for *ATOH8* and *CRISPLD2* (rFC 0.02 and 0.03, respectively, see Fig. 2).

When GD patients were compared to NPC patients, the expression of *MX1* and *PLAU* genes was up-regulated (rFC 6.7 and 2.6) and the expression of *ATOH8*, *MXRA5* and *MN1* was down-regulated (rFC 0.02, 0.14, and 0.13, see Fig. 3).

The protein encoded by the *IL13RA2* gene participates in cytokine signalling in immune system and Jak-Stat signalling pathway.

The protein encoded by *IFI6* gene may play a critical role in the regulation of apoptosis. Among its related pathways are type II interferon signalling (IFNG) and interferon gamma signalling^18^.

*CRISPLD2* gene encodes a protein binding heparin and glycosaminoglycans, and involved in innate immune system (especially in neutrophil degranulation process)^18,19^.

Product of the *MX1* gene is a guanosine triphosphate (GTP)-metabolizing protein that participates in the cellular antiviral response. It also enhances ER stress-mediated cell death after influenza virus infection and may regulate the calcium channel activity of TRPCs (Transient Receptor Potential-Canonical)^20^. The *MX1* gene product is involved in PI3K-Akt signalling pathway, cytokine signalling in immune system, innate immune system, immune response IFN alpha/beta signalling pathway, and interferon gamma signalling^19^.

*MXRA5* gene encodes one of the matrix-remodelling associated proteins. Protein *MXRA5* (adlican) is an adhesion proteoglycan involved in extracellular matrix (ECM) remodelling and cell-cell adhesion^21^.

The *MN1* gene product promotes maturation and normal function of calvarial osteoblasts inducing expression of the osteoclastogenic cytokine TNFSF11/RANKL. Zhang *et al*.^22^ demonstrated that the RANKL promoter was stimulated by expression of MN1 in primary osteoblasts in mice. In MN1 knock-out osteoblasts RANKL promoter activity was significantly decreased when compared with wild-type cells. The stimulation of RANKL promoter activity by MN1 supports a potential mechanism in which MN1 promotes osteoclastogenesis via increased transcription of the RANKL gene in osteoblasts^22^. MN1 may also play a role in tumour suppression^20^. *MN1* product influences expression of genes involved in haematopoiesis and can enhance as well as inhibit RAR/RXR-induced gene expression (retinoic acid receptor/retinoic x receptor)^23^. Inactivation of *MN1* may lead to certain meningiomas^24^ and potentially to leukemia^25,26^.

### Statistical analysis

The differences between ddCT values obtained for the genes studied in GD patients, NPC patients, and controls were analysed for their statistical significance.

Statistical analysis of results obtained in GD patients and controls was performed both with the Mann-Whitney test (medians) and t-Student test (means of log-transformed variables). It revealed statistically significant differences in the ddCt values in the case of *IL13RA2* (up-regulated), *THBS2*, and *NNMT* genes (down-regulated); see Supplementary Table S10. In the two groups of individuals the ranges, medians, 1^st^ and 3^rd^ quartile values did not overlap. This suggests that *IL13RA2*, *THBS2*, and *NNMT* proteins could be useful biomarkers for the differentiation of GD patients from healthy controls.

Similar analysis was performed for ddCt values obtained in GD and NPC patients. Both Mann-Whitney test and t-Student test revealed statistically significant differences in expression of *UCHL1* (up-regulated), *ATOH8*, *MN1*, and *FOXQ1* genes (down-regulated) in GD vs. NPC patients; see Supplementary Table S11. Since the ranges, medians, 1^st^ and 3^rd^ quartile values do not overlap between the two groups we propose that *UCHL1*, *ATOH8*, *MN1*, and *FOXQ1* could be good biomarkers for differentiation of GD from NPC.

**Figure 5:**
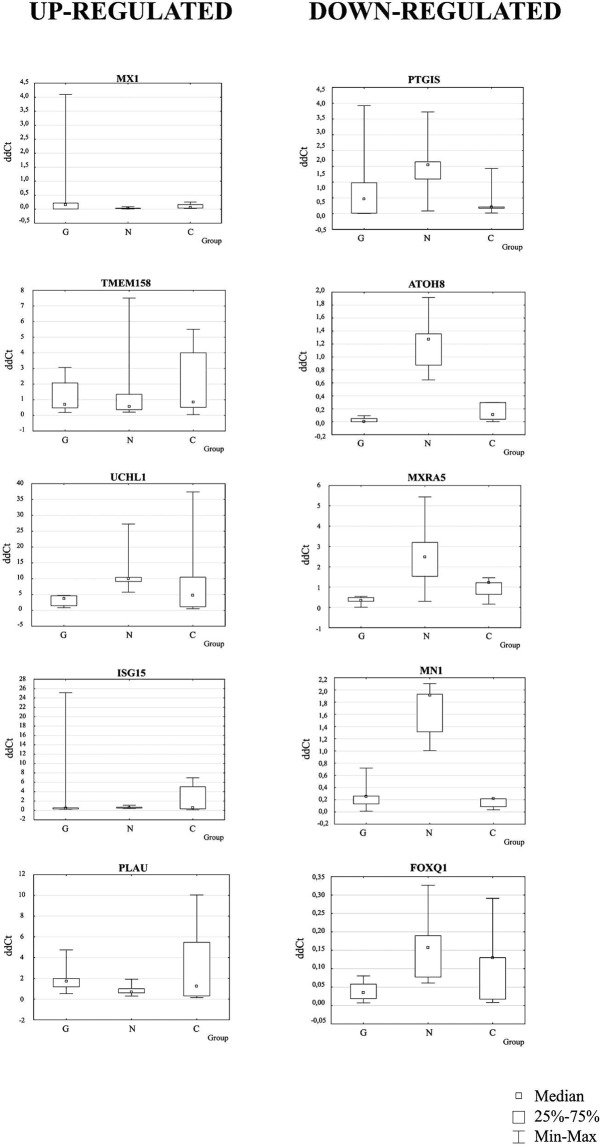
Statistically significant differences in the ddCt values for up- and down-regulated genes. Analysis of results obtained after qRT-PCR analyses in Gaucher disease (GD) patients, Niemann-Pick type C (NPC) patients, and control persons. Data are presented as medians of ddCt, interquartile ranges from first to third quartile, and minimum and maximum values. G – GD patients, N – NPC patients, C – control persons

Additionally, one-way Anova and Kruskal-Wallis Anova were performed for the comparison of ddCt values in GD patients, NPC patients and controls, simultaneously. Statistically significant differences in ddCt values were observed in the case of *ATOH8* and *MN1* genes. Thus, proteins encoded by these genes could be good biomarkers for the differentiation of NPC patients from both GD patients and controls, but not GD patients from controls; see Table 3 and Fig. 5. Whereas it is possible that incidentally we have found potential biomarkers for the diagnosis of NPC these results should be verified.

**Table 3:** Statistically significant differences in the ddCt values - analysis of results obtained in GD patients, NPC patients, and control persons.

Gene	ddCt	ddCt	ddCt	P ANOVA Kruskal-Wallis	P One-way ANOVA^A^
	GD patients	NPC patients	controls		
	n = 5	n = 5	n = 5		
*MX1*	0.1530 [0.0067–0.2171]	0.0229 [0.0213–0.0333]	0.0584 [0.0397–0.1634]	0.265	0.464
*TMEM158*	0.7064 [0.4861–2.0786]	0.5789 [0.3727–1.3480]	0.8509 [0.5185–4.0034]	0.932	0.999
*UCHL1*	3.7895 [1.4849–4.6394]	10.0139 [9.1342–10.4285]	4.7071 [1.1682–10.4798]	**0.077** ^B^	0.161
*ISG15*	0.5225 [0.3093–0.5590	0.6090 [0.4881–0.6703]	0.5831 [0.3475–5.0640]	0.810	0.873
*PLAU*	1.7297 [1.1790–1.9834]	0.6592 [0.5987–0.9978]	1.2501 [0.3067–5.4708]	0.512	0.605
*PTGIS*	0.4643 [0.0166–0.9778]	1.5476 [1.0997–1.6428]	0.2121 [0.1709–0.2121]	0.275	0.340
*ATOH8*	0.0025 [0.0016–0.0487]	1.2724 [0.8737–1.3571]	0.1098 [0.0393–0.2956]	**0.048** ^C^	**0.001** ^F^
*MXRA5*	0.3414 [0.3003–0.4857]	2.4863 [1.5311–3.2034]	1.2144 [0.6442–1.2144]	**0.055** ^D^	**0.049** ^G^
*MN1*	0.2538 [0.1325–0.2612]	1.9104 [1.3138–1.9294]	0.2171 [0.0881–0.2171]	**0.008** ^E^	**0.003** ^H^
*FOXQ1*	0.0349 [0.0187–0.0578]	0.1565 [0.0772–0.1897]	0.1301 [0.0172–0.1301]	0.105	0.135

Data are presented as medians and interquartile ranges from first to third quartile.

^A^Variables were logarithmically transformed in statistical analysis.

^B^p = 0.071 (borderline significant) GD vs. NPC group (ANOVA Kruskal-Wallis post-hoc test).

^C^p = 0.004 GD vs. NPC group (ANOVA Kruskal-Wallis post-hoc test).

^D^p = 0.049 GD vs. NPC group (ANOVA Kruskal-Wallis post-hoc test).

^E^p = 0.059 (borderline significant) GD vs. NPC group; p = 0.009 NPC vs. Control group (ANOVA Kruskal-Wallis post-hoc test).

^F^p = 0.0003 GD vs. NPC group; p = 0.034 GD vs. Control group; p = 0.015 NPC vs. Control group (ANOVA LSD post-hoc test).

^G^p = 0.017 GD vs. NPC group (ANOVA LSD post-hoc test).

^H^p = 0.0003 GD vs. NPC group; p = 0.002 NPC vs. Control group (ANOVA LSD post-hoc test).

Bold values are the significant or borderline significant results.

### Networks

Microarray data analysis with the IPA software revealed 15 networks of molecules in interactions for the differentially expressed genes in GD vs. controls (see Supplementary Fig. S5 and Supplementary Table S6) and 17 networks of molecules in interactions for the differentially expressed genes in GD vs. NPC (Supplementary Table S7 and Supplementary Fig. S8).

Differentially expressed genes in GD patients in comparison to controls and NPC patients were involved in the following networks:

- Infectious Diseases, Cell Signalling, Cell Morphology (*IFI6*)
- Nervous System Development and Function, Developmental Disorder, Endocrine System Disorders (*TMEM158*)
- Antimicrobial Response, Inflammatory Response, Neurological Disease (*IL13RA2*)
- Cardiovascular System Development and Function, Organismal Injury and Abnormalities, Cancer (*PLAU*)
- Skeletal and Muscular System Development and Function, Organismal Injury and Abnormalities, Respiratory Disease (*IFIT1*)
- Cell-To-Cell Signaling and Interaction, Cell Cycle, Renal and Urological System Development and Function (*IFI6*)
- Cellular Compromise, Cell Cycle, Cellular Development (*ISLR*)
- Psychological Disorders, Cardiac Thrombosis, Cardiovascular Disease (*ATOH8*)
- Cellular Movement, Cellular Development, Cellular Growth and Proliferation (*CRISPLD2*)
- Connective Tissue Disorders, Skeletal and Muscular Disorders, Cancer (*PLAU*)
- Gene Expression, Cardiovascular System Development and Function, Embryonic Development (*MN1*)
- Antimicrobial Response, Inflammatory Response, Cell Signaling (*IFIT1, MX1*)
- Cell Morphology, Cellular Assembly and Organization, Cell Death and Survival (*TMEM158*)
- Cellular Movement, Hematological System Development and Function, Immune Cell Trafficking (*ATOH8*)
- Cell Cycle, Connective Tissue Development and Function, Cancer (*ISLR*)
- Hereditary Disorder, Neurological Disease, Organismal Injury and Abnormalities (*MXRA5*).

**Figure 6:**
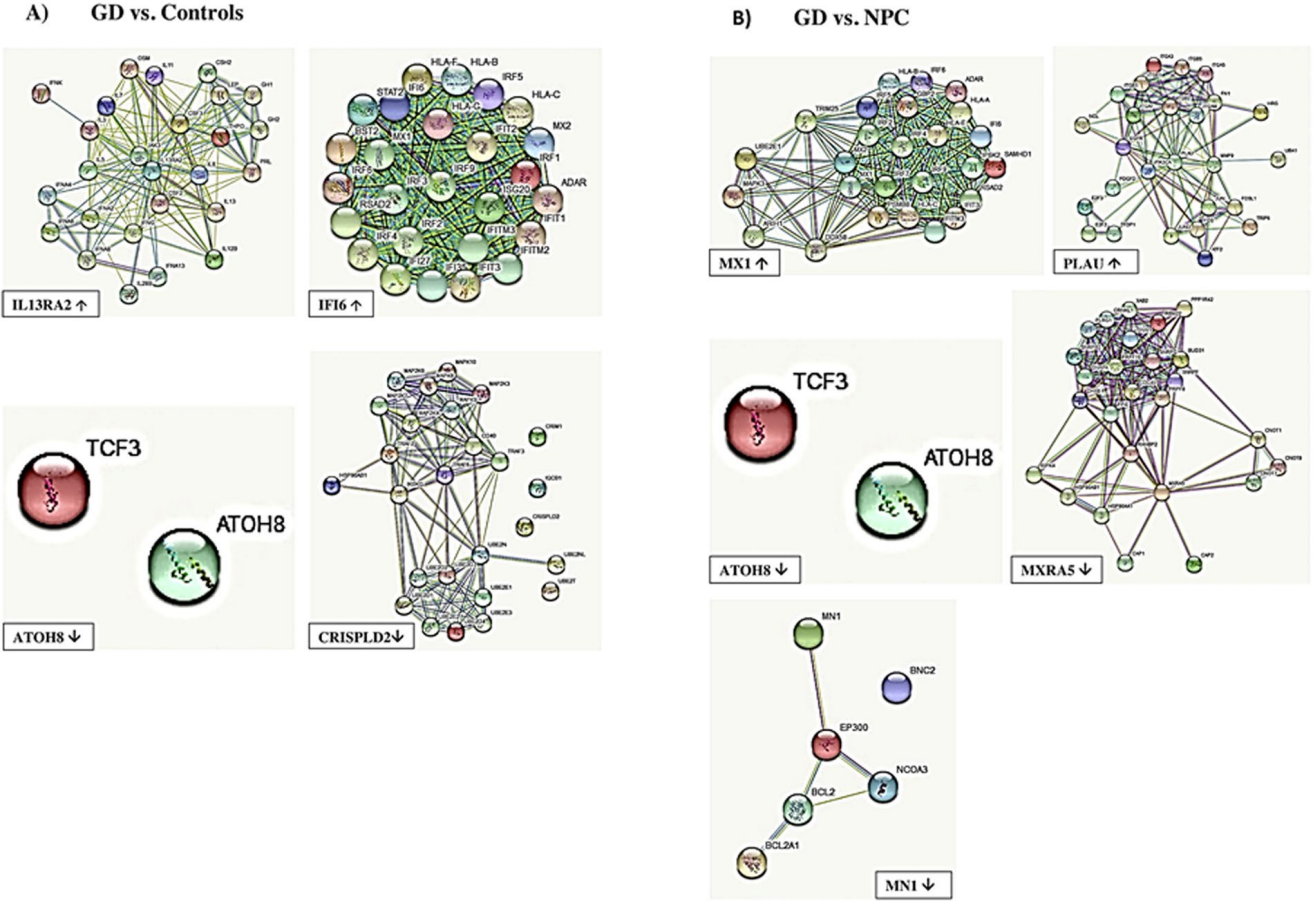
Interacting proteins for most differentially expressed genes after qRT-PCR studies in GD patients using STRING interaction network. (**A**) Analysis: Gaucher disease (GD) patients vs. control persons. (**B**) Analysis: Gaucher disease (GD) patients vs. Niemann-Pick type C (NPC) patients. Figures represent network of proteins interacting with products of most differentially expressed genes: (1) protein products of most up-regulated genes - IL13RA2 and IFI6 for analysis (**A**), and MX1 and PLAU for analysis (**B**) (2) protein products of most down-regulated genes – ATOH8 and CRISPLD2 for analysis (**A**), and ATOH8 and MXRA5 for analysis (**B**). For details, please see STRING: functional protein association networks, https://string-db.org.

For top genes whose expression was different in GD vs. controls and NPC patients we have created networks of interacting proteins using the STRING interaction network database^27^, see Fig. 6.

Genes with up-regulated expression were involved in the following cellular pathways (on the basis of results obtained from STRING database): Jak-STAT signalling pathway, cytokine-cytokine receptor interaction, PI3K-Akt signalling pathway, type I interferon signalling pathway, cellular response to type I interferon, cellular response to cytokine stimulus, innate immune response, cytokine-mediated signalling pathway, cellular response to cytokine stimulus, integrin-mediated signalling pathway, angiogenesis, cell-matrix adhesion, regulation of actin cytoskeleton, pathways in cancer, proteoglycans in cancer.

Genes with down-regulated expression were involved in the following cellular pathways (on the basis of results obtained from STRING database): E-box binding, endothelial cell differentiation through NOS3, specification and differentiation of neuronal cell lineages in the brain, positive regulation of immune response, Toll-like receptor signalling pathway, TNF signalling pathway, Ubiquitin mediated proteolysis, Spliceosome, RNA degradation, intrinsic apoptotic signalling pathway in response to DNA damage, NF-kappa B signalling pathway.

## Discussion

Results obtained in this multicentre study confirm the significant role of inflammation in the pathogenesis of Gaucher disease in humans which is similar to observations in animal models of GD. Products of genes with up-regulated expression in GD were engaged in cytokine signalling in immune system and the Jak-Stat signalling pathway (*IL13RA2*), autophagy and apoptosis (*SERPINB2*, *IFI6* whose product additionally participates in a related pathway, i.e. type II interferon signalling (IFNG) and interferon gamma signalling)^18^ or ER stress-mediated cell death after influenza virus infection (*MX1*). MX1 protein may also regulate the calcium channel activity of TRPCs (Transient Receptor Potential-Canonical), is involved in PI3K-Akt signalling pathway, cytokine signalling in immune system, innate immune system, immune response IFN alpha/beta signalling pathway, interferon gamma signalling^19,20^. Products of genes with down-regulated expression in GD were involved in immune response (neutrophil degranulation – *CRISPLD2*), cell-to-cell and cell-to-matrix interactions and matrix remodelling (*THBS2*, *MXRA5*), PI3K-Akt signalling pathway (*THBS2*), maturation and normal function of calvarial osteoblasts including expression of the osteoclastogenic cytokine as well as in meningiomas and leukemia (*MN1*), in the cellular response to elevated platelet cytosolic Ca^2+^ (*ISLR*), in the processes modulating endothelial cell differentiation and specification and differentiation of neuronal cell lineages in the brain (*ATOH8*)^18^.

In general, the following genes distinguished GD from both NPC patients and controls: *PLAU*, *IFIT1*, *TMEM158* (up-regulated in GD), *ATOH8* and *ISLR* (down-regulated in GD). Results of gene expression profiling in NPC patients vs. controls will be presented in another article.

Abnormal immune system function causes clinical symptoms in patients affected with GD but also with Fabry disease, mucopolysaccharidoses, gangliosidoses and Niemann-Pick disease (A/B and C types). The abnormalities of the immune system include the increased production of proinflammatory cytokines (GD, Fabry disease), autoantibody production (GD, Fabry, gangliosidoses) as well as disturbed microglia activation (Niemann-Pick disease)^28^.

It has already been shown that cytokines, chemokines and other molecules involved in inflammation are associated with macrophage activation via the classic (M1 phenotype) and alternative (M2 phenotype) pathways^29,30^. In macrophages of GD patients an increased secretion of the proinflammatory IL-1beta due to the activation of caspase-1 and IL-1 following the formation of the inflammasome has been found^30^. There are also reports of increased levels of other cytokines and chemokines in GD patients and GD animal models (IL-1Ra, sIL-2R, IL-6, IL-8, IL-10, IL-18HGF, MCSF, MIP-1, CCL18, sCD14, TGF-beta1, TNF-alfa)^31–35^.

Transcriptome analyses using microarrays or mRNA sequencing revealed that in a *Gba1* mutant mouse model of GD expression of the genes involved in the Jak-Stat pathway was enhanced after ERT (enzyme replacement therapy) with both imiglucerase and velaglucerase indicating an important role of anti-inflammatory actions of Jak-Stat pathway genes products^36^.

Global gene expression analyses in GD mouse models implicated IFNgamma-regulated pro-inflammatory and IL-4-regulated anti-inflammatory networks^34^.

In another study of a mouse model of neuronopathic GD expression profile analysis was performed in affected brain tissue at a pre-symptomatic stage. Results showed that inflammatory genes were up-regulated with the gene expression signature significantly enriched in interferon signalling genes^37^. Interferon beta was elevated in neurons while interferon-stimulated genes products were increased mainly in microglia. Authors concluded that type I interferon response was involved in the pathomechanism of neuronopathic form of GD most probably via the effect of accumulated GlcCer on interferon induction and secretion in neuron followed by activation of microglia by binding to IFNAR [Ifnα/β receptor (IFNAR)], phosphorylation of STAT [(JAK)-signal transducer and activator of transcription (STAT)], and induction of ISGs [type I IFN-stimulated genes (ISGs)].

Several genes and proteins involved in inflammatory processes have been also investigated. For example, pro-inflammatory kinase p38 which is normally suppressed by ceramide, has been found activated in lung and liver tissues in mouse models of three clinical types of GD and exclusively in brains of neuronopathic GD mice. Additionally, p38-inducible proinflammatory cytokines (e.g. IL-6) were up-regulated in mouse models and in human GD fibroblasts, in which p38 was also activated^38^.

In spleen samples of GD patients up-regulation of cysteine proteinases – cathepsins B, K, and S - as well as chemokine CCL-18, IL6, and gpNMB protein were observed^17^.

In GD patients, increased incidence of neoplasia (especially myeloma) was described and attributed to a disturbed profile of immunoglobulins^39,40^. In our study, the inhibition of the *MN1* gene expression was observed, which may result in certain meningiomas^24^ and leukemia^25,26^.

Our second aim was to identify novel biomarker(s) due to an emerging need for quick and reliable tools for primary detection of GD (allowing early introduction of therapy) as well as for differentiation of GD from NPC. So far, the most common biomarkers to monitor therapeutic response in GD patients are chitotriosidase and CCL-18. However, one limitation in the use of chitotriosidase activity is the presence of genetic variants in its gene (*CHIT1*; MIM #600031.0001) which result in lack or reduced enzymatic activity. In particular, the most common variant, the 24 bp duplication in exon 10 of the *CHIT1* gene, was reported with allele frequencies of >0.20 and >0.50 in Europeans and Asians, respectively^41^. GD patients with the homozygous 24 bp *CHIT1* duplication have chitotriosidase deficiency which renders the measurement of activity of this enzyme uninformative. The use of CCL-18, the alternative biomarker, may also be misleading as its increased levels could be caused by chronic inflammation resulting from other causes than lysosomal storage^17,42,43^.

Recently, glucosylsphingosine (lyso-Gl-1) has been used for primary detection of GD and for monitoring efficacy of a therapy^3,4,44,45^.

Statistical analysis of results obtained in our experiments indicated that *IL13RA2*, *THBS2* and *NNMT* proteins could be useful biomarkers for the differentiation of GD patients from controls (*IL13RA2* is up-regulated, while *THBS2* and *NNMT* are down-regulated). Proteins encoded by *UCHL1*, *ATOH8*, *MN1* and *FOXQ1* could to be biomarkers for differentiating GD from NPC (*UCHL1* is up-regulated and *ATOH8*, *MN1* and *FOXQ1* are down-regulated). Due to financial limitations and difficulties in obtaining samples from GD patients who are not treated by enzyme replacement therapy we did not perform analyses in blood aimed at conclusive assessment of diagnostic value of these potential novel biomarkers in comparison with existing ones - chitotriosidase activity, levels of lyso-Gl1, lyso-SM, and lyso-SM-509. Clearly, this is a plan for future experiments in co-operation with other medical centres.

Recently, by proteome analysis, increased amounts of the gpNMB protein were found in plasma and splenic cells from cells GD patients as well as in a mouse model of GD^46^. Murugesan observed over 15-fold elevation of gpNMB protein in sera of untreated GD patients and suggested the utility of this protein as a biomarker useful both for diagnosis and for monitoring of GD treatment^47^. gpNMB is most probably involved in lysosomal stress and in the degradation of cellular debris, and macroautophagy^48^. In our material the gpNMB protein was not significantly elevated which can be explained by different source of material and the number of the examined individuals.

Summarizing, gene expression changes associated with GD have not been studied in humans apart from a single report on global gene expression analysis on splenic tissue from 4 GD patients^17^. Therefore, we believe that results presented in this multicentre study are an important contribution to understanding the pathological processes occurring in humans affected with GD. In general, our results confirm and emphasize the inflammatory character of changes taking place in affected cells and tissues. This indicates that further studies on novel therapeutics for GD should consider anti-inflammatory agents.

## Materials and Methods

### Patients’ cell lines

Skin fibroblasts were taken from 5 healthy individuals (2 males and 3 females), 5 patients with GD (2 patients suffering from the type 1 GD, 2 – with type 2 GD, and 1 – with type 3 GD; 3 males and 2 females), and 5 patients with NPC caused by mutations in the *NPC1* gene (4 patients with infantile type and 1 – with juvenile type NPC; 1 male and 4 females). GD was confirmed by the deficient beta-glucocerebrosidase activity in blood leukocytes and elevated chitotriosidase activity in plasma. In three patients (one with type 1, one with type 2, and one with type 3 GD) the molecular background of the disease was established by mutation analysis in the *GBA1* gene (Table 4). NPC patients were diagnosed by the test with filipin and confirmed by mutation analysis in the *NPC1* and *NPC2* genes. Skin fibroblasts lines from GD and controls originated from the collection of Institute of Psychiatry and Neurology, Department of Genetics (Warsaw, Poland), while the NPC patients’ cell lines originated from the ‘Cell line and DNA Biobank from patients affected by Genetic Diseases’ located at G. Gaslini Institute (Genoa, Italy)^49^ and member of the Telethon Network of Genetic Biobanks (http://biobanknetwork.telethon.it/).

**Table 4:** Characteristics of Caucasian patients with Gaucher disease (GD) and Niemann-Pick type C (NPC) disease examined in this study.

Patient	Disease type	Age at skin biopsy	Sex	Gene	Genotype*
1	GD 1	43 yr.	M	*GBA*	[N370S] + [L444P]
2	GD 2	3 mo.	F	*GBA*	[D399N] + [L444P]
3	GD 3	1 yr. 9 mo.	F	*GBA*	[D448G] + [R202X]
4	GD 2	8 mo.	M		n.a.
5	GD 1	52 yr.	M		n.a.
1	NPC, infantile	2 yr.	F	*NPC1*	p.[C31WfsX26] + [C31WfsX26]
2	NPC, infantile	5 yr.	F	*NPC1*	p.[H512R] + [Y1019C]
3	NPC, infantile	newborn	F	*NPC1*	p.[T1205NfsX53] + [T1205NfsX53]
4	NPC, juvenile	10 yr.	F	*NPC1*	p.[L1191F] + [L1191F]
5	NPC, infantile	4 yr.	M	*NPC1*	p.[F284LfsX26] + [F284LfsX26]

F – female, M – male, n.a. = data not available.

* *NPC1* mutations are described according to the HGVS recommended nomenclature (http://varnomen.hgvs.org/). NPC1 amino acid numbers are derived from GenBank accession no. NP_000262).

Note that the *GBA* mutations at the protein level are described following the traditional nomenclature within the Gaucher field, which considers amino acid 1 the first amino acid after the signal peptide (GenBank accession no. M16328.1). According to current HGVS recommended nomenclature, ascribing the A of the first ATG translational initiation codon as nucleotide + 1, 39 amino acids should be added.

All cell lines were cultured in DMEM (Thermo Fisher Scientific Inc.) supplemented with 10% fetal bovine serum (FBS) and 1% penicillin/streptomycin solution (Sigma-Aldrich Co. LLC., St. Louis, USA) in a humidified atmosphere containing 5% CO_2_ at 37 °C. Cells were harvested after trypsinization when the culture was confluent, immediately immersed in RNA Later (Qiagen), and stored at −70 °C before further procedures.

### Ethical aspects

Following ethical guidelines, all cell and nucleic acid samples were obtained for analysis and stored with the patients’ (and/or a family member’s) written informed consent. The protocol and procedures of this study were accepted by the local Bioethics Committee at the Institute of Psychiatry and Neurology (Warsaw, Poland). All experiments were performed in accordance with relevant guidelines and regulations.

### RNA isolation

Total RNA samples were isolated from the cultured skin fibroblasts with the use of MagNa Pure Compact RNA Isolation Kit (Roche Applied Science, Mannheim, Germany). Quantity and quality of RNA samples were assessed using the NanoDrop instrument (Thermo Scientific) and evaluated with the RNA 6000 Nano Assay on the Agilent 2100 Bioanalyser (Agilent Technologies Inc., USA).

### Microarray analyses

Gene expression profiles were analysed with HumanHT-12 v4.0 Expression BeadChip Kit (Illumina Inc., USA). Biotin-labeled cRNA samples for hybridization were prepared according to Illumina’s recommended sample labeling procedure. The hybridization cocktail containing the fragmented and labeled cRNA was hybridized to HumanHT-12 v4.0 Expression BeadChip using TargetAmp™-Nano Labeling Kit for Illumina® Expression BeadChip® which enables whole-genome expression studies. Each array on the HumanHT-12 v4 Expression BeadChip targets more than 47,000 probes on a single BeadChip, derived from National Center for Biotechnology Information Reference Sequence (NCBI) RefSeq Release 38 (November 7, 2009) and other sources. HumanHT-12 v. 4 Expression BeadChips arrays were scanned with the iScan system.

Row data obtained after microarray experiments were then analysed with the Partek Genomic Suite v 6.6 software with the use of GCRMA (GC Robust Multiarray Averaging). During this step background correction was applied based on the global distribution of the PM (perfect match) probe intensities and the affinity for each of the probes (based on their sequences) was calculated. Further, the probe intensities were quantile normalized^50^ and median polish summarization to each of the probe sets was applied. Then the qualitative analysis was performed, e.g. Principal Component Analysis, in order to identify outliers and artefacts on the microarray. After quality check the Analysis of Variance (ANOVA) was performed on the data, which allowed to create lists of significantly and differentially expressed genes between the biological variants. The selected lists were subjected to cluster analysis in order to find genes and samples with similar profiles, with the use of Hierarchical Clustering algorithm. The results were interposed onto the publicly available data bases containing information about gene functions, e.g. Ingenuity Pathway Analysis program (Qiagen Bioinformatics) or Gene Ontology.

Genes with the expression fold change (FC) greater than 2.0 or lower than −2.0 were considered as being significantly differentially expressed and further analysed by qRT-PCR.

### Quantitative Real-Time PCR (qRT-PCR)

To verify the selected sets of genes which were up- or down-regulated after microarray studies we performed qRT-PCR to check the level of expression. cDNA samples were obtained after reverse transcription with Maxima First Strand cDNA Synthesis Kit for RT-qPCR (Thermo Scientific, Thermo Fisher Scientific Inc.), according to the manufacturer’s instructions. qRT-PCR was carried out with DyNAmo ColorFlash Probe qPCR Kit (Thermo Scientific) and TaqMan Gene Expression probes for up- or down regulated genes (Applied Biosystems) and the reactions were performed in the PikoReal™ Real-Time PCR System (Thermo Scientific). In Supplementary Table S9 genes and probes used in qRT-PCR analyses are listed. The thermal cycling parameters were following: 95 °C for 7 min, 50 cycles of 95 °C for 10 sec., 60 °C for 30 sec. followed by incubation at 60 °C for 30 sec. and at 20 °C for 10 sec. Each cDNA sample was analysed in 3 solutions (16, 32, and 64 ng) and each solution was analysed in duplicates.

Expression values were normalized against two housekeeping control genes - *PUM1* and *SDHA* (E^−ΔΔCt^ method, where E means amplification efficiency of the reaction). Results are presented as medians of the relative fold change (rFC), which is a ratio of normalized mRNA level of the analysed gene expression in GD patients in comparison to control individuals or NPC patients.

### Statistical analysis

Statistical analysis of the normalized gene expression data after qRT-PCR (ddCt) was performed with Statistica version 13 (StatSoft, Poland). Normality of data distribution was checked by the Kolmogorov-Smirnov and Shapiro-Wilk tests. Because the variables were skewed, they were natural log-transformed to approximate a normal distribution before statistical analysis with parametric or nonparametric tests. Variables were presented as medians with interquartile ranges (IQR). Differences between two groups (GD vs. controls and GD vs. NPC) were tested using nonparametric Mann-Whitney test and Student t-test. Differences between three groups (GD, NPC and controls) were tested using a nonparametric Kruskal-Wallis analysis of variance (ANOVA) followed by post hoc test for multiple comparisons and one-way Analysis of Variance (ANOVA) followed by Least Significant Difference (LSD) post-hoc test. P-values lower than 0.05 were considered as statistically significant.

For qRT-PCR experiments, a relative fold change (rFC) greater than 1.0 was considered as a relevant criterion for genes with significantly up-regulated expression, while an rFC between 0.0 and 1.0 indicated genes with down-regulated expression.

### Network and functional correlation analysis

Data of significantly differentially expressed genes in GD patients and control individuals as well as in GD and NPC patients were analysed through the use of IPA (QIAGEN Inc., https://www.qiagenbioinformatics.com/products/ingenuity-pathway-analysis)^51^ and STRING (https://string-db.org) programs and databases.

## Supplementary information

Supplementary information

## Data Availability

All data underlying the findings described in this article are fully available without restriction at the NCBI’s Gene Expression Omnibus (GEO, http: www.ncbi.nlm.nih.gov/geo, GEO Series accession number GSE124283) or are within the article and its Supplementary Information files. If any additional questions arise authors will share any requested documents, methods or data.

## References

[1] Grabowski GA (2008). Phenotype, diagnosis, and treatment of Gaucher’s disease. Lancet..

[2] Stirnemann J (2012). The French Gaucher’s disease registry: Clinical characteristics, complications and treatment of 562 patients. Orphanet J. Rare Dis..

[3] Rolfs A (2013). Glucosylsphingosine Is a Highly Sensitive and Specific Biomarker for Primary Diagnostic and Follow-Up Monitoring in Gaucher Disease in a Non-Jewish, Caucasian Cohort of Gaucher Disease Patients. PLoS One..

[4] Murugesan V (2016). Glucosylsphingosine is a key Biomarker of Gaucher Disease. Am. J. Hematol..

[5] Pàmpols T (1999). Neuronopathic juvenile glucosylceramidosis due to sap-C deficiency: clinical course, neuropathology and brain lipid composition in this Gaucher disease variant. Acta Neuropathol. (Berl)..

[6] Qi X, Grabowski GA (2001). Molecular and cell biology of acid beta-glucosidase and prosaposin. Prog. Nucleic Acid Res. Mol. Biol..

[7] Tylki-Szymańska A (2007). Non-neuronopathic Gaucher disease due to saposin C deficiency. Clin. Genet..

[8] Goker-Alpan O (2003). Phenotypic continuum in neuronopathic Gaucher disease: an intermediate phenotype between type 2 and type 3. Journal of Pediatrics..

[9] Bultron G (2010). The risk of Parkinson’s disease in type 1 Gaucher disease. J. Inherit. Metab. Dis..

[10] Lal TR, Sidransky E (2017). The Spectrum of Neurological Manifestations Associated with Gaucher Disease. Diseases..

[11] Dekker N (2011). Elevated plasma glucosylsphingosine in Gaucher disease: Relation to phenotype, storage cell markers, and therapeutic response. Blood..

[12] Mistry PK (2014). Glucocerebrosidase 2 gene deletion rescues type 1 Gaucher disease. Proc. Natl. Acad. Sci. USA.

[13] Taguchi YV (2017). Glucosylsphingosine Promotes α-Synuclein Pathology in Mutant GBA-Associated Parkinson’s Disease. J Neurosci..

[14] Hong YB, Kim EY, Jung SC (2004). Down-regulation of Bcl-2 in the fetal brain of the Gaucher disease mouse model: A possible role in the neuronal loss. J. Hum. Genet..

[15] Matloubian M (2004). Lymphocyte egress from thymus and peripheral lymphoid organs is dependent on S1P receptor 1. Nature.

[16] Fuller M (2008). Glucosylceramide accumulation is not confined to the lysosome in fibroblasts from patients with Gaucher disease. Mol Genet Metab..

[17] Moran MT (2000). Pathologic gene expression in Gaucher disease: up-regulation of cysteine proteinases including osteoclastic cathepsin K. Blood..

[18] GeneCards: https://www.genecards.org.

[19] Reactome Pathway Database: https://reactome.org.

[20] UniProtKB/Swiss-Prot: www.uniprot.org.

[21] Xiong D (2012). Exome sequencing identifies MXRA5 as a novel cancer gene frequently mutated in non-small cell lung carcinoma from Chinese patients. Carcinogenesis..

[22] Zhang X (2009). Meningioma 1 is required for appropriate osteoblast proliferation, motility, differentiation, and function. J Biol Chem..

[23] Meester-Smoor MA (2008). MN1 affects expression of genes involved in hematopoiesis and can enhance as well as inhibit RAR/RXR-induced gene expression. Carcinogenesis..

[24] Lekanne Deprez RH (1995). Cloning and characterization of MN1, a gene from chromosome 22q11, which is disrupted by a balanced translocation in a meningioma. Oncogene..

[25] Buijs A (1995). Translocation (12;22) (p13;q11) in myeloproliferative disorders results in fusion of the ETS-like TEL gene on 12p13 to the MN1 gene on 22q11. Oncogene..

[26] Grosveld GC (2007). MN1, a novel player in human AML. Blood Cells Mol. Dis..

[27] STRING: https://string-db.org

[28] Rigante D, Cipolla C, Basile U, Gulli F, Savastano MC (2017). Overview of immune abnormalities in lysosomal storage disorders. Immunol. Lett..

[29] Mucci JM (2015). Proinflammatory and proosteoclastogenic potential of peripheral blood mononuclear cells from Gaucher patients: Implication for bone pathology. Blood Cells Mol. Dis..

[30] Aflaki E (2016). Lysosomal storage and impaired autophagy lead to inflammasome activation in Gaucher macrophages. Aging Cell..

[31] Yoshino M (2007). Roles of specific cytokines in bone remodeling and hematopoiesis in Gaucher disease. Pediatr. Int..

[32] van Breemen MJ (2007). Increased plasma macrophage inflammatory protein (MIP)-1alpha and MIP-1beta levels in type 1 Gaucher disease. Biochim. Biophys. Acta..

[33] de Fost M (2008). Immunoglobulin and free light chain abnormalities in Gaucher disease type I: data from an adult cohort of 63 patients and review of the literature. Ann. Hematol..

[34] Xu YH (2011). Global gene expression profile progression in Gaucher disease mouse models. BMC Genomics..

[35] Pandey MK, Grabowski GA (2013). Immunological Cells and Functions in Gaucher *Disease*. Crit. Rev. Oncog..

[36] Dasgupta N (2013). Gaucher Disease: Transcriptome Analyses Using Microarray or mRNA Sequencing in a Gba1 Mutant Mouse Model Treated with Velaglucerase alfa or Imiglucerase. PLoS One..

[37] Vitner EB (2016). Induction of the type I interferon response in neurological forms of Gaucher disease. J. Neuroinflammation..

[38] Kitatani K (2015). Activation of p38 Mitogen-Activated Protein Kinase in Gaucher’s Disease. PLoS One..

[39] Arends M, van Dussen L, Biegstraaten M, Hollak CE (2013). Malignancies and monoclonal gammopathy in Gaucher disease; a systematic review of the literature. Br. J. Haematol..

[40] Rosenbloom BE (2005). Gaucher disease and cancer incidence: A study from the Gaucher Registry. Blood..

[41] Lee P, Waalen J, Crain K, Smargon A, Beutler E (2007). Human Chitotriosidase Polymorphisms G354R and A442V Associated with Reduced Enzyme Activity. Blood Cells Mol. Dis..

[42] Boot RG (2004). Marked elevation of the chemokine CCL18/PARC in Gaucher disease: a novel surrogate marker for assessing therapeutic intervention. Blood..

[43] Deegan PB (2005). Clinical evaluation of chemokine and enzymatic biomarkers of Gaucher disease. Blood Cells Mol. Dis..

[44] Elstein D (2017). Reductions in glucosylsphingosine (lyso-Gb1) in treatment-naïve and previously treated patients receiving velaglucerase alfa for type 1 Gaucher disease: Data from phase 3 clinical trials. Mol. Genet. Metab..

[45] Tylki-Szymańska A, Szymańska-Rożek P, Hasiński P, Ługowska A (2018). Plasma chitotriosidase activity versus plasma glucosylsphingosine in wide spectrum of Gaucher disease phenotypes - A statistical insight. Mol. Genet. Metab..

[46] Kramer G (2016). Elevation of glycoprotein nonmetastatic melanoma protein B in type 1 Gaucher disease patients and mouse models. FEBS Open Bio..

[47] Murugesan V (2018). Validating glycoprotein non-metastatic melanoma B (gpNMB, osteoactivin), a new biomarker of Gaucher disease. Blood Cells Mol. Dis..

[48] Li B (2010). The melanoma-associated transmembrane glycoprotein Gpnmb controls trafficking of cellular debris for degradation and is essential for tissue repair. FASEB J..

[49] Filocamo M (2014). Cell Line and DNA Biobank From Patients Affected by Genetic Diseases. Open Journal of Bioresources..

[50] Bolstad BM, Irizarry RA, Astrand M, Speed TP (2003). A comparison of normalization methods for high density oligonucleotide array data based on variance and bias. Bioinformatics..

[51] Krämer, A., Green, J., Pollard, J. Jr. & Tugendreich, S. Causal analysis approaches in Ingenuity Pathway Analysis. *Bioinformatics*. **30**(**4**), 523–30 (2014 Feb 15). 10.1093/bioinformatics/btt703 PMC3928520 24336805

