# Supplementary material for: Gene expression profile in patients with Gaucher disease indicates activation of inflammatory processes
Source: Sci Rep. 2019 Apr 15;9:6060. doi: 10.1038/s41598-019-42584-1 (PMC6465595; doi:10.1038/s41598-019-42584-1)
Supplement: Supplementary file 1 — Supplementary information [file 41598_2019_42584_MOESM1_ESM.pdf]

Supplementary information

Gene expression profile in patients with Gaucher disease indicates activation of inflammatory processes.

Agnieszka Ługowska[1*], Katarzyna Hetmańczyk-Sawicka[1], Roksana Iwanicka-Nowicka[2,3], Anna Fogtman[2], Jarosław Cieśla[4], Joanna Karolina Purzycka-Olewiecka[1], Dominika Sitarska[1], Rafał Płoski[5], Mirella Filocamo[6], Susanna Lualdi[6], Małgorzata Bednarska-Makaruk[1], Marta Koblowska[2,3]

[1] Department of Genetics, Institute of Psychiatry and Neurology, Warsaw, Poland

[2] Laboratory of Microarray Analysis, Institute of Biochemistry and Biophysics, Polish Academy of Sciences, Warsaw, Poland

[3] Laboratory of Systems Biology, Faculty of Biology, University of Warsaw, Warsaw, Poland

[4] Institute of Biochemistry and Biophysics, Polish Academy of Sciences, Warsaw, Poland

[5] Department of Medical Genetics, Warsaw Medical University, Warsaw, Poland.

[6] Laboratorio di Genetica Molecolare e Biobanche, Istituto G. Gaslini, L.go G. Gaslini -16147 Genova, Italy

*Corresponding author:

e-mail; alugipin@yahoo.com (AŁ)

Data were analysed and the networks were generated through the use of IPA (QIAGEN Inc., https://www.qiagenbioinformatics.com/products/ingenuity-pathway-analysis).

Supplementary Table S1. Genes with changed expression after microarray study. Illumina HumanHT-12 v4.0 Expression BeadChip arrays were used to identify gene expression profiles characteristic of GD patients and Control persons (results of the experiment were analyzed with IPA program).

Exp p-value	Exp Fold Change	ID	Entrez Gene Name	Location	Type(s)
0,013	3,675	SERPINB2	serpin family B member 2	Extracellular Space	other
0,004	3,242	PLAU	plasminogen activator, urokinase	Extracellular Space	peptidase
0,02	2,879	IL13RA2	interleukin 13 receptor subunit alpha 2	Plasma Membrane	transmembrane receptor
0,033	2,769	IFI6	interferon alpha inducible protein 6	Cytoplasm	other
0,01	2,605	TXNIP	thioredoxin interacting protein	Cytoplasm	other
0,003	2,568	IFIT2	interferon induced protein with tetratricopeptide repeats 2	Cytoplasm	other
0,021	2,555	IFIT1	interferon induced protein with tetratricopeptide repeats 1	Cytoplasm	other
0,01	2,473	NPTX1	neuronal pentraxin 1	Extracellular Space	other
0,046	2,457	TMEM158	transmembrane protein 158 (gene/pseudogene)	Plasma Membrane	other
0,005	2,362	GPR177	wntless Wnt ligand secretion mediator	Cytoplasm	other
0,007	2,326	LOC100129681			
0,012	2,317	DNER	delta/notch like EGF repeat containing	Plasma Membrane	transmembrane receptor
0,044	2,313	ISG15	ISG15 ubiquitin-like modifier	Extracellular Space	other
0,001	2,263	SPRY2	sprouty RTK signaling antagonist 2	Plasma Membrane	other
0,004	2,192	STAT1	signal transducer and activator of transcription 1	Nucleus	transcription regulator
0,04	2,128	CLDN11	claudin 11	Plasma Membrane	other
0,011	2,089	LOC642489			
0,048	2,083	UCHL1	ubiquitin C-terminal hydrolase L1	Cytoplasm	peptidase
0,023	2,073	HS.91389			
0,037	2,071	IFI44	interferon induced protein 44	Cytoplasm	other
0,02	2,061	HERC6	HECT and RLD domain containing E3 ubiquitin protein ligase family member 6	Cytoplasm	enzyme
0,018	2,058	IFI44L	interferon induced protein 44 like	Nucleus	other
0,026	2,017	ITGA2	integrin subunit alpha 2	Plasma Membrane	transmembrane receptor
0,01	2,01	PHLDA1	pleckstrin homology like domain family A member 1	Cytoplasm	other
0,002	1,998	FJX1	four jointed box 1	Extracellular Space	other
0,031	1,99	GNG11	G protein subunit gamma 11	Plasma Membrane	enzyme
0,003	1,988	DUSP6	dual specificity phosphatase 6	Cytoplasm	phosphatase
0,001	1,973	IGF2BP3	insulin like growth factor 2 mRNA binding protein 3	Cytoplasm	translation regulator
0,003	1,944	LY6E	lymphocyte antigen 6 complex, locus E	Plasma Membrane	other
0,027	1,93	OSR1	odd-skipped related transciption factor 1	Nucleus	other
0,005	1,905	BST2	bone marrow stromal cell antigen 2	Plasma Membrane	other
0,027	1,872	SLC20A1	solute carrier family 20 member 1	Plasma Membrane	transporter
0,007	1,872	DUSP5	dual specificity phosphatase 5	Nucleus	phosphatase
0,011	1,868	CAMK2N1	calcium/calmodulin dependent protein kinase II inhibitor 1	Plasma Membrane	kinase

0,021	1,853	RND3	Rho family GTPase 3	Cytoplasm	enzyme
0,014	1,832	TFPI	tissue factor pathway inhibitor	Extracellular Space	other
0,038	1,827	HERC5	HECT and RLD domain containing E3 ubiquitin protein ligase 5	Cytoplasm	enzyme
0,033	1,827	HPCAL1	hippocalcin like 1	Cytoplasm	other
0,017	1,815	OAS2	2'-5'-oligoadenylate synthetase 2	Cytoplasm	enzyme
0,023	1,806	SEMA3A	semaphorin 3A	Extracellular Space	other
0,008	1,798	IFIT3	interferon induced protein with tetratricopeptide repeats 3	Cytoplasm	other
0,007	1,798	MGST1	microsomal glutathione S-transferase 1	Cytoplasm	enzyme
0,006	1,796	SAMD9L	sterile alpha motif domain containing 9 like	Extracellular Space	other
0,036	1,793	NT5E	5'-nucleotidase ecto	Plasma Membrane	phosphatase
0,024	1,789	MIR1978			
0,004	1,788	LOC730278			
0,026	1,77	HMGA1	high mobility group AT-hook 1	Nucleus	transcription regulator
0,006	1,756	IFI16	interferon gamma inducible protein 16	Nucleus	transcription regulator
0,006	1,752	SAMD9	sterile alpha motif domain containing 9	Cytoplasm	other
0,046	1,737	TBX2	T-box 2	Nucleus	transcription regulator
0,01	1,734	CTSL1	cathepsin L	Cytoplasm	peptidase
0,016	1,725	TAP1	transporter 1, ATP-binding cassette, sub-family B (MDR/TAP)	Cytoplasm	transporter
0,04	1,723	C21ORF7			
0,007	1,702	SNHG8	small nucleolar RNA host gene 8	Other	other
0,035	1,701	RSAD2	radical S-adenosyl methionine domain containing 2	Cytoplasm	enzyme
0,041	1,681	FKBP1A	FK506 binding protein 1A	Cytoplasm	enzyme
0,049	1,663	FOXF1	forkhead box F1	Nucleus	transcription regulator
0,008	1,654	COL4A5	collagen type IV alpha 5	Extracellular Space	other
0,049	1,645	DDIT4L	DNA damage inducible transcript 4 like	Cytoplasm	other
0,036	1,644	CAV1	caveolin 1	Plasma Membrane	transmembrane receptor
0,015	1,632	MT1G	metallothionein 1G	Nucleus	other
0,029	1,626	IFIH1	interferon induced with helicase C domain 1	Nucleus	enzyme
0,004	1,62	LAP3	leucine aminopeptidase 3	Cytoplasm	peptidase
0,001	1,612	TPBG	trophoblast glycoprotein	Plasma Membrane	other
0,032	1,609	OAS3	2'-5'-oligoadenylate synthetase 3	Cytoplasm	enzyme
0,002	1,604	ANAPC13	anaphase promoting complex subunit 13	Nucleus	other
0,029	1,592	LPIN1	lipin 1	Nucleus	phosphatase
0,005	1,589	GLUD1	glutamate dehydrogenase 1	Cytoplasm	enzyme
0,043	1,583	AOX1	aldehyde oxidase 1	Cytoplasm	enzyme
0,015	1,582	M160			
0,027	1,576	TIPARP	TCDD inducible poly(ADP-ribose) polymerase	Nucleus	enzyme
0,022	1,567	CPNE3	copine 3	Cytoplasm	kinase
0,026	1,56	OAS1	2'-5'-oligoadenylate synthetase 1	Cytoplasm	enzyme

0,01	1,557	ABLIM3	actin binding LIM protein family member 3	Cytoplasm	other
0,049	1,557	RGS17	regulator of G-protein signaling 17	Cytoplasm	other
0,032	1,554	C20ORF108			
0,006	1,55	UCHL3	ubiquitin C-terminal hydrolase L3	Cytoplasm	peptidase
0,006	1,549	LY96	lymphocyte antigen 96	Plasma Membrane	transmembrane receptor
0,022	1,549	PERP	PERP, TP53 apoptosis effector	Plasma Membrane	other
0,003	1,545	CHMP5	charged multivesicular body protein 5	Cytoplasm	other
0,012	1,544	PRNP	prion protein	Plasma Membrane	other
0,017	1,538	KCNJ2	potassium voltage-gated channel subfamily J member 2	Plasma Membrane	ion channel
0,032	1,537	SLC7A14	solute carrier family 7 member 14	Cytoplasm	other
0,005	1,535	PRSS3	protease, serine 3	Extracellular Space	peptidase
0,008	1,531	OPTN	optineurin	Cytoplasm	other
0,008	1,528	HCP5	HLA complex P5 (non-protein coding)	Other	other
0,029	1,527	CASP1	caspase 1	Cytoplasm	peptidase
0,019	1,522	UBE2L6	ubiquitin conjugating enzyme E2 L6	Cytoplasm	enzyme
0,047	1,521	PARP9	poly(ADP-ribose) polymerase family member 9	Nucleus	enzyme
0,03	1,519	ARHGAP21	Rho GTPase activating protein 21	Cytoplasm	other
0,024	1,516	TMEM126B	transmembrane protein 126B	Cytoplasm	other
0,026	1,515	XAF1	XIAP associated factor 1	Nucleus	other
0,044	1,505	TXNDC17	thioredoxin domain containing 17	Cytoplasm	enzyme
0,006	1,497	RASSF2	Ras association domain family member 2	Nucleus	kinase
0,015	1,496	PSME1	proteasome activator subunit 1	Cytoplasm	other
0,025	1,494	KLF4	Kruppel-like factor 4 (gut)	Nucleus	transcription regulator
0,011	1,493	LOC650518			
0,001	1,491	ALDH9A1	aldehyde dehydrogenase 9 family member A1	Cytoplasm	enzyme
0,012	1,49	VPS29	VPS29 retromer complex component	Cytoplasm	transporter
0,019	1,489	ELTD1	adhesion G protein-coupled receptor L4	Plasma Membrane	G-protein coupled receptor
0,02	1,489	B2M	beta-2-microglobulin	Plasma Membrane	transmembrane receptor
0,021	1,488	IRF9	interferon regulatory factor 9	Nucleus	transcription regulator
0,05	1,486	LOC440043			
0,015	1,485	BCL2L1	BCL2 like 1	Cytoplasm	other
0,041	1,483	TMED10P	transmembrane p24 trafficking protein 10 pseudogene 1	Other	other
0,019	1,483	GK	glycerol kinase	Cytoplasm	kinase
0,04	1,481	C9ORF167			
0,014	1,478	ACAT1	acetyl-CoA acetyltransferase 1	Cytoplasm	enzyme
0,002	1,476	IMP3	IMP3, U3 small nucleolar ribonucleoprotein	Cytoplasm	other
0,036	1,475	LOC401397			
0,012	1,474	VPS35	VPS35 retromer complex component	Cytoplasm	transporter

0,023	1,471	BRI3P1	brain protein I3 pseudogene 1	Other	other
0,042	1,466	RGMB	repulsive guidance molecule family member b	Plasma Membrane	other
0,006	1,465	SLC35F5	solute carrier family 35 member F5	Other	other
0	1,456	UBA6	ubiquitin like modifier activating enzyme 6	Cytoplasm	enzyme
0,008	1,455	UBL5	ubiquitin like 5	Cytoplasm	other
0	1,454	LOC389599			
0,033	1,454	NARS	asparaginyl-tRNA synthetase	Cytoplasm	enzyme
0,002	1,454	CCNG1	cyclin G1	Nucleus	other
0,039	1,453	LOC88523			
0,01	1,452	IDH1	isocitrate dehydrogenase (NADP(+)) 1, cytosolic	Cytoplasm	enzyme
0,016	1,452	TRIM22	tripartite motif containing 22	Cytoplasm	transcription regulator
0,024	1,452	DLC1	DLC1 Rho GTPase activating protein	Cytoplasm	other
0,008	1,45	C7ORF30			
0,011	1,449	ELOVL6	ELOVL fatty acid elongase 6	Cytoplasm	enzyme
0,041	1,447	PBX3	pre-B-cell leukemia homeobox 3	Nucleus	transcription regulator
0,01	1,445	PRKG2	protein kinase, cGMP-dependent, type II	Cytoplasm	kinase
0,022	1,445	GLRX5	glutaredoxin 5	Cytoplasm	other
0,023	1,443	KCNMA1	potassium calcium-activated channel subfamily M alpha 1	Plasma Membrane	ion channel
0,009	1,442	SCO1	SCO1 cytochrome c oxidase assembly protein	Cytoplasm	other
0,024	1,44	PARP12	poly(ADP-ribose) polymerase family member 12	Nucleus	other
0,002	1,439	MRPL15	mitochondrial ribosomal protein L15	Cytoplasm	other
0,019	1,436	OASL	2'-5'-oligoadenylate synthetase like	Cytoplasm	enzyme
0,034	1,436	HIGD1A	HIG1 hypoxia inducible domain family member 1A	Cytoplasm	other
0	1,435	PDHX	pyruvate dehydrogenase complex component X	Cytoplasm	enzyme
0,019	1,434	CCDC72	translation machinery associated 7 homolog	Other	other
0,011	1,434	LOC729769			
0,003	1,431	TMX1	thioredoxin related transmembrane protein 1	Cytoplasm	enzyme
0,041	1,43	MAP1LC3B	microtubule associated protein 1 light chain 3 beta	Cytoplasm	other
0,032	1,426	XPO1	exportin 1	Nucleus	transporter
0,038	1,426	NDUFAB1	NADH:ubiquinone oxidoreductase subunit AB1	Cytoplasm	enzyme
0,01	1,426	SS18L2	SS18 like 2	Other	other
0,017	1,424	LARP6	La ribonucleoprotein domain family member 6	Other	other
0,012	1,424	MAP2K1IP1	late endosomal/lysosomal adaptor, MAPK and MTOR activator 3	Cytoplasm	other
0,033	1,422	PARP4	poly(ADP-ribose) polymerase family member 4	Cytoplasm	enzyme
0,019	1,42	MRPL50	mitochondrial ribosomal protein L50	Cytoplasm	other
0,006	1,419	TTC4	tetratricopeptide repeat domain 4	Other	other
0,035	1,419	IARS2	isoleucyl-tRNA synthetase 2, mitochondrial	Cytoplasm	enzyme
0,011	1,419	RTCD1	RNA 3'-terminal phosphate cyclase	Nucleus	enzyme

0,037	1,418	LGALS3	lectin, galactoside binding soluble 3	Extracellular Space	other
0,006	1,418	DNAJA3	DnaJ heat shock protein family (Hsp40) member A3	Cytoplasm	other
0,026	1,417	ETV5	ETS variant 5	Nucleus	transcription regulator
0,029	1,416	RHBDF1	rhomboid 5 homolog 1 (Drosophila)	Cytoplasm	other
0,044	1,415	PRSS12	protease, serine 12	Extracellular Space	peptidase
0	1,414	LYPLAL1	lysophospholipase like 1	Cytoplasm	enzyme
0,045	1,414	NAV3	neuron navigator 3	Nucleus	other
0,019	1,413	ADO	2-aminoethanethiol (cysteamine) dioxygenase	Cytoplasm	enzyme
0,034	1,411	CD47	CD47 molecule	Plasma Membrane	transmembrane receptor
0,048	1,411	LOC440063			
0,002	1,41	KIAA1826	Myb/SANT DNA binding domain containing 4 with coiled-coils	Nucleus	other
0,033	1,409	FAM98A	family with sequence similarity 98 member A	Other	other
0,001	1,408	C8ORF76			
0,045	1,408	NOL7	nucleolar protein 7	Nucleus	other
0,003	1,406	TXNDC9	thioredoxin domain containing 9	Cytoplasm	other
0,039	1,406	C20ORF30			
0,018	1,405	LOC401206			
0,036	1,405	ZMPSTE24	zinc metallopeptidase STE24	Nucleus	peptidase
0,002	1,405	UBLCP1	ubiquitin like domain containing CTD phosphatase 1	Nucleus	phosphatase
0,012	1,404	DNAJB4	DnaJ heat shock protein family (Hsp40) member B4	Nucleus	other
0,016	1,404	ERCC5	excision repair cross-complementation group 5	Nucleus	enzyme
0,045	1,403	PRDX3	peroxiredoxin 3	Cytoplasm	enzyme
0,04	1,403	RASGRP3	RAS guanyl releasing protein 3	Cytoplasm	other
0,029	-1,407	HNRPA1L-2			
0,026	-1,407	ZSWIM4	zinc finger SWIM-type containing 4	Other	other
0,004	-1,409	FOXF2	forkhead box F2	Nucleus	transcription regulator
0,044	-1,409	WNK4	WNK lysine deficient protein kinase 4	Plasma Membrane	kinase
0,003	-1,412	IER5L	immediate early response 5-like	Other	other
0,001	-1,415	LOC440311	glioma tumor suppressor candidate region gene 2 pseudogene	Other	other
0,006	-1,417	RAXL1	retina and anterior neural fold homeobox 2	Nucleus	transcription regulator
0	-1,421	NPLOC4	NPL4 homolog, ubiquitin recognition factor	Nucleus	other
0,005	-1,427	LMOD3	leiomodin 3	Other	other
0,022	-1,428	LOC729102			
0,049	-1,43	NT5DC2	5'-nucleotidase domain containing 2	Cytoplasm	other
0,004	-1,431	CCDC85B	coiled-coil domain containing 85B	Cytoplasm	other
0,014	-1,434	LOC729978			
0,002	-1,434	TMEM109	transmembrane protein 109	Cytoplasm	other
0,032	-1,435	DKFZP761P0423			
0,016	-1,439	RIPK4	receptor interacting serine/threonine kinase 4	Nucleus	kinase

0,026	-1,445	HSPBL2	heat shock protein family B (small) member 1 pseudogene 1	Other	other
0,023	-1,452	SSPN	sarcospan	Plasma Membrane	other
0,033	-1,454	LGMN	legumain	Cytoplasm	peptidase
0,02	-1,456	TNFAIP2	TNF alpha induced protein 2	Extracellular Space	other
0,019	-1,459	LOC100132247			
0,007	-1,463	DMPK	dystrophia myotonica protein kinase	Cytoplasm	kinase
0,001	-1,465	ARHGEF17	Rho guanine nucleotide exchange factor 17	Cytoplasm	other
0,004	-1,471	SPIN1	spindlin 1	Nucleus	other
0,022	-1,478	PFKFB3	6-phosphofructo-2-kinase/fructose-2,6-biphosphatase 3	Cytoplasm	kinase
0,011	-1,479	LOC613037	nuclear pore complex interacting protein member	Other	other
0,036	-1,488	ITM2C	integral membrane protein 2C	Cytoplasm	other
0,018	-1,489	GOLGA8A	golgin A8 family member A	Cytoplasm	other
0,015	-1,489	CYTH2	cytohesin 2	Cytoplasm	other
0,023	-1,49	SIX5	SIX homeobox 5	Nucleus	transcription regulator
0,043	-1,496	LOC643287			
0,023	-1,496	ALDOC	aldolase, fructose-bisphosphate C	Cytoplasm	enzyme
0,013	-1,5	LOC642412			
0,041	-1,5	MAP3K8	mitogen-activated protein kinase kinase kinase 8	Cytoplasm	kinase
0,04	-1,508	FOXC1	forkhead box C1	Nucleus	transcription regulator
0,003	-1,511	KLF13	Kruppel-like factor 13	Nucleus	transcription regulator
0,015	-1,513	LOC727882			
0,032	-1,517	LATS2	large tumor suppressor kinase 2	Nucleus	kinase
0,002	-1,518	MXRA8	matrix-remodelling associated 8	Cytoplasm	other
0,035	-1,52	CLCF1	cardiotrophin-like cytokine factor 1	Extracellular Space	cytokine
0,01	-1,523	IDH2	isocitrate dehydrogenase (NADP(+)) 2, mitochondrial	Cytoplasm	enzyme
0,027	-1,524	SPHK1	sphingosine kinase 1	Cytoplasm	kinase
0,014	-1,536	GPC1	glypican 1	Plasma Membrane	transmembrane receptor
0,001	-1,541	MGC16384			
0,015	-1,549	SGCA	sarcoglycan alpha	Plasma Membrane	other
0,016	-1,552	YPEL3	yippee like 3	Other	other
0,016	-1,563	CRLF1	cytokine receptor like factor 1	Extracellular Space	other
0,02	-1,572	C6ORF145			
0,005	-1,604	B4GALT1	beta-1,4-galactosyltransferase 1	Cytoplasm	enzyme
0,015	-1,614	SLC39A14	solute carrier family 39 member 14	Plasma Membrane	transporter
0	-1,615	THOC6	THO complex 6	Nucleus	other
0,018	-1,655	LOC728873			
0,016	-1,681	HS.374257	ST3 beta-galactoside alpha-2,3-sialyltransferase 1	Cytoplasm	enzyme
0,008	-1,691	CRIPAK	cysteine rich PAK1 inhibitor	Other	other
0,033	-1,72	S1PR3	sphingosine-1-phosphate receptor 3	Plasma Membrane	G-protein coupled

					receptor
0,038	-1,728	CILP	cartilage intermediate layer protein	Extracellular Space	phosphatase
0,029	-1,729	GAPDHL6	glyceraldehyde 3 phosphate dehydrogenase pseudogene 61	Other	other
0,019	-1,731	FSTL3	follistatin like 3	Extracellular Space	other
0,022	-1,75	SERPINH1	serpin family H member 1	Extracellular Space	other
0,036	-1,754	NPTX2	neuronal pentraxin 2	Extracellular Space	other
0,001	-1,765	PTK7	protein tyrosine kinase 7 (inactive)	Plasma Membrane	kinase
0,022	-1,77	C1ORF198			
0,013	-1,778	SMAD9	SMAD family member 9	Nucleus	transcription regulator
0	-1,792	SAMD11	sterile alpha motif domain containing 11	Nucleus	other
0,028	-1,807	LITAF	lipopolysaccharide induced TNF factor	Nucleus	transcription regulator
0,03	-1,808	CD24	CD24 molecule	Plasma Membrane	other
0,036	-1,826	TSHZ2	teashirt zinc finger homeobox 2	Other	other
0,031	-1,827	CSRP2	cysteine and glycine rich protein 2	Nucleus	other
0,002	-1,866	ZNF503	zinc finger protein 503	Nucleus	other
0,007	-1,885	EIF5A	eukaryotic translation initiation factor 5A	Cytoplasm	translation regulator
0,002	-1,921	RASD2	RASD family member 2	Cytoplasm	enzyme
0,014	-1,949	HEY1	hes related family bHLH transcription factor with YRPW motif 1	Nucleus	transcription regulator
0,014	-1,961	ID3	inhibitor of DNA binding 3, HLH protein	Nucleus	transcription regulator
0,017	-1,988	MYLIP	myosin regulatory light chain interacting protein	Cytoplasm	enzyme
0,029	-1,998	CSRP1	cysteine and glycine rich protein 1	Nucleus	other
0,008	-2,023	C1QTNF5	C1q and tumor necrosis factor related protein 5	Plasma Membrane	transmembrane receptor
0,001	-2,07	ISLR	immunoglobulin superfamily containing leucine-rich repeat	Extracellular Space	other
0,023	-2,103	CNN1	calponin 1	Cytoplasm	other
0,01	-2,323	NNMT	nicotinamide N-methyltransferase	Cytoplasm	enzyme
0,002	-2,354	ATOH8	atonal bHLH transcription factor 8	Nucleus	transcription regulator
0,008	-2,445	THBS2	thrombospondin 2	Extracellular Space	other
0,039	-2,689	CRISPLD2	cysteine rich secretory protein LCCL domain containing 2	Cytoplasm	other
0,029	-4,027	IGFBP5	insulin like growth factor binding protein 5	Extracellular Space	other

Supplementary Table S2. Genes with changed expression after microarray study (analysis GD vs. NPC). Illumina HumanHT-12 v4.0 Expression BeadChip arrays were used to identify gene expression profiles characteristic of GD patients and NPC patients (results of the experiment were analyzed with IPA program).

Exp p-value	Exp Fold Change	ID	Entrez Gene Name	Location	Type(s)
0,045	3,139	MX1	MX dynamin like GTPase 1	Cytoplasm	enzyme
0,023	2,786	TMEM158	transmembrane protein 158 (gene/pseudogene)	Plasma Membrane	other
0,008	2,727	UCHL1	ubiquitin C-terminal hydrolase L1	Cytoplasm	peptidase
0,02	2,637	ISG15	ISG15 ubiquitin-like modifier	Extracellular Space	other
0,018	2,591	PLAU	plasminogen activator, urokinase	Extracellular Space	peptidase
0,024	2,508	FOS	FBJ murine osteosarcoma viral oncogene homolog	Nucleus	transcription regulator
0,012	2,473	MLLT11	myeloid/lymphoid or mixed-lineage leukemia; translocated to, 11	Cytoplasm	other
0,03	2,384	IFIT1	interferon induced protein with tetratricopeptide repeats 1	Cytoplasm	other
0,004	2,38	PLOD2	procollagen-lysine,2-oxoglutarate 5-dioxygenase 2	Cytoplasm	enzyme
0,006	2,339	LOC100129681			
0	2,256	IGF2BP3	insulin like growth factor 2 mRNA binding protein 3	Cytoplasm	translation regulator
0,001	2,175	DUSP6	dual specificity phosphatase 6	Cytoplasm	phosphatase
0,004	2,162	STAT1	signal transducer and activator of transcription 1	Nucleus	transcription regulator
0,015	2,085	IFI44L	interferon induced protein 44 like	Nucleus	other
0,018	2,084	IFIT2	interferon induced protein with tetratricopeptide repeats 2	Cytoplasm	other
0,011	2,055	SLC20A1	solute carrier family 20 member 1	Plasma Membrane	transporter
0,017	2,051	GPR177	wntless Wnt ligand secretion mediator	Cytoplasm	other
0,003	1,991	DUSP5	dual specificity phosphatase 5	Nucleus	phosphatase
0,038	1,973	DNER	delta/notch like EGF repeat containing	Plasma Membrane	transmembrane receptor
0,021	1,946	C8ORF13			
0,018	1,945	FAM167A	family with sequence similarity 167 member A	Other	other
0,022	1,941	WNT5A	Wnt family member 5A	Extracellular Space	cytokine
0,013	1,913	FOXF1	forkhead box F1	Nucleus	transcription regulator
0	1,898	TPBG	trophoblast glycoprotein	Plasma Membrane	other
0,043	1,895	HS.91389			
0,001	1,886	COL4A5	collagen type IV alpha 5	Extracellular Space	other
0,021	1,883	RGS2	regulator of G-protein signaling 2	Nucleus	other

0,018	1,876	RND3	Rho family GTPase 3	Cytoplasm	enzyme
0,006	1,857	BST2	bone marrow stromal cell antigen 2	Plasma Membrane	other
0,013	1,854	DOCK10	dedicator of cytokinesis 10	Cytoplasm	other
0,025	1,851	WARS	tryptophanyl-tRNA synthetase	Cytoplasm	enzyme
0,045	1,849	HERC6	HECT and RLD domain containing E3 ubiquitin protein ligase family member 6	Cytoplasm	enzyme
0,011	1,828	SPRY2	sprouty RTK signaling antagonist 2	Plasma Membrane	other
0,036	1,824	AKR1B1	aldo-keto reductase family 1, member B1 (aldose reductase)	Cytoplasm	enzyme
0,02	1,815	LMO4	LIM domain only 4	Nucleus	transcription regulator
0,007	1,809	FJX1	four jointed box 1	Extracellular Space	other
0,032	1,805	TFAP2C	transcription factor AP-2 gamma	Nucleus	transcription regulator
0,04	1,805	C20ORF100			
0,003	1,803	SNHG8	small nucleolar RNA host gene 8	Other	other
0,032	1,799	VEGFC	vascular endothelial growth factor C	Extracellular Space	growth factor
0,039	1,789	ASS1	argininosuccinate synthase 1	Cytoplasm	enzyme
0,023	1,787	GARS	glycyl-tRNA synthetase	Cytoplasm	enzyme
0,049	1,77	HS.444692			
0,03	1,769	SH2B3	SH2B adaptor protein 3	Plasma Membrane	other
0,013	1,761	NXN	nucleoredoxin	Nucleus	enzyme
0,015	1,754	ADAM19	ADAM metallopeptidase domain 19	Plasma Membrane	peptidase
0,013	1,752	GLRX	glutaredoxin	Cytoplasm	enzyme
0,032	1,748	SLC2A1	solute carrier family 2 member 1	Plasma Membrane	transporter
0,014	1,732	HS.193406	family with sequence similarity 212 member B	Other	other
0,027	1,729	BNIP3	BCL2/adenovirus E1B 19kDa interacting protein 3	Cytoplasm	other
0,015	1,725	OLR1	oxidized low density lipoprotein receptor 1	Plasma Membrane	transmembrane receptor
0,001	1,716	PELO	pelota homolog (Drosophila)	Nucleus	other
0,03	1,712	OAS2	2'-5'-oligoadenylate synthetase 2	Cytoplasm	enzyme
0,014	1,711	CTPS	CTP synthase 1	Nucleus	enzyme
0,046	1,708	CITED2	Cbp/p300 interacting transactivator with Glu/Asp rich carboxy-terminal domain 2	Nucleus	transcription regulator
0,006	1,7	PERP	PERP, TP53 apoptosis effector	Plasma Membrane	other
0,007	1,699	CHN1	chimerin 1	Cytoplasm	other
0,006	1,699	TFRC	transferrin receptor	Plasma Membrane	transporter
0,01	1,696	LOC440043			
0,014	1,694	LY6E	lymphocyte antigen 6 complex, locus E	Plasma Membrane	other
0,007	1,692	PFKFB4	6-phosphofructo-2-kinase/fructose-2,6-biphosphatase 4	Cytoplasm	kinase
0,04	1,69	SEMA3A	semaphorin 3A	Extracellular Space	other

0,037	1,683	RSAD2	radical S-adenosyl methionine domain containing 2	Cytoplasm	enzyme
0,003	1,681	NAV3	neuron navigator 3	Nucleus	other
0,05	1,678	MTHFD2	methylenetetrahydrofolate dehydrogenase (NADP+ dependent) 2, methenyltetrahydrofolate cyclohydrolase	Cytoplasm	enzyme
0,005	1,67	TARS	threonyl-tRNA synthetase	Nucleus	enzyme
0,019	1,665	IFIT3	interferon induced protein with tetratricopeptide repeats 3	Cytoplasm	other
0,037	1,656	LOC644774			
0,018	1,655	MGST1	microsomal glutathione S-transferase 1	Cytoplasm	enzyme
0,004	1,654	PRNP	prion protein	Plasma Membrane	other
0,04	1,651	CAV2	caveolin 2	Plasma Membrane	other
0,017	1,65	TPI1	triosephosphate isomerase 1	Cytoplasm	enzyme
0,009	1,649	C9ORF167			
0,033	1,648	SLC16A3	solute carrier family 16 member 3	Plasma Membrane	transporter
0,016	1,647	GPT2	glutamic pyruvate transaminase (alanine aminotransferase) 2	Cytoplasm	enzyme
0,008	1,64	DDX10	DEAD-box helicase 10	Nucleus	enzyme
0,042	1,638	RHOB	ras homolog family member B	Cytoplasm	enzyme
0,004	1,629	GK	glycerol kinase	Cytoplasm	kinase
0,007	1,627	IARS	isoleucyl-tRNA synthetase	Cytoplasm	enzyme
0,017	1,615	PFN2	profilin 2	Cytoplasm	other
0,014	1,607	DCBLD1	discoidin, CUB and LCCL domain containing 1	Extracellular Space	other
0,017	1,606	SAMD9	sterile alpha motif domain containing 9	Cytoplasm	other
0,006	1,605	GBP1	guanylate binding protein 1	Cytoplasm	enzyme
0,003	1,604	LOC650518			
0,02	1,601	IFI16	interferon gamma inducible protein 16	Nucleus	transcription regulator
0,003	1,599	UCHL3	ubiquitin C-terminal hydrolase L3	Cytoplasm	peptidase
0,011	1,591	PSMA4	proteasome subunit alpha 4	Cytoplasm	peptidase
0,005	1,584	SEC11C	SEC11 homolog C, signal peptidase complex subunit	Cytoplasm	peptidase
0,022	1,577	OAS1	2'-5'-oligoadenylate synthetase 1	Cytoplasm	enzyme
0,004	1,575	HCP5	HLA complex P5 (non-protein coding)	Other	other
0,017	1,574	P4HA1	prolyl 4-hydroxylase subunit alpha 1	Cytoplasm	enzyme
0,023	1,571	LOC730278			
0,024	1,568	LOC732007			
0	1,566	UBA6	ubiquitin like modifier activating enzyme 6	Cytoplasm	enzyme
0	1,561	PDHX	pyruvate dehydrogenase complex component X	Cytoplasm	enzyme

0,001	1,559	LARS	leucyl-tRNA synthetase	Cytoplasm	enzyme
0,047	1,557	TAP1	transporter 1, ATP-binding cassette, sub-family B (MDR/TAP)	Cytoplasm	transporter
0,008	1,555	DLC1	DLC1 Rho GTPase activating protein	Cytoplasm	other
0,005	1,555	PAICS	phosphoribosylaminoimidazole carboxylase; phosphoribosylaminoimidazolesuccinocarboxamide synthase	Cytoplasm	enzyme
0,032	1,551	SAMD9L	sterile alpha motif domain containing 9 like	Extracellular Space	other
0,026	1,55	ADAMTS6	ADAM metallopeptidase with thrombospondin type 1 motif 6	Extracellular Space	peptidase
0,006	1,55	CCT2	chaperonin containing TCP1 subunit 2	Cytoplasm	kinase
0	1,549	BRIX1	BRX1, biogenesis of ribosomes	Nucleus	other
0,011	1,546	NDUFAB1	NADH:ubiquinone oxidoreductase subunit AB1	Cytoplasm	enzyme
0,023	1,546	PRDX6	peroxiredoxin 6	Cytoplasm	enzyme
0,028	1,544	MT1G	metallothionein 1G	Nucleus	other
0,008	1,543	LAP3	leucine aminopeptidase 3	Cytoplasm	peptidase
0,048	1,543	OAS3	2'-5'-oligoadenylate synthetase 3	Cytoplasm	enzyme
0,019	1,542	RAB32	RAB32, member RAS oncogene family	Cytoplasm	enzyme
0,011	1,542	PRDX3	peroxiredoxin 3	Cytoplasm	enzyme
0,001	1,539	IMP3	IMP3, U3 small nucleolar ribonucleoprotein	Cytoplasm	other
0	1,538	EEF1E1	eukaryotic translation elongation factor 1 epsilon 1	Cytoplasm	translation regulator
0	1,534	STRADB	STE20-related kinase adaptor beta	Cytoplasm	kinase
0,025	1,534	CKAP4	cytoskeleton-associated protein 4	Cytoplasm	other
0,012	1,533	EBNA1BP2	EBNA1 binding protein 2	Nucleus	other
0,015	1,533	NARS	asparaginyl-tRNA synthetase	Cytoplasm	enzyme
0,002	1,531	SCO1	SCO1 cytochrome c oxidase assembly protein	Cytoplasm	other
0,021	1,531	HSPA9	heat shock protein family A (Hsp70) member 9	Cytoplasm	other
0,004	1,529	ADO	2-aminoethanethiol (cysteamine) dioxygenase	Cytoplasm	enzyme
0	1,529	LOC389599			
0,004	1,529	CHMP5	charged multivesicular body protein 5	Cytoplasm	other
0,007	1,527	VPS29	VPS29 retromer complex component	Cytoplasm	transporter
0,028	1,525	SYTL2	synaptotagmin like 2	Cytoplasm	other
0,01	1,524	ATP5C1	ATP synthase, H+ transporting, mitochondrial F1 complex, gamma polypeptide 1	Cytoplasm	transporter
0,024	1,522	C14ORF156			
0,016	1,522	PTS	6-pyruvoyltetrahydropterin synthase	Cytoplasm	enzyme
0,013	1,522	KPNB1	karyopherin subunit beta 1	Nucleus	transporter
0,013	1,521	PSMD14	proteasome 26S subunit, non-ATPase 14	Cytoplasm	peptidase
0,03	1,519	ODC1	ornithine decarboxylase 1	Cytoplasm	enzyme

0,017	1,519	SRXN1	sulfiredoxin 1	Cytoplasm	enzyme
0,042	1,516	INHBE	inhibin beta E	Extracellular Space	growth factor
0,012	1,514	MRPL17	mitochondrial ribosomal protein L17	Cytoplasm	other
0	1,513	CLDN1	claudin 1	Plasma Membrane	other
0,005	1,513	SPATS2L	spermatogenesis associated serine rich 2 like	Nucleus	other
0,003	1,51	PSMA6	proteasome subunit alpha 6	Cytoplasm	peptidase
0,016	1,507	HIGD1A	HIG1 hypoxia inducible domain family member 1A	Cytoplasm	other
0,044	1,506	NME1	NME/NM23 nucleoside diphosphate kinase 1	Cytoplasm	kinase
0,014	1,505	KPNA4	karyopherin subunit alpha 4	Nucleus	transporter
0,006	1,504	C1QBP	complement component 1, q subcomponent binding protein	Cytoplasm	transcription regulator
0,035	1,504	CPNE3	copine 3	Cytoplasm	kinase
0,007	1,504	PRSS3	protease, serine 3	Extracellular Space	peptidase
0,008	1,503	ME1	malic enzyme 1	Cytoplasm	enzyme
0,006	1,502	C2ORF25			
0,035	1,502	LOC100128266			
0,001	1,501	LOC100130308			
0,019	1,5	MORF4L2	mortality factor 4 like 2	Nucleus	other
0,003	1,498	DCUN1D5	defective in cullin neddylation 1 domain containing 5	Other	other
0,035	1,498	MARS	methionyl-tRNA synthetase	Cytoplasm	enzyme
0,001	1,498	DNAJA3	DnaJ heat shock protein family (Hsp40) member A3	Cytoplasm	other
0,007	1,497	TST	thiosulfate sulfurtransferase	Cytoplasm	enzyme
0,019	1,496	MYC	v-myc avian myelocytomatosis viral oncogene homolog	Nucleus	transcription regulator
0,001	1,494	RPAIN	RPA interacting protein	Nucleus	other
0,011	1,492	TMEM171	transmembrane protein 171	Other	other
0,032	1,491	M160			
0,037	1,49	TMED10P	transmembrane p24 trafficking protein 10 pseudogene 1	Other	other
0,012	1,488	OXSR1	oxidative stress responsive 1	Nucleus	kinase
0,001	1,487	MRPL15	mitochondrial ribosomal protein L15	Cytoplasm	other
0,035	1,487	LDHA	lactate dehydrogenase A	Cytoplasm	enzyme
0,008	1,487	ANAPC13	anaphase promoting complex subunit 13	Nucleus	other
0,009	1,484	LDHB	lactate dehydrogenase B	Cytoplasm	enzyme
0,002	1,481	UBE2E1	ubiquitin conjugating enzyme E2 E1	Cytoplasm	enzyme
0,042	1,48	ACTR3	ARP3 actin-related protein 3 homolog (yeast)	Plasma Membrane	other
0,027	1,48	OSTC	oligosaccharyltransferase complex subunit	Cytoplasm	enzyme

			(non-catalytic)		
0,033	1,48	PGAM1	phosphoglycerate mutase 1	Cytoplasm	phosphatase
0,018	1,478	KLF6	Kruppel-like factor 6	Nucleus	transcription regulator
0,003	1,477	PMAIP1	phorbol-12-myristate-13-acetate-induced protein 1	Cytoplasm	other
0,022	1,476	NOL7	nucleolar protein 7	Nucleus	other
0,039	1,475	GFPT1	glutamine--fructose-6-phosphate transaminase 1	Cytoplasm	enzyme
0,003	1,475	PPA1	pyrophosphatase (inorganic) 1	Cytoplasm	enzyme
0,005	1,473	AHCY	adenosylhomocysteinase	Cytoplasm	enzyme
0,046	1,473	HPRT1	hypoxanthine phosphoribosyltransferase 1	Cytoplasm	enzyme
0,037	1,472	XAF1	XIAP associated factor 1	Nucleus	other
0,011	1,471	POLR2H	polymerase (RNA) II subunit H	Nucleus	enzyme
0,04	1,466	MGC16121			
0,018	1,465	HDDC2	HD domain containing 2	Cytoplasm	other
0,001	1,461	ZZZ3	zinc finger ZZ-type containing 3	Nucleus	other
0,004	1,459	RAP1GDS1	Rap1 GTPase-GDP dissociation stimulator 1	Cytoplasm	other
0,006	1,459	C7ORF30			
0,046	1,458	LOC645638			
0,005	1,458	DNAJB4	DnaJ heat shock protein family (Hsp40) member B4	Nucleus	other
0,003	1,457	TTC4	tetratricopeptide repeat domain 4	Other	other
0,023	1,456	STRAP	serine/threonine kinase receptor associated protein	Plasma Membrane	other
0,039	1,456	HSP90B1	heat shock protein 90kDa beta family member 1	Cytoplasm	other
0,044	1,455	YARS	tyrosyl-tRNA synthetase	Cytoplasm	enzyme
0,016	1,455	ETV5	ETS variant 5	Nucleus	transcription regulator
0,001	1,454	RDH14	retinol dehydrogenase 14 (all-trans/9-cis/11-cis)	Cytoplasm	enzyme
0,036	1,452	CDCP1	CUB domain containing protein 1	Plasma Membrane	other
0,022	1,452	NCKAP1	NCK associated protein 1	Plasma Membrane	other
0,014	1,451	VBP1	von Hippel-Lindau binding protein 1	Cytoplasm	other
0,016	1,451	MYPN	myopalladin	Cytoplasm	other
0,009	1,451	CCT8	chaperonin containing TCP1 subunit 8	Cytoplasm	enzyme
0,001	1,45	PRMT3	protein arginine methyltransferase 3	Nucleus	enzyme
0,029	1,449	PSME2	proteasome activator subunit 2	Cytoplasm	peptidase
0,001	1,448	RBCK1	RANBP2-type and C3HC4-type zinc finger containing 1	Cytoplasm	transcription regulator
0,035	1,447	GTF3A	general transcription factor IIIA	Nucleus	transcription regulator

0,001	1,446	CCDC50	coiled-coil domain containing 50	Cytoplasm	other
0	1,446	C8ORF76			
0,001	1,444	LOC647150			
0,048	1,444	PGM1	phosphoglucomutase 1	Cytoplasm	enzyme
0,014	1,441	MRPL50	mitochondrial ribosomal protein L50	Cytoplasm	other
0,017	1,44	EPRS	glutamyl-prolyl-tRNA synthetase	Cytoplasm	enzyme
0,019	1,439	LOC389787			
0,001	1,438	TXNDC9	thioredoxin domain containing 9	Cytoplasm	other
0,027	1,438	TIMM10	translocase of inner mitochondrial membrane 10 homolog (yeast)	Cytoplasm	transporter
0,012	1,437	HLA-C	major histocompatibility complex, class I, C	Plasma Membrane	other
0,036	1,436	CDR2	cerebellar degeneration related protein 2	Cytoplasm	other
0,016	1,433	ETFA	electron transfer flavoprotein alpha subunit	Cytoplasm	transporter
0,02	1,433	GSPT1	G1 to S phase transition 1	Cytoplasm	translation regulator
0,001	1,432	HS.71947			
0,011	1,431	POLE3	polymerase (DNA) epsilon 3, accessory subunit	Nucleus	enzyme
0,003	1,431	STK39	serine/threonine kinase 39	Nucleus	kinase
0,033	1,43	NDUFA8	NADH:ubiquinone oxidoreductase subunit A8	Cytoplasm	enzyme
0,006	1,429	DDX47	DEAD-box helicase 47	Nucleus	enzyme
0,034	1,428	TCF21	transcription factor 21	Nucleus	transcription regulator
0,031	1,427	IARS2	isoleucyl-tRNA synthetase 2, mitochondrial	Cytoplasm	enzyme
0,003	1,427	EIF2B2	eukaryotic translation initiation factor 2B subunit beta	Cytoplasm	other
0,001	1,426	GTF2H2B	general transcription factor IIH subunit 2B (pseudogene)	Other	other
0,028	1,426	ZMPSTE24	zinc metallopeptidase STE24	Nucleus	peptidase
0,018	1,426	EPS8	epidermal growth factor receptor pathway substrate 8	Plasma Membrane	peptidase
0,003	1,425	ABCE1	ATP binding cassette subfamily E member 1	Cytoplasm	transporter
0,021	1,424	OASL	2'-5'-oligoadenylate synthetase like	Cytoplasm	enzyme
0,004	1,423	BCL2L13	BCL2 like 13	Cytoplasm	other
0,028	1,422	C20ORF24			
0,038	1,421	TMEM2	transmembrane protein 2	Cytoplasm	other
0,028	1,42	GLRX5	glutaredoxin 5	Cytoplasm	other
0,009	1,42	GHITM	growth hormone inducible transmembrane protein	Cytoplasm	other
0,002	1,42	KIAA1826	Myb/SANT DNA binding domain containing 4 with coiled-coils	Nucleus	other
0,013	1,419	LOC729769			

0,002	1,419	RPF2	ribosome production factor 2 homolog	Nucleus	other
0	1,418	NOC3L	NOC3 like DNA replication regulator	Nucleus	other
0,025	1,417	HINT1	histidine triad nucleotide binding protein 1	Nucleus	enzyme
0,005	1,416	SACM1L	SAC1 suppressor of actin mutations 1-like (yeast)	Cytoplasm	phosphatase
0,008	1,416	AK2	adenylate kinase 2	Cytoplasm	kinase
0,003	1,415	USO1	USO1 vesicle transport factor	Cytoplasm	transporter
0,002	1,413	NDUFAF2	NADH:ubiquinone oxidoreductase complex assembly factor 2	Cytoplasm	enzyme
0,038	1,412	MAPRE1	microtubule associated protein RP/EB family member 1	Cytoplasm	other
0,046	1,412	LOC440063			
0,014	1,412	PPP4R1	protein phosphatase 4 regulatory subunit 1	Other	phosphatase
0,006	1,411	MRPL14	mitochondrial ribosomal protein L14	Cytoplasm	other
0,017	1,411	BZW2	basic leucine zipper and W2 domains 2	Cytoplasm	translation regulator
0,037	1,41	XPO1	exportin 1	Nucleus	transporter
0,034	1,41	PSMC2	proteasome 26S subunit, ATPase 2	Nucleus	peptidase
0,014	1,409	SCYL2	SCY1 like pseudokinase 2	Cytoplasm	other
0,032	1,409	MRPL36	mitochondrial ribosomal protein L36	Cytoplasm	other
0,017	1,408	TMED3	transmembrane p24 trafficking protein 3	Cytoplasm	transporter
0,009	1,407	DUSP10	dual specificity phosphatase 10	Nucleus	phosphatase
0,032	1,406	PARP12	poly(ADP-ribose) polymerase family member 12	Nucleus	other
0,008	1,405	PSMG1	proteasome (prosome, macropain) assembly chaperone 1	Plasma Membrane	other
0,005	1,405	PRPF4	pre-mRNA processing factor 4	Nucleus	other
0,022	1,405	UQCRHL	ubiquinol-cytochrome c reductase hinge protein like	Cytoplasm	other
0,001	1,404	C20ORF45			
0,026	1,404	CTNNAL1	catenin alpha like 1	Plasma Membrane	other
0,028	1,402	FAM129B	family with sequence similarity 129 member B	Cytoplasm	other
0,002	1,402	ZC3H15	zinc finger CCCH-type containing 15	Nucleus	other
0,002	1,401	STK4	serine/threonine kinase 4	Cytoplasm	kinase
0,02	1,401	HS.413494			
0,043	-1,403	NFKB1	nuclear factor kappa B subunit 1	Nucleus	transcription regulator
0,006	-1,407	THOC6	THO complex 6	Nucleus	other
0,047	-1,41	VWCE	von Willebrand factor C and EGF domains	Other	other
0,034	-1,414	DACT3	dishevelled-binding antagonist of beta-catenin 3	Cytoplasm	other
0,015	-1,414	KLHL21	kelch like family member 21	Cytoplasm	other

0,018	-1,416	C7ORF41			
0,021	-1,42	UAP1L1	UDP-N-acetylglucosamine pyrophosphorylase 1 like 1	Other	other
0,036	-1,422	LOC727882			
0,032	-1,426	B4GALT1	beta-1,4-galactosyltransferase 1	Cytoplasm	enzyme
0,037	-1,443	MMP2	matrix metallopeptidase 2	Extracellular Space	peptidase
0,019	-1,448	BTG2	BTG family member 2	Nucleus	transcription regulator
0,006	-1,455	MXRA8	matrix-remodelling associated 8	Cytoplasm	other
0	-1,456	CCDC74A	coiled-coil domain containing 74A	Other	other
0,017	-1,46	FAM21A	family with sequence similarity 21 member C	Cytoplasm	other
0,043	-1,466	APPL2	adaptor protein, phosphotyrosine interacting with PH domain and leucine zipper 2	Cytoplasm	other
0,016	-1,477	LOC642412			
0,023	-1,485	SIX5	SIX homeobox 5	Nucleus	transcription regulator
0,04	-1,49	SMAD7	SMAD family member 7	Nucleus	transcription regulator
0,001	-1,501	CCDC85B	coiled-coil domain containing 85B	Cytoplasm	other
0,004	-1,507	ZCCHC14	zinc finger CCHC-type containing 14	Other	other
0,006	-1,508	RIPK4	receptor interacting serine/threonine kinase 4	Nucleus	kinase
0,034	-1,509	CXXC5	CXXC finger protein 5	Nucleus	other
0,001	-1,516	MGC16384			
0,03	-1,537	MAP3K8	mitogen-activated protein kinase kinase kinase 8	Cytoplasm	kinase
0,016	-1,545	YPEL3	yippee like 3	Other	other
0,01	-1,553	TWIST1	twist family bHLH transcription factor 1	Nucleus	transcription regulator
0	-1,56	SLC7A8	solute carrier family 7 member 8	Plasma Membrane	transporter
0,006	-1,562	TNFAIP2	TNF alpha induced protein 2	Extracellular Space	other
0,027	-1,569	RAB3IL1	RAB3A interacting protein like 1	Other	other
0,002	-1,606	A4GALT	alpha 1,4-galactosyltransferase	Cytoplasm	enzyme
0,001	-1,607	KLF13	Kruppel-like factor 13	Nucleus	transcription regulator
0,012	-1,613	PRRX2	paired related homeobox 2	Nucleus	transcription regulator
0,005	-1,619	CTSA	cathepsin A	Cytoplasm	peptidase
0,006	-1,632	SGCA	sarcoglycan alpha	Plasma Membrane	other
0,005	-1,647	PLEKHF1	pleckstrin homology and FYVE domain containing 1	Cytoplasm	other
0,022	-1,664	KIAA1644	KIAA1644	Other	other
0,015	-1,671	PROS1	protein S (alpha)	Extracellular Space	other
0,028	-1,671	OLFML3	olfactomedin like 3	Extracellular Space	other
0	-1,699	FOXF2	forkhead box F2	Nucleus	transcription regulator
0,004	-1,7	CRLF1	cytokine receptor like factor 1	Extracellular Space	other

0,001	-1,743	SAMD11	sterile alpha motif domain containing 11	Nucleus	other
0,001	-1,757	C16ORF45			
0,031	-1,778	LITAF	lipopolysaccharide induced TNF factor	Nucleus	transcription regulator
0,003	-1,832	RASD2	RASD family member 2	Cytoplasm	enzyme
0,008	-1,85	EIF5A	eukaryotic translation initiation factor 5A	Cytoplasm	translation regulator
0,013	-1,867	SMAD6	SMAD family member 6	Nucleus	transcription regulator
0,013	-1,908	C1QTNF5	C1q and tumor necrosis factor related protein 5	Plasma Membrane	transmembrane receptor
0,006	-1,911	SCARA3	scavenger receptor class A member 3	Plasma Membrane	transmembrane receptor
0,042	-1,913	OLFML2B	olfactomedin like 2B	Extracellular Space	other
0,016	-1,921	ID3	inhibitor of DNA binding 3, HLH protein	Nucleus	transcription regulator
0,011	-1,953	MAFB	v-maf avian musculoaponeurotic fibrosarcoma oncogene homolog B	Nucleus	transcription regulator
0,035	-1,971	ID1	inhibitor of DNA binding 1, HLH protein	Nucleus	transcription regulator
0,011	-1,986	HEY1	hes related family bHLH transcription factor with YRPW motif 1	Nucleus	transcription regulator
0,024	-2,097	ANGPTL2	angiopoietin like 2	Extracellular Space	other
0	-2,128	ZNF503	zinc finger protein 503	Nucleus	other
0,001	-2,138	SMAD9	SMAD family member 9	Nucleus	transcription regulator
0,027	-2,216	FBLN2	fibulin 2	Extracellular Space	other
0,017	-2,216	THBS2	thrombospondin 2	Extracellular Space	other
0	-2,25	ISLR	immunoglobulin superfamily containing leucine-rich repeat	Extracellular Space	other
0,003	-2,414	TSHZ2	teashirt zinc finger homeobox 2	Other	other
0,045	-2,478	HSPB7	heat shock protein family B (small) member 7	Cytoplasm	other
0,023	-2,573	COMP	cartilage oligomeric matrix protein	Extracellular Space	other
0,008	-2,648	CRIP1	cysteine rich protein 1	Cytoplasm	other
0,004	-2,743	FOXQ1	forkhead box Q1	Nucleus	transcription regulator
0,002	-2,837	MN1	meningioma (disrupted in balanced translocation) 1	Nucleus	other
0,03	-2,926	MXRA5	matrix-remodelling associated 5	Extracellular Space	other
0	-3,15	ATOH8	atonal bHLH transcription factor 8	Nucleus	transcription regulator
0,004	-3,661	PTGIS	prostaglandin I2 (prostacyclin) synthase	Cytoplasm	enzyme

Supplementary Table S3. Canonical pathways with changed expression. Results of microarray studies after IPA analysis - comparison 'GD patients vs. Controls' .

A) All cellular canonical pathways with differentially expressed genes.
B) Top five cellular canonical pathways with differentially expressed genes.

**Ingenuity (IPA) Canonical Pathways**	**Molecules**
**A) All cellular canonical pathways with differentially expressed genes.**	
Interferon Signaling	IFIT3, IFIT1, OAS1, IFI6, IRF9, STAT1, TAP1, ISG15
Activation of IRF by Cytosolic Pattern Recognition Receptors	IFIH1, IRF9, STAT1, IFIT2, ISG15
UVA-Induced MAPK Signaling	BCL2L1, TIPARP, PARP4, PARP12, STAT1, PARP9
Retinoic acid Mediated Apoptosis Signaling	TIPARP, PARP4, PARP12, PARP9
Role of Pattern Recognition Receptors in Recognition of Bacteria and Viruses	IFIH1, OAS1, OAS2, CASP1, OAS3
iNOS Signaling	LY96, HMGA1, STAT1
Death Receptor Signaling	TIPARP, PARP4, PARP12, PARP9
Protein Ubiquitination Pathway	UCHL1, B2M, PSME1, DNAJB4, TAP1, UBE2L6, UCHL3
Urate Biosynthesis/Inosine 5'-phosphate Degradation	NT5E, AOX1
Guanosine Nucleotides Degradation III	NT5E, AOX1
IL-15 Production	STAT1, PTK7
RhoGDI Signaling	GNG11, RND3,ITGA2,ARHGEF17,DLC1
Adenosine Nucleotides Degradation II	NT5E,AOX1

Sphingosine-1-phosphate Signaling	S1PR3,RND3,CASP1,SPHK1
Caveolar-mediated Endocytosis Signaling	B2M,ITGA2,CAV1
Oncostatin M Signaling	PLAU,STAT1
Coagulation System	PLAU,TFPI
Hypusine Biosynthesis	EIF5A
Purine Nucleotides Degradation II (Aerobic)	NT5E,AOX1
Antigen Presentation Pathway	B2M,TAP1
PDGF Signaling	CAV1,SPHK1,STAT1
Glutamate Biosynthesis II	GLUD1
Glutamate Degradation X	GLUD1
Tec Kinase Signaling	GNG11,RND3,ITGA2,STAT1
Virus Entry via Endocytic Pathways	B2M,ITGA2,CAV1
CD27 Signaling in Lymphocytes	BCL2L1,MAP3K8
p53 Signaling	CCNG1,BCL2L1,PERP
Semaphorin Signaling in Neurons	SEMA3A,RND3
Actin Nucleation by ARP-WASP Complex	RND3,ITGA2
Triacylglycerol Biosynthesis	LPIN1,ELOVL6
Sertoli Cell-Sertoli Cell Junction Signaling	CLDN11,ITGA2,MAP3K8,PRKG2
Hepatic Fibrosis / Hepatic Stellate Cell Activation	LY96,COL4A5,IGFBP5,STAT1

NRF2-mediated Oxidative Stress Response	MGST1,DNAJB4,DNAJA3,AOX1
Gαi Signaling	S1PR3,GNG11,CAV1
L-carnitine Biosynthesis	ALDH9A1
Glycerol Degradation I	GK
PI3K/AKT Signaling	BCL2L1,ITGA2,MAP3K8
ERK/MAPK Signaling	LAMTOR3,DUSP6,ITGA2,STAT1
Adipogenesis pathway	LPIN1,SMAD9,TXNIP
Parkinson's Signaling	UCHL1
Glioma Invasiveness Signaling	RND3, PLAU
Molecular Mechanisms of Cancer	BCL2L1,LAMTOR3,RND3,SMAD9,ITGA2,ARHGEF17
Pyridoxal 5'-phosphate Salvage Pathway	DMPK,MAP3K8
GM-CSF Signaling	BCL2L1,STAT1
RAN Signaling	XPO1
Extrinsic Prothrombin Activation Pathway	TFPI
Ketogenesis	ACAT1
Ketolysis	ACAT1
LPS/IL-1 Mediated Inhibition of RXR Function	MGST1,LY96,XPO1,ALDH9A1
Sucrose Degradation V (Mammalian)	ALDOC
VDR/RXR Activation	IGFBP5,KLF4

tRNA Charging	NARS,IARS2
JAK/Stat Signaling	BCL2L1,STAT1
Polyamine Regulation in Colon Cancer	PSME1
Role of Lipids/Lipid Rafts in the Pathogenesis of Influenza	RSAD2
Glutaryl-CoA Degradation	ACAT1
Inflammasome pathway	CASP1
Phospholipase C Signaling	GNG11,RND3,ITGA2,ARHGEF17
IL-22 Signaling	STAT1
Role of JAK1, JAK2 and TYK2 in Interferon Signaling	STAT1
Arginine Biosynthesis IV	GLUD1
Huntington's Disease Signaling	BCL2L1,PSME1,GNG11,CASP1
OX40 Signaling Pathway	B2M,BCL2L1
Signaling by Rho Family GTPases	GNG11,RND3,ITGA2,ARHGEF17
Role of JAK family kinases in IL-6-type Cytokine Signaling	STAT1
Fatty Acid α-oxidation	ALDH9A1
Colorectal Cancer Metastasis Signaling	BCL2L1,GNG11,RND3,STAT1
Regulation of Actin-based Motility by Rho	RND3,ITGA2
Lipid Antigen Presentation by CD1	B2M
Glutathione Redox Reactions I	MGST1

Acute Myeloid Leukemia Signaling	IDH2,IDH1
Mevalonate Pathway I	ACAT1
Germ Cell-Sertoli Cell Junction Signaling	RND3,ITGA2,MAP3K8
Ceramide Signaling	S1PR3,SPHK1
G Beta Gamma Signaling	GNG11,CAV1
Isoleucine Degradation I	ACAT1
Cytotoxic T Lymphocyte-mediated Apoptosis of Target Cells	B2M
Mouse Embryonic Stem Cell Pluripotency	SMAD9,ID3
G-Protein Coupled Receptor Signaling	S1PR3,LAMTOR3,DUSP6,MAP3K8
Histamine Degradation	ALDH9A1
Oxidative Ethanol Degradation III	ALDH9A1
Role of JAK2 in Hormone-like Cytokine Signaling	STAT1
Salvage Pathways of Pyrimidine Ribonucleotides	DMPK,MAP3K8
G Protein Signaling Mediated by Tubby	GNG11
MIF-mediated Glucocorticoid Regulation	LY96
NAD Salvage Pathway II	NT5E
Tryptophan Degradation X (Mammalian, via Tryptamine)	ALDH9A1
Putrescine Degradation III	ALDH9A1
Superpathway of Geranylgeranyldiphosphate Biosynthesis	ACAT1

I (via Mevalonate)	
Nucleotide Excision Repair Pathway	ERCC5
Production of Nitric Oxide and Reactive Oxygen Species in Macrophages	RND3,MAP3K8,STAT1
Ethanol Degradation IV	ALDH9A1
IL-8 Signaling	BCL2L1,GNG11,RND3
Notch Signaling	HEY1
HGF Signaling	ITGA2,MAP3K8
Glioma Signaling	IDH2,IDH1
Inhibition of Angiogenesis by TSP1	CD47
Amyotrophic Lateral Sclerosis Signaling	BCL2L1,CASP1
Pancreatic Adenocarcinoma Signaling	BCL2L1,STAT1
PTEN Signaling	BCL2L1,ITGA2
Role of PKR in Interferon Induction and Antiviral Response	STAT1
Transcriptional Regulatory Network in Embryonic Stem Cells	FOXC1
Glutathione-mediated Detoxification	MGST1
Inhibition of Matrix Metalloproteases	THBS2
autophagy	MAP1LC3B
phagosome formation	RND3,ITGA2

Mechanisms of Viral Exit from Host Cells	XPO1
Glycolysis I	ALDOC
RhoA Signaling	RND3,DLC1
Leukocyte Extravasation Signaling	CLDN11,ITGA2,DLC1
Nitric Oxide Signaling in the Cardiovascular System	CAV1,PRKG2
Role of RIG1-like Receptors in Antiviral Innate Immunity	IFIH1
phagosome maturation	B2M,TAP1
LXR/RXR Activation	LY96,MYLIP
MIF Regulation of Innate Immunity	LY96
Netrin Signaling	ABLIM3
Integrin Signaling	RND3,ITGA2,CAV1
IL-9 Signaling	STAT1
Tryptophan Degradation III (Eukaryotic)	ACAT1
Gluconeogenesis I	ALDOC
cAMP-mediated signaling	S1PR3,LAMTOR3,DUSP6
Ethanol Degradation II	ALDH9A1
D-myo-inositol (1,4,5,6)-Tetrakisphosphate Biosynthesis	CILP,UBLCP1
D-myo-inositol (3,4,5,6)-tetrakisphosphate Biosynthesis	CILP,UBLCP1
Primary Immunodeficiency Signaling	TAP1

Dopamine Degradation	ALDH9A1
Stearate Biosynthesis I (Animals)	ELOVL6
Sperm Motility	PRKG2,PTK7
Aryl Hydrocarbon Receptor Signaling	MGST1,ALDH9A1
Cardiac Hypertrophy Signaling	GNG11,RND3,MAP3K8
Human Embryonic Stem Cell Pluripotency	S1PR3,SPHK1
IL-12 Signaling and Production in Macrophages	MAP3K8,STAT1
Noradrenaline and Adrenaline Degradation	ALDH9A1
Docosahexaenoic Acid (DHA) Signaling	BCL2L1
Regulation of Cellular Mechanics by Calpain Protease	ITGA2
3-phosphoinositide Degradation	CILP,UBLCP1
D-myo-inositol-5-phosphate Metabolism	CILP,UBLCP1
Nicotine Degradation III	AOX1
Induction of Apoptosis by HIV1	BCL2L1
CNTF Signaling	STAT1
ERK5 Signaling	MAP3K8
Glutamate Receptor Signaling	GNG11
Hypoxia Signaling in the Cardiovascular System	UBE2L6
Mitotic Roles of Polo-Like Kinase	ANAPC13

Gαq Signaling	GNG11,RND3
Cdc42 Signaling	B2M,ITGA2
Lymphotoxin β Receptor Signaling	BCL2L1
Thrombopoietin Signaling	STAT1
Agrin Interactions at Neuromuscular Junction	ITGA2
CXCR4 Signaling	GNG11,RND3
Role of JAK1 and JAK3 in γc Cytokine Signaling	STAT1
T Helper Cell Differentiation	STAT1
EGF Signaling	STAT1
Dopamine-DARPP32 Feedback in cAMP Signaling	KCNJ2,PRKG2
Ephrin Receptor Signaling	GNG11,ITGA2
Nicotine Degradation II	AOX1
GABA Receptor Signaling	ALDH9A1
Granulocyte Adhesion and Diapedesis	CLDN11,ITGA2
CCR5 Signaling in Macrophages	GNG11
Toll-like Receptor Signaling	LY96
Ephrin B Signaling	GNG11
Gap Junction Signaling	CAV1,PRKG2
Xenobiotic Metabolism Signaling	MGST1,MAP3K8,ALDH9A1

TREM1 Signaling	CASP1
IL-15 Signaling	BCL2L1
BMP signaling pathway	SMAD9
Glucocorticoid Receptor Signaling	BCL2L1, PLAU,STAT1
Role of BRCA1 in DNA Damage Response	STAT1
Protein Kinase A Signaling	DUSP5,GNG11,DUSP6,ANAPC13
Antiproliferative Role of Somatostatin Receptor 2	GNG11
Mitochondrial Dysfunction	PRDX3,NDUFAB1
Role of NFAT in Regulation of the Immune Response	GNG11,XPO1
Agranulocyte Adhesion and Diapedesis	CLDN11,ITGA2
B Cell Receptor Signaling	BCL2L1,MAP3K8
Neurotrophin/TRK Signaling	SPRY2
Dendritic Cell Maturation	B2M,STAT1
IL-3 Signaling	STAT1
Superpathway of Cholesterol Biosynthesis	ACAT1
PEDF Signaling	BCL2L1
FLT3 Signaling in Hematopoietic Progenitor Cells	STAT1
Growth Hormone Signaling	STAT1
Allograft Rejection Signaling	B2M

3-phosphoinositide Biosynthesis	CILP,UBLCP1
Prolactin Signaling	STAT1
NF-κB Activation by Viruses	ITGA2
Small Cell Lung Cancer Signaling	BCL2L1
HIPPO signaling	LATS2
TGF-β Signaling	SMAD9
IL-4 Signaling	HMGA1
Apoptosis Signaling	BCL2L1
Communication between Innate and Adaptive Immune Cells	B2M
mTOR Signaling	RND3,FKBP1A
Serotonin Degradation	ALDH9A1
Reelin Signaling in Neurons	ITGA2
Factors Promoting Cardiogenesis in Vertebrates	SMAD9
GPCR-Mediated Nutrient Sensing in Enteroendocrine Cells	GNG11
Neuregulin Signaling	ITGA2
Thrombin Signaling	GNG11,RND3
Breast Cancer Regulation by Stathmin1	GNG11,ARHGEF17
IL-1 Signaling	GNG11

α-Adrenergic Signaling	GNG11
CTLA4 Signaling in Cytotoxic T Lymphocytes	B2M
FAK Signaling	ITGA2
RANK Signaling in Osteoclasts	MAP3K8
CDK5 Signaling	ITGA2
PAK Signaling	ITGA2
Chronic Myeloid Leukemia Signaling	BCL2L1
SAPK/JNK Signaling	GNG11
Cholecystokinin/Gastrin-mediated Signaling	RND3
**B) Top five cellular canonical pathways with differentially expressed genes.**	
Interferon Signaling	IFIT3, IFIT1, OAS1, IFI6, IRF9, STAT1, TAP1, ISG15
Activation of IRF by Cytosolic Pattern Recognition Receptors	IFIH1, IRF9, STAT1, IFIT2, ISG15
UVA-Induced MAPK Signaling	BCL2L1, TIPARP, PARP4, PARP12, STAT1, PARP9
Retinoic acid Mediated Apoptosis Signaling	TIPARP, PARP4, PARP12, PARP9
Role of Pattern Recognition Receptors in Recognition of Bacteria and Viruses	IFIH1, OAS1, OAS2, CASP1, OAS3

Supplementary Table S4. Canonical pathways with changed expression. Results of microarray studies after IPA analysis - Comparison 'GD patients vs. NPC patients'.

A) All cellular canonical pathways with differentially expressed genes.
B) Top five cellular canonical pathways with differentially expressed genes.

**Ingenuity Canonical Pathways**	**Molecules**
**A) All cellular canonical pathways with differentially expressed genes.**	
tRNA Charging	NARS,LARS,WARS,IARS2,YARS,GARS,TARS,MARS,IARS,EPRS
Interferon Signaling	IFIT3,**IFIT1***,OAS1,**MX1**,STAT1,TAP1,**ISG15**
Protein Ubiquitination Pathway	PSMA6,DNAJB4,HSPA9,PSME2,TAP1,UCHL3,**UCHL1**,USO1,HSP90B1,HLA-C,PSMA4,HSPB7,PSMD14,PSMC2,UBE2E1
Vitamin-C Transport	SLC2A1,NXN,GLRX
RAN Signaling	KPNB1,KPNA4,XPO1
Pyruvate Fermentation to Lactate	LDHA,LDHB
Polyamine Regulation in Colon Cancer	MYC,PSME2,ODC1
Activation of IRF by Cytosolic Pattern Recognition Receptors	STAT1,NFKB1,IFIT2,**ISG15**
Colorectal Cancer Metastasis Signaling	MYC,**FOS**,RND3,RHOB,VEGFC,MMP2,STAT1,NFKB1,WNT5A
Aryl Hydrocarbon Receptor Signaling	MYC,**FOS**,MGST1,HSP90B1,HSPB7,NFKB1
Glioma Invasiveness Signaling	RND3,RHOB,MMP2,**PLAU**
Hepatic Fibrosis / Hepatic Stellate Cell Activation	COL4A5,KLF6,SMAD7,VEGFC,MMP2,STAT1,NFKB1
Methionine Degradation I (to Homocysteine)	PRMT3,AHCY
RAR Activation	**FOS**,SMAD9,RDH14,SMAD7,SMAD6,NFKB1,CITED2
Guanine and Guanosine Salvage I	HPRT1
Alanine Degradation III	GPT2
Alanine Biosynthesis II	GPT2
Putrescine Biosynthesis III	ODC1
iNOS Signaling	**FOS**,STAT1,NFKB1
BMP signaling pathway	SMAD9,SMAD7,SMAD6,NFKB1
Glutathione Redox Reactions I	MGST1,PRDX6
Cysteine Biosynthesis III (mammalia)	PRMT3,AHCY
HIF1α Signaling	SLC2A1,VEGFC,MMP2,LDHA,LDHB

Ascorbate Recycling (Cytosolic)	GLRX
Tetrahydrobiopterin Biosynthesis I	PTS
Inosine-5'-phosphate Biosynthesis II	PAICS
Hypusine Biosynthesis	EIF5A
Thiosulfate Disproportionation III (Rhodanese)	TST
Tetrahydrobiopterin Biosynthesis II	PTS
CD27 Signaling in Lymphocytes	**FOS**,MAP3K8,NFKB1
Regulation of Actin-based Motility by Rho	ACTR3,RND3,RHOB,PFN2
Semaphorin Signaling in Neurons	SEMA3A,RND3,RHOB
Glycolysis I	TPI1,PGAM1
TGF-β Signaling	**FOS**,SMAD9,SMAD7,SMAD6
PPAR Signaling	**FOS**,HSP90B1,NFKB1,CITED2
Role of JAK1, JAK2 and TYK2 in Interferon Signaling	STAT1,NFKB1
Gluconeogenesis I	PGAM1,ME1
Actin Nucleation by ARP-WASP Complex	ACTR3,RND3,RHOB
IL-17A Signaling in Gastric Cells	**FOS**,NFKB1
Glutathione Redox Reactions II	GLRX
Antioxidant Action of Vitamin C	SLC2A1,NXN,NFKB1,GLRX
IL-15 Production	STAT1,NFKB1
TNFR2 Signaling	**FOS**,NFKB1
Tetrahydrofolate Salvage from 5,10-methenyltetrahydrofolate	MTHFD2
Thioredoxin Pathway	NXN
Glycerol Degradation I	GK
Citrulline-Nitric Oxide Cycle	ASS1
Rapoport-Luebering Glycolytic Shunt	PGAM1
Molecular Mechanisms of Cancer	MYC,**FOS**,PMAIP1,RND3,SMAD9,RHOB,SMAD7,SMAD6,NFKB1,WNT5A
ERK5 Signaling	MYC,**FOS**,MAP3K8
Hypoxia Signaling in the Cardiovascular System	HSP90B1,LDHA,UBE2E1
Pyrimidine Ribonucleotides Interconversion	NME1,CTPS1
Remodeling of Epithelial Adherens Junctions	NME1,ACTR3,MAPRE1

Thrombopoietin Signaling	MYC,**<u>FOS</u>**,STAT1
Production of Nitric Oxide and Reactive Oxygen Species in Macrophages	**<u>FOS</u>**,RND3,RHOB,MAP3K8,STAT1,NFKB1
ILK Signaling	MYC,**<u>FOS</u>**,RND3,RHOB,VEGFC,NFKB1
Superpathway of Methionine Degradation	PRMT3,AHCY
Mouse Embryonic Stem Cell Pluripotency	MYC,ID1,SMAD9,ID3
Arginine Biosynthesis IV	ASS1
Urea Cycle	ASS1
UDP-N-acetyl-D-glucosamine Biosynthesis II	GFPT1
Adenine and Adenosine Salvage III	HPRT1
Role of JAK2 in Hormone-like Cytokine Signaling	SH2B3,STAT1
Pyrimidine Ribonucleotides De Novo Biosynthesis	NME1,CTPS1
IL-8 Signaling	**<u>FOS</u>**,RND3,RHOB,VEGFC,MMP2,NFKB1
Mitochondrial Dysfunction	ATP5C1,PRDX3,NDUFAF2,NDUFAB1,NDUFA8
Oncostatin M Signaling	**<u>PLAU</u>**,STAT1
Coagulation System	PROS1,**<u>PLAU</u>**
IL-17A Signaling in Fibroblasts	**<u>FOS</u>**,NFKB1
Antigen Presentation Pathway	HLA-C,TAP1
RhoA Signaling	ACTR3,RND3,PFN2,DLC1
p38 MAPK Signaling	MYC,DUSP10,HSPB7,STAT1
April Mediated Signaling	**<u>FOS</u>**,NFKB1
Tec Kinase Signaling	**<u>FOS</u>**,RND3,RHOB,STAT1,NFKB1
Airway Pathology in Chronic Obstructive Pulmonary Disease	MMP2
Sucrose Degradation V (Mammalian)	TPI1
Histidine Degradation III	MTHFD2
GDP-glucose Biosynthesis	PGM1
Aldosterone Signaling in Epithelial Cells	HSP90B1,DNAJB4,HSPA9,HSPB7,AHCY
Inhibition of Matrix Metalloproteases	THBS2,MMP2
B Cell Activating Factor Signaling	**<u>FOS</u>**,NFKB1
Glucose and Glucose-1-phosphate Degradation	PGM1
Prostanoid Biosynthesis	**<u>PTGIS</u>**

Folate Transformations I	MTHFD2
Role of PKR in Interferon Induction and Antiviral Response	STAT1,NFKB1
MIF Regulation of Innate Immunity	**FOS**,NFKB1
Glycogen Degradation II	PGM1
Methylglyoxal Degradation III	AKR1B1
Prolactin Signaling	MYC,**FOS**,STAT1
JAK/Stat Signaling	**FOS**,STAT1,NFKB1
HMGB1 Signaling	**FOS**,RND3,RHOB,NFKB1
Purine Nucleotides De Novo Biosynthesis II	PAICS
Bladder Cancer Signaling	MYC,VEGFC,MMP2
IL-9 Signaling	STAT1,NFKB1
Role of Pattern Recognition Receptors in Recognition of Bacteria and Viruses	OAS1,OAS2,OAS3,NFKB1
Glucocorticoid Receptor Signaling	**FOS**,HSP90B1,HSPA9,POLR2H,**PLAU**,STAT1,NFKB1
TNFR1 Signaling	**FOS**,NFKB1
PDGF Signaling	MYC,**FOS**,STAT1
Death Receptor Signaling	HSPB7,PARP12,NFKB1
OX40 Signaling Pathway	HLA-C,NFKB1
Assembly of RNA Polymerase III Complex	GTF3A
Glycogen Degradation III	PGM1
Oxidative Phosphorylation	ATP5C1,NDUFAB1,NDUFA8
Superpathway of Citrulline Metabolism	ASS1
IL-12 Signaling and Production in Macrophages	**FOS**,MAP3K8,STAT1,NFKB1
Unfolded protein response	HSP90B1,HSPA9
TR/RXR Activation	SLC16A3,SLC2A1,ME1
RANK Signaling in Osteoclasts	**FOS**,MAP3K8,NFKB1
Cholecystokinin/Gastrin-mediated Signaling	**FOS**,RND3,RHOB
UVA-Induced MAPK Signaling	**FOS**,PARP12,STAT1
ERK/MAPK Signaling	MYC,**FOS**,DUSP6,HSPB7,STAT1
Type I Diabetes Mellitus Signaling	HLA-C,STAT1,NFKB1
Wnt/Ca+ pathway	NFKB1,WNT5A

Extrinsic Prothrombin Activation Pathway	PROS1
Parkinson's Signaling	**UCHL1**
Granzyme A Signaling	NME1
Glioblastoma Multiforme Signaling	MYC,RND3,RHOB,WNT5A
phagosome formation	SCARA3,RND3,RHOB
Gαq Signaling	RGS2,RND3,RHOB,NFKB1
Cardiomyocyte Differentiation via BMP Receptors	SMAD6
Role of Lipids/Lipid Rafts in the Pathogenesis of Influenza	RSAD2
The Visual Cycle	RDH14
Tryptophan Degradation X (Mammalian, via Tryptamine)	RDH14
Mitotic Roles of Polo-Like Kinase	HSP90B1,ANAPC13
PCP pathway	PFN2,WNT5A
Axonal Guidance Signaling	SEMA3A,ADAMTS6,ACTR3,ADAM19,PFN2,VEGFC,PSMD14,MMP2,WNT5A
Endoplasmic Reticulum Stress Pathway	HSP90B1
Rac Signaling	ACTR3,NFKB1,NCKAP1
Inflammasome pathway	NFKB1
CD28 Signaling in T Helper Cells	**FOS**,ACTR3,NFKB1
RhoGDI Signaling	ACTR3,RND3,RHOB,DLC1
Renin-Angiotensin Signaling	**FOS**,STAT1,NFKB1
phagosome maturation	HLA-C,TAP1,PRDX6
IL-10 Signaling	**FOS,**NFKB1
EGF Signaling	**FOS**,STAT1
Pancreatic Adenocarcinoma Signaling	VEGFC,STAT1,NFKB1
PKCθ Signaling in T Lymphocytes	**FOS**,MAP3K8,NFKB1
D-myo-inositol (1,4,5,6)-Tetrakisphosphate Biosynthesis	DUSP10,PPP4R1,SACM1L
D-myo-inositol (3,4,5,6)-tetrakisphosphate Biosynthesis	DUSP10,PPP4R1,SACM1L
PI3K/AKT Signaling	HSP90B1,MAP3K8,NFKB1
Role of Osteoblasts, Osteoclasts and Chondrocytes in	**FOS**,SMAD9,SMAD6,NFKB1,WNT5A

Rheumatoid Arthritis	
Pyrimidine Deoxyribonucleotides De Novo Biosynthesis I	NME1
Cdc42 Signaling	**FOS,**ACTR3,HLA-C
Toll-like Receptor Signaling	**FOS**,NFKB1
IL-22 Signaling	STAT1
Estrogen-mediated S-phase Entry	MYC
Glutathione-mediated Detoxification	MGST1
Role of JAK family kinases in IL-6-type Cytokine Signaling	STAT1
GNRH Signaling	**FOS,**MAP3K8,NFKB1
IL-6 Signaling	**FOS**,HSPB7,NFKB1
Regulation of the Epithelial-Mesenchymal Transition Pathway	TWIST1,MMP2,NFKB1,WNT5A
Regulation of IL-2 Expression in Activated and Anergic T Lymphocytes	**FOS**,NFKB1
Cytotoxic T Lymphocyte-mediated Apoptosis of Target Cells	HLA-C
Role of Macrophages, Fibroblasts and Endothelial Cells in Rheumatoid Arthritis	MYC,**FOS**,VEGFC,NFKB1,PRSS3,WNT5A
Estrogen-Dependent Breast Cancer Signaling	**FOS**,NFKB1
Neurotrophin/TRK Signaling	**FOS**,SPRY2
P2Y Purigenic Receptor Signaling Pathway	MYC,**FOS**,NFKB1
CD40 Signaling	**FOS**,NFKB1
Signaling by Rho Family GTPases	**FOS**,ACTR3,RND3,RHOB,NFKB1
Renal Cell Carcinoma Signaling	**FOS**,SLC2A1
NRF2-mediated Oxidative Stress Response	**FOS**,MGST1,DNAJB4,DNAJA3
Intrinsic Prothrombin Activation Pathway	PROS1
Erythropoietin Signaling	**FOS**,NFKB1
3-phosphoinositide Degradation	DUSP10,PPP4R1,SACM1L
D-myo-inositol-5-phosphate Metabolism	DUSP10,PPP4R1,SACM1L
Retinoate Biosynthesis I	RDH14

Growth Hormone Signaling	**FOS,**STAT1
Ethanol Degradation II	RDH14
Human Embryonic Stem Cell Pluripotency	SMAD7,SMAD6,WNT5A
IL-3 Signaling	**FOS**,STAT1
4-1BB Signaling in T Lymphocytes	NFKB1
Triacylglycerol Degradation	PRDX6
Small Cell Lung Cancer Signaling	MYC,NFKB1
Neuregulin Signaling	MYC,HSP90B1
Autoimmune Thyroid Disease Signaling	HLA-C
MIF-mediated Glucocorticoid Regulation	NFKB1
Ovarian Cancer Signaling	VEGFC,MMP2,WNT5A
LPS-stimulated MAPK Signaling	**FOS**,NFKB1
VEGF Family Ligand-Receptor Interactions	**FOS**,VEGFC
TWEAK Signaling	NFKB1
Noradrenaline and Adrenaline Degradation	RDH14
Crosstalk between Dendritic Cells and Natural Killer Cells	HLA-C,NFKB1
Acute Myeloid Leukemia Signaling	MYC,NFKB1
Primary Immunodeficiency Signaling	TAP1
Xenobiotic Metabolism Signaling	MGST1,HSP90B1,MAP3K8,NFKB1,CITED2
eNOS Signaling	HSP90B1,HSPA9,VEGFC
IL-1 Signaling	**FOS,**NFKB1
Cell Cycle Regulation by BTG Family Proteins	BTG2
Nucleotide Excision Repair Pathway	POLR2H
Ceramide Signaling	**FOS,**NFKB1
Integrin Signaling	ACTR3,RND3,RHOB,PFN2
Prostate Cancer Signaling	HSP90B1,NFKB1
Salvage Pathways of Pyrimidine Ribonucleotides	NME1,MAP3K8
Retinol Biosynthesis	RDH14
Notch Signaling	HEY1
Complement System	C1QBP

Allograft Rejection Signaling	HLA-C
Graft-versus-Host Disease Signaling	HLA-C
Thyroid Cancer Signaling	MYC
Cancer Drug Resistance By Drug Efflux	NFKB1
Mechanisms of Viral Exit from Host Cells	XPO1
Neuroprotective Role of THOP1 in Alzheimer's Disease	HLA-C
VEGF Signaling	VEGFC,EIF2B2
Role of RIG1-like Receptors in Antiviral Innate Immunity	NFKB1
Role of IL-17F in Allergic Inflammatory Airway Diseases	NFKB1
Serotonin Receptor Signaling	PTS
Tight Junction Signaling	**FOS**,CLDN1,NFKB1
Virus Entry via Endocytic Pathways	HLA-C,TFRC
Chronic Myeloid Leukemia Signaling	MYC,NFKB1
UVC-Induced MAPK Signaling	**FOS**
CXCR4 Signaling	**FOS**,RND3,RHOB
Wnt/β-catenin Signaling	MYC,APPL2,WNT5A
PPARα/RXRα Activation	HSP90B1,GK,NFKB1
Germ Cell-Sertoli Cell Junction Signaling	RND3,RHOB,MAP3K8
Cardiac Hypertrophy Signaling	RND3,RHOB,MAP3K8,EIF2B2
T Cell Receptor Signaling	**FOS**,NFKB1
Role of NFAT in Regulation of the Immune Response	**FOS**,XPO1,NFKB1
Dendritic Cell Maturation	HLA-C,STAT1,NFKB1
Telomerase Signaling	MYC,HSP90B1
Nitric Oxide Signaling in the Cardiovascular System	HSP90B1,VEGFC
Androgen Signaling	POLR2H,NFKB1
3-phosphoinositide Biosynthesis	DUSP10,PPP4R1,SACM1L
p53 Signaling	PMAIP1,PERP
Assembly of RNA Polymerase II Complex	POLR2H
HGF Signaling	**FOS**,MAP3K8
Heparan Sulfate Biosynthesis (Late Stages)	PRDX6

NGF Signaling	MAP3K8,NFKB1
Systemic Lupus Erythematosus Signaling	**FOS**,HLA-C,PRPF4
Sphingosine-1-phosphate Signaling	RND3,RHOB
fMLP Signaling in Neutrophils	ACTR3,NFKB1
Role of NANOG in Mammalian Embryonic Stem Cell Pluripotency	SMAD9,WNT5A
PI3K Signaling in B Lymphocytes	**FOS,**NFKB1
Heparan Sulfate Biosynthesis	PRDX6
Retinoic acid Mediated Apoptosis Signaling	PARP12
Induction of Apoptosis by HIV1	NFKB1
CNTF Signaling	STAT1
Serotonin Degradation	RDH14
T Helper Cell Differentiation	STAT1
**B) Top five cellular canonical pathways with differentially expressed genes.**	
tRNA Charging	NARS, LARS, WARS, IARS2, YARS, GARS, TARS, MARS, IARS, EPRS
Interferon Signaling	IFIT3, **IFIT1***, OAS1, MX1, STAT1, TAP1, ISG15
Protein Ubiquitination Pathway	PSMA6, DNAJB4, HSPA9, PSME2, TAP1, UCHL3, UCHL1, USO1, HSP90B1, HLA-C, PSMA4, HSPB7, PSMD14, PSMC2, UBE2E1
Vitamin-C Transport	SLC2A1, NXN, GLRX
RAN Signaling	KPNB1, KPNA4, XPO1

*bolded and underlined are genes with most enhanced or inhibited expression after microarray study

Supplementary Figure S5 . Networks of the differentially expressed genes by using IPA platform for the comparison: GD patients vs. Controls.

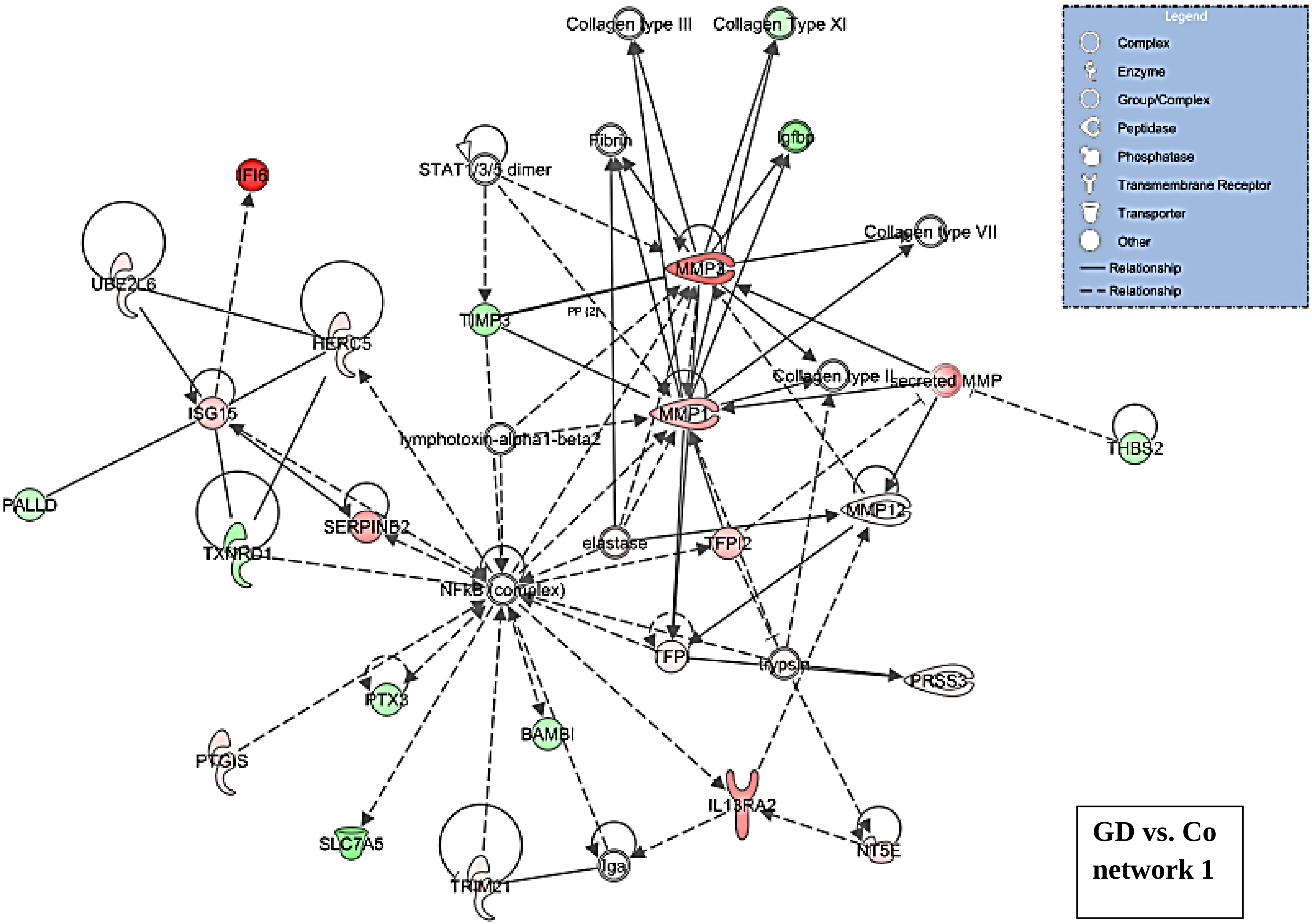

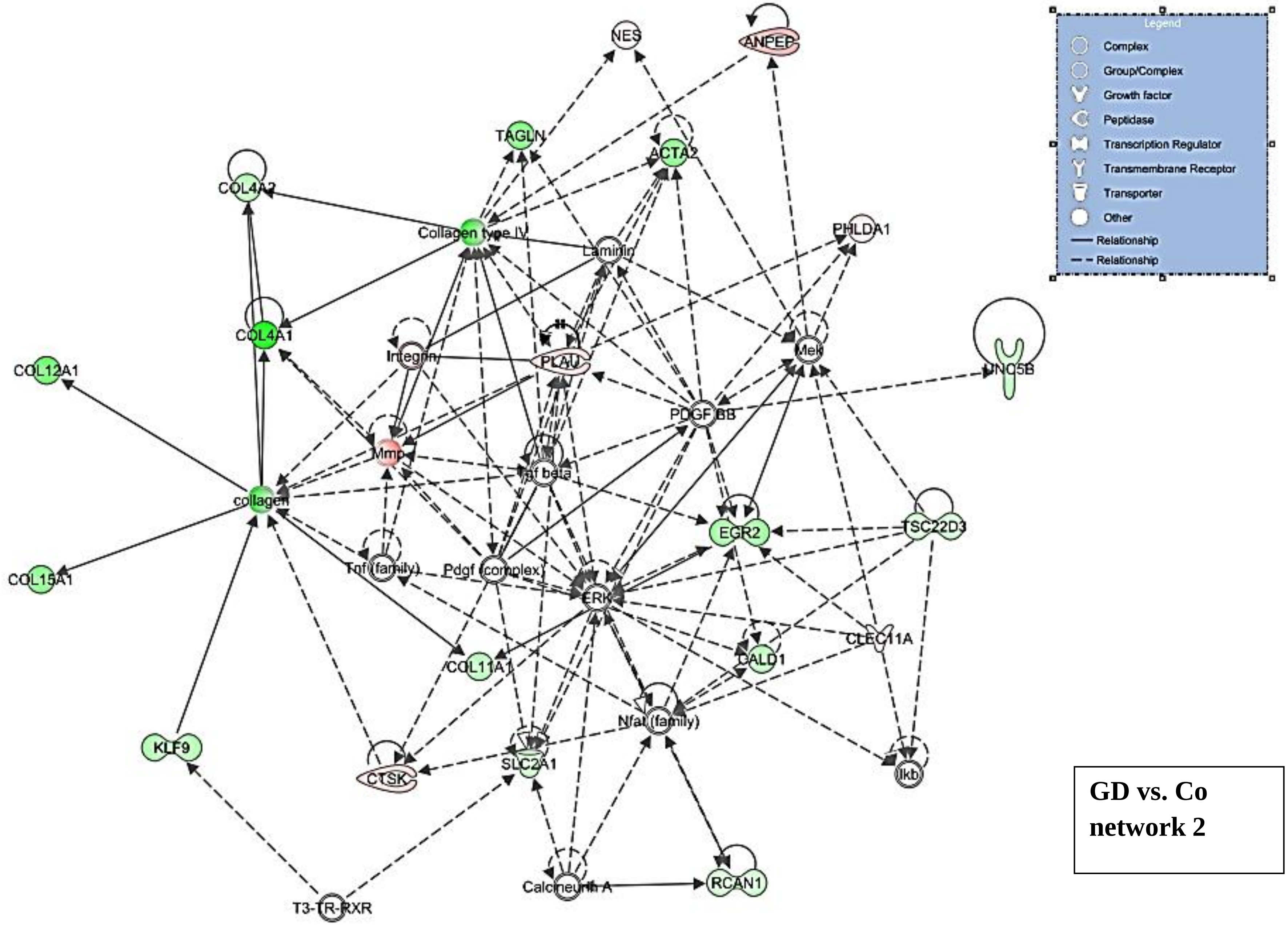

GD vs. Co network 2

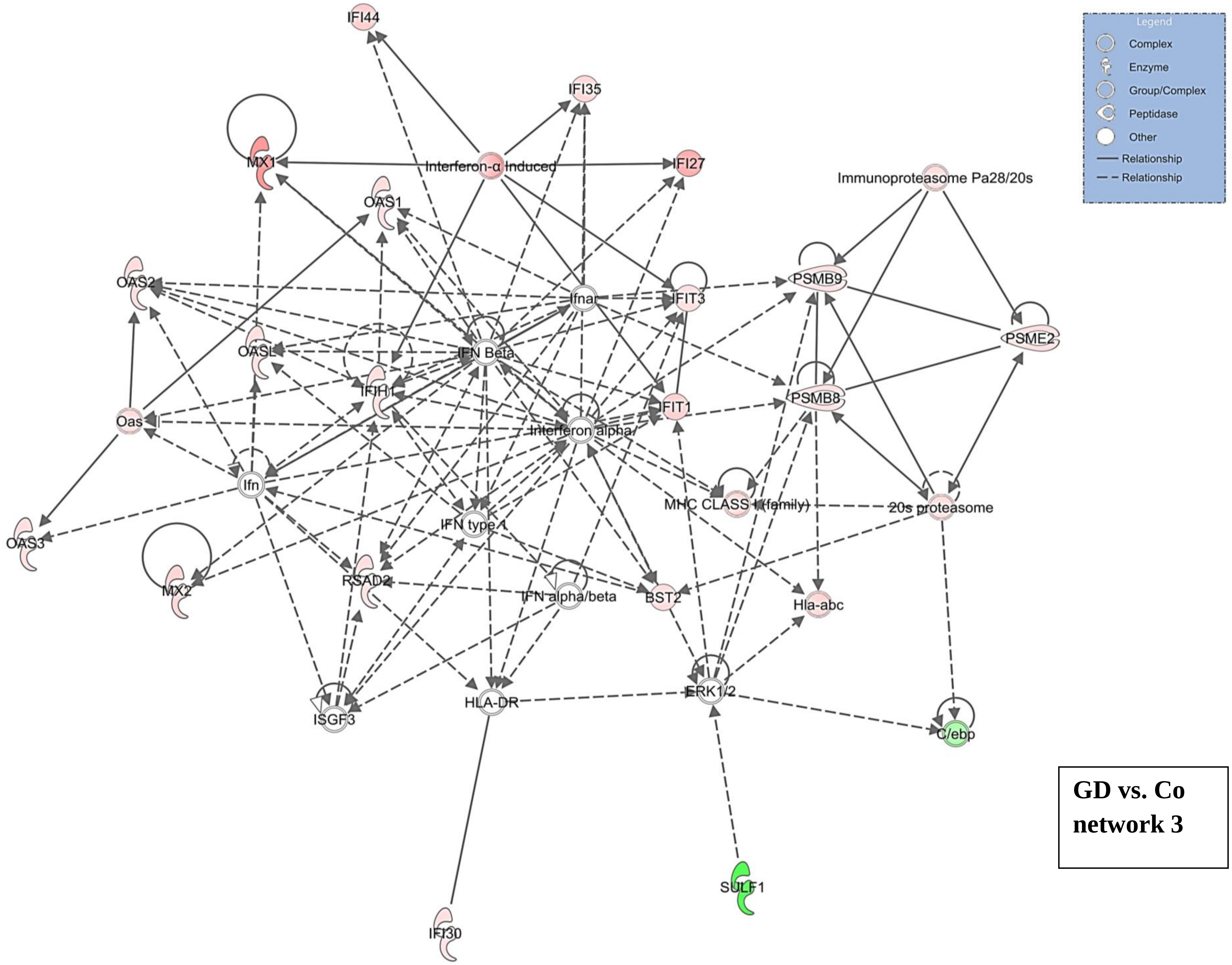

GD vs. Co network 3

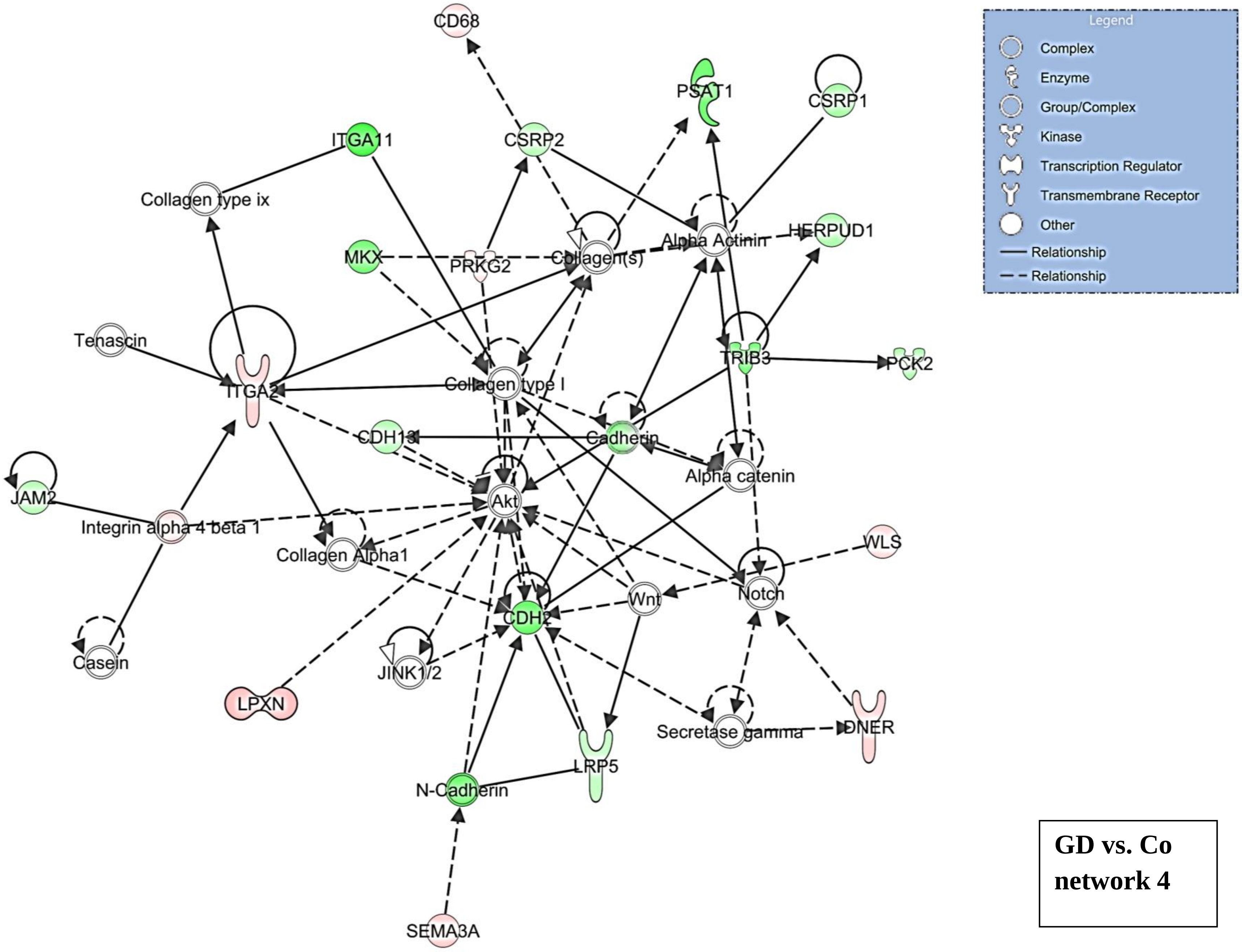

GD vs. Co network 4

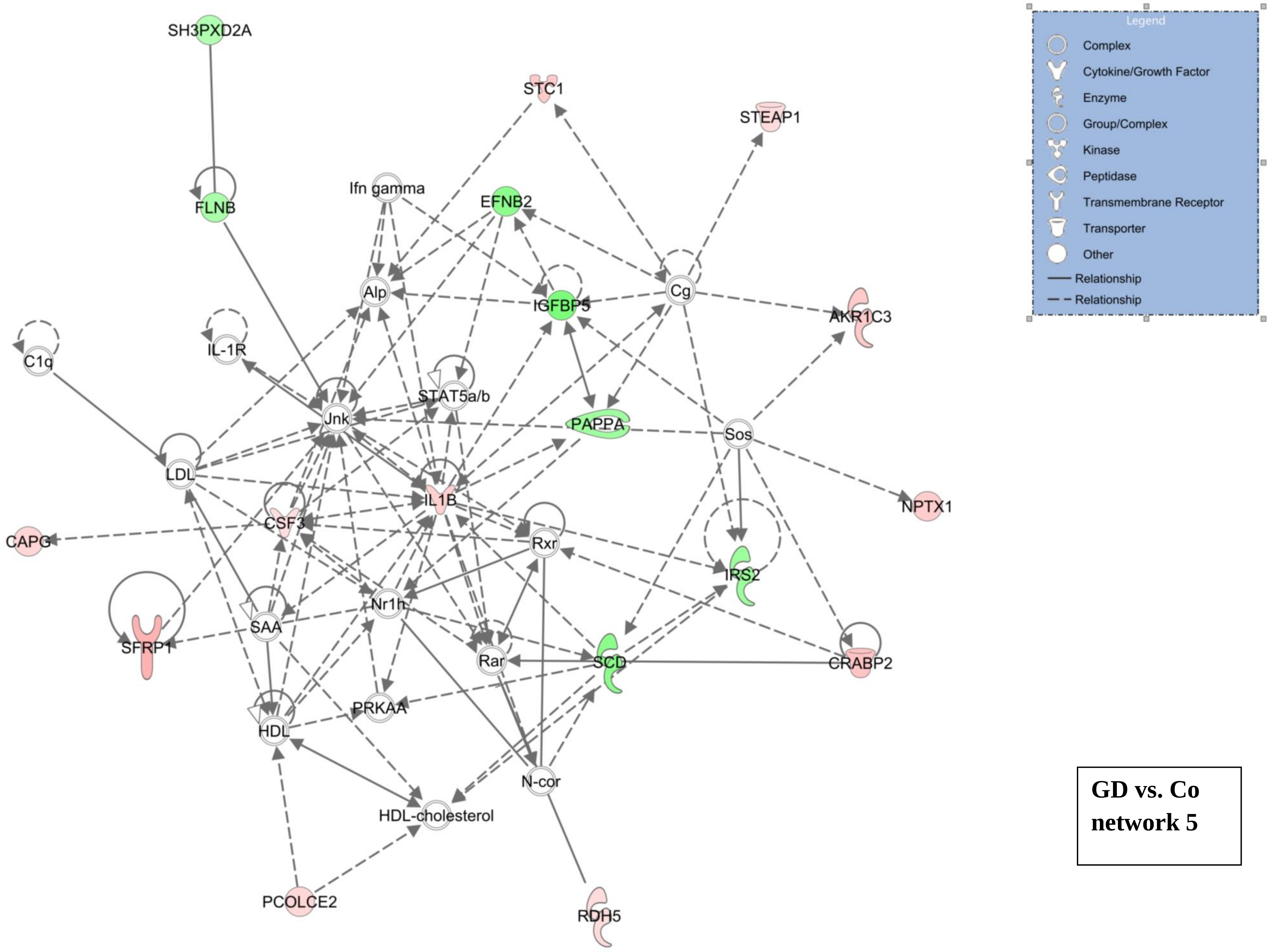

GD vs. Co network 5

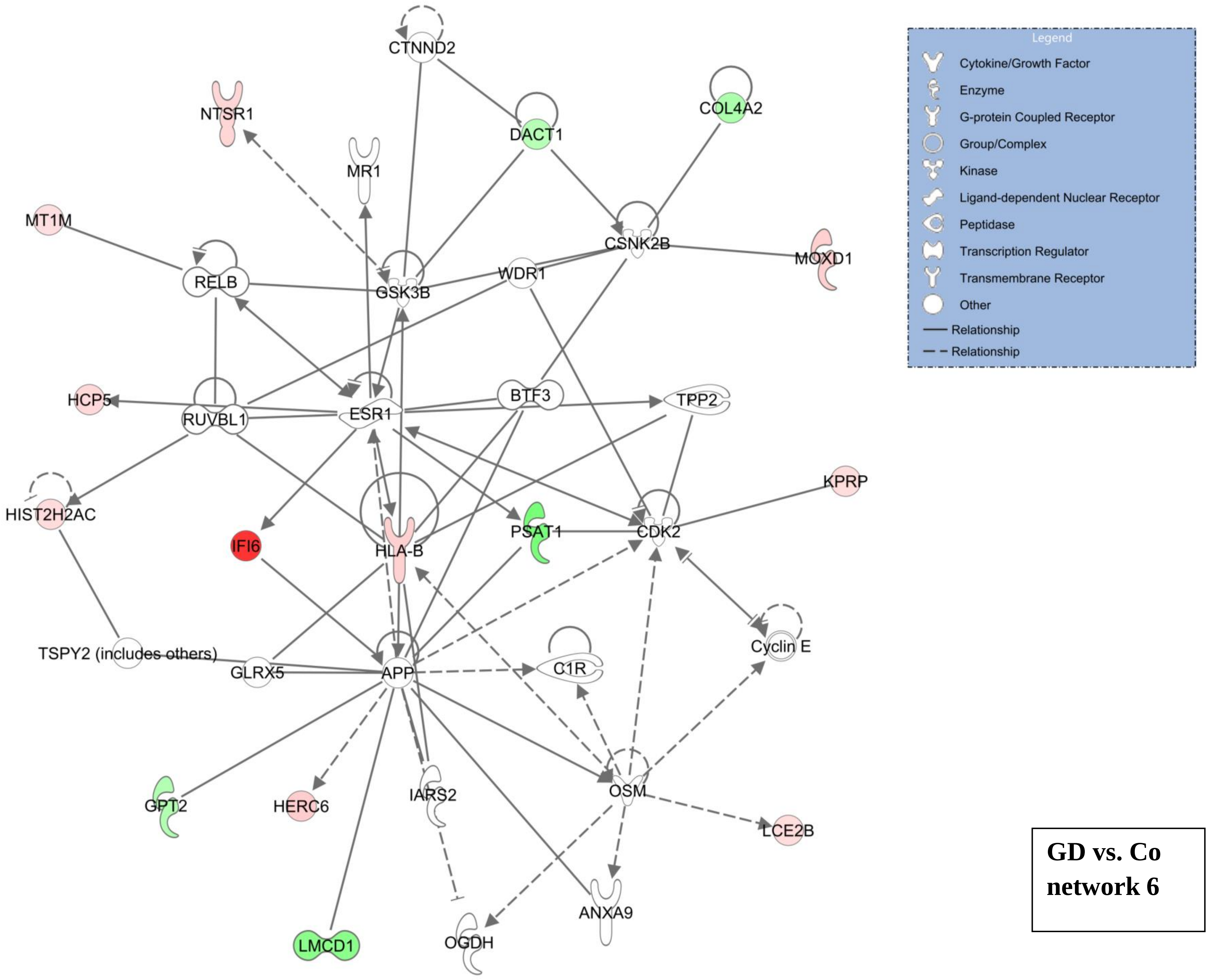

GD vs. Co network 6

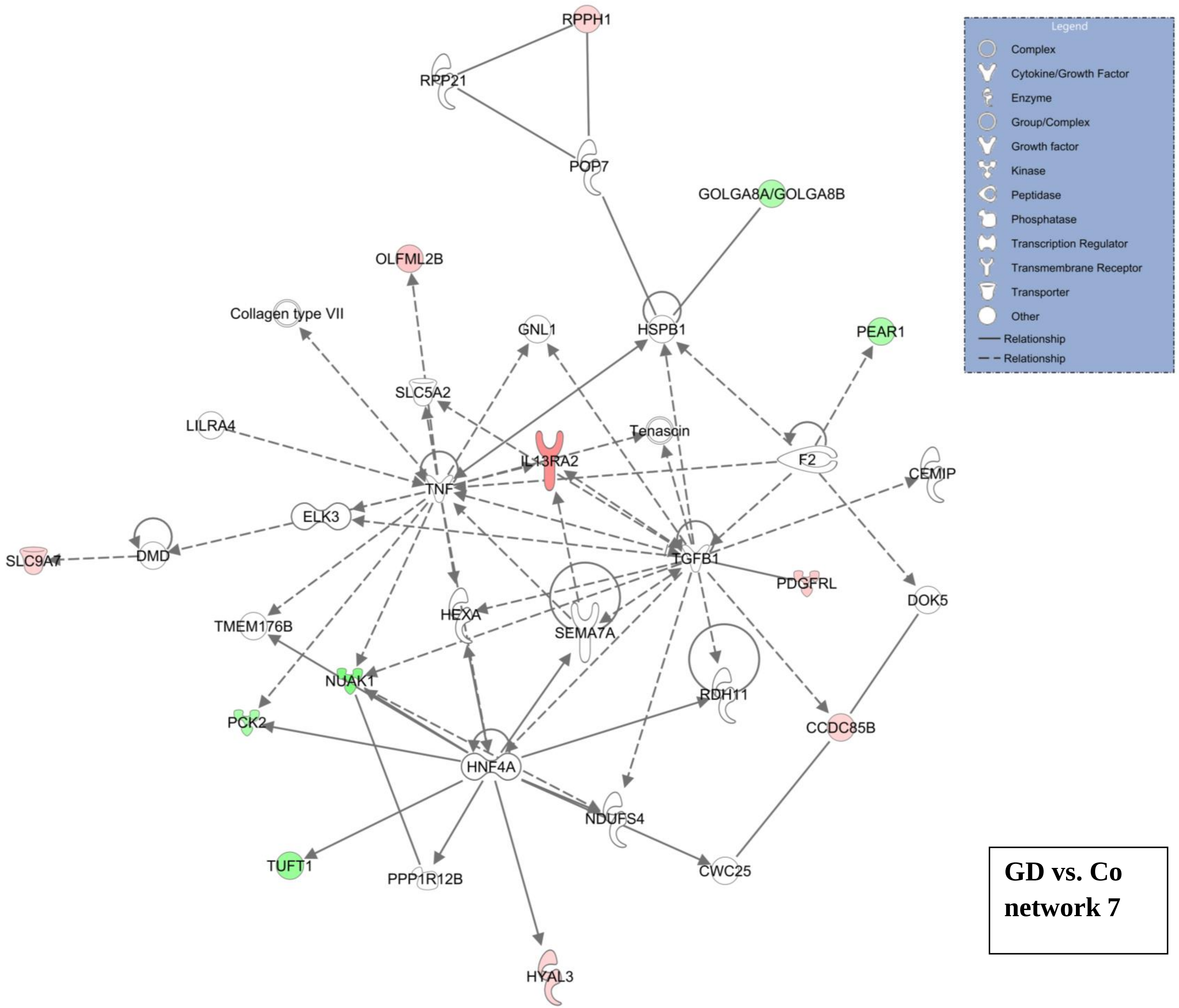

GD vs. Co network 7

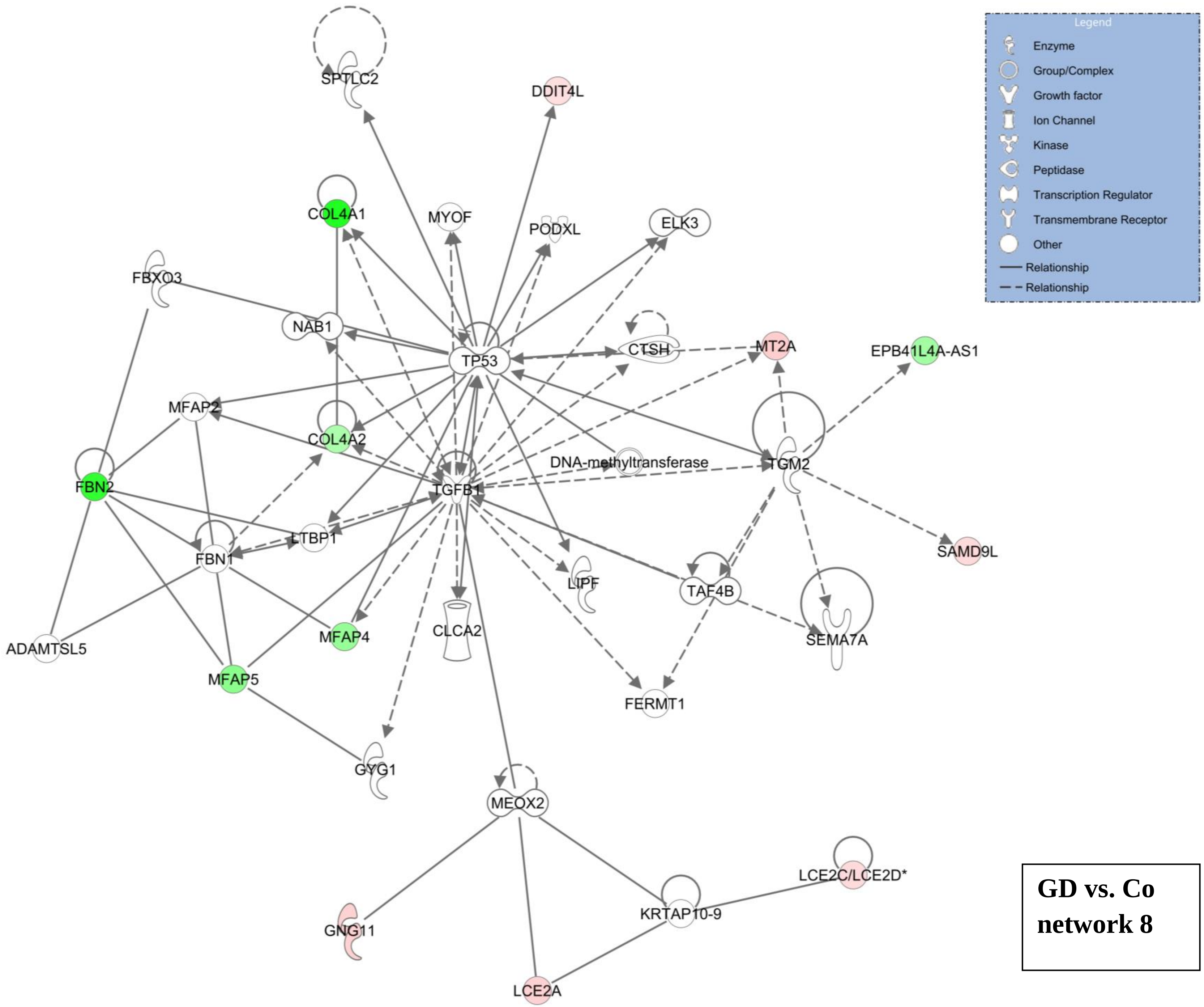

GD vs. Co network 8

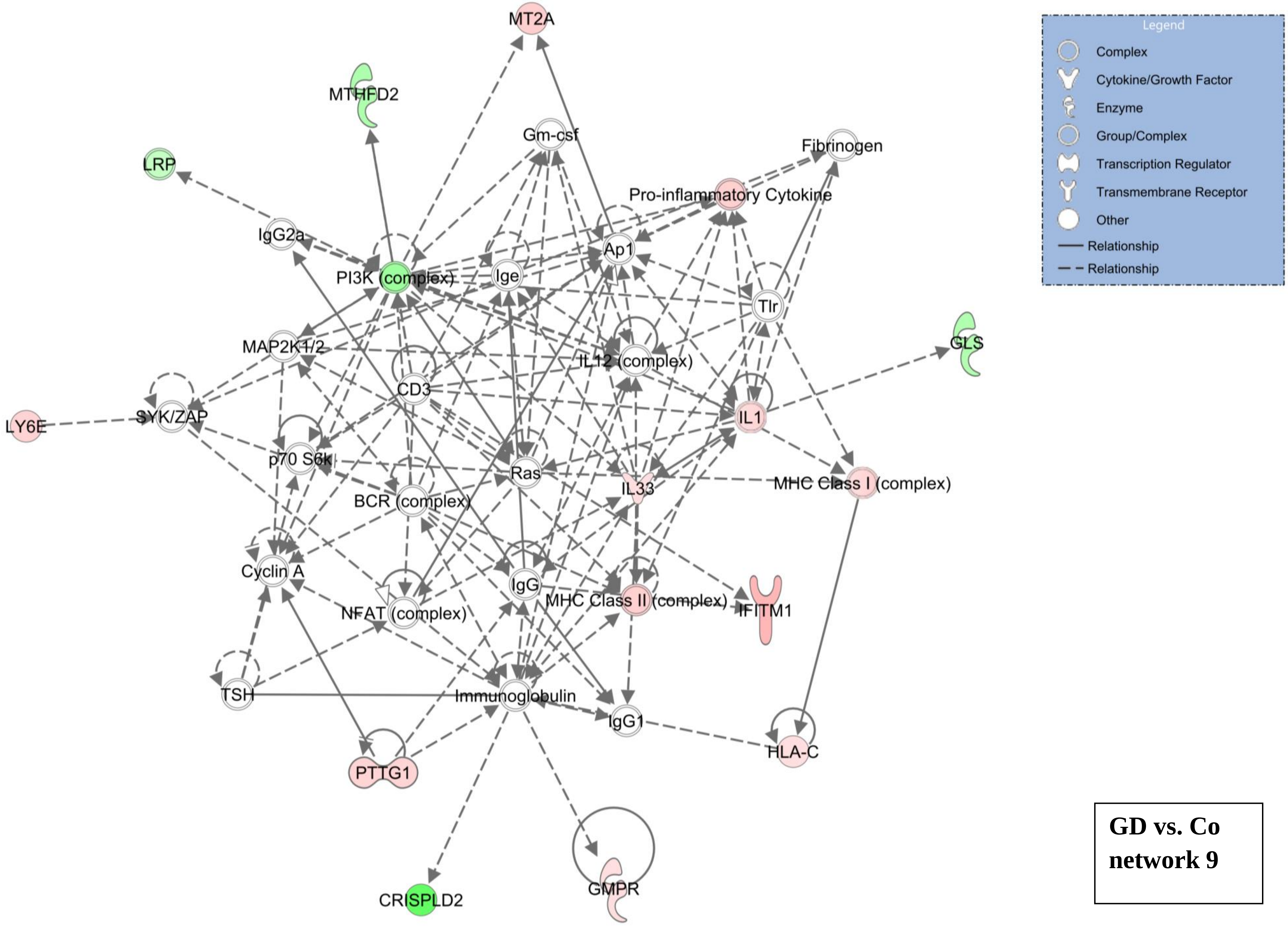

GD vs. Co network 9

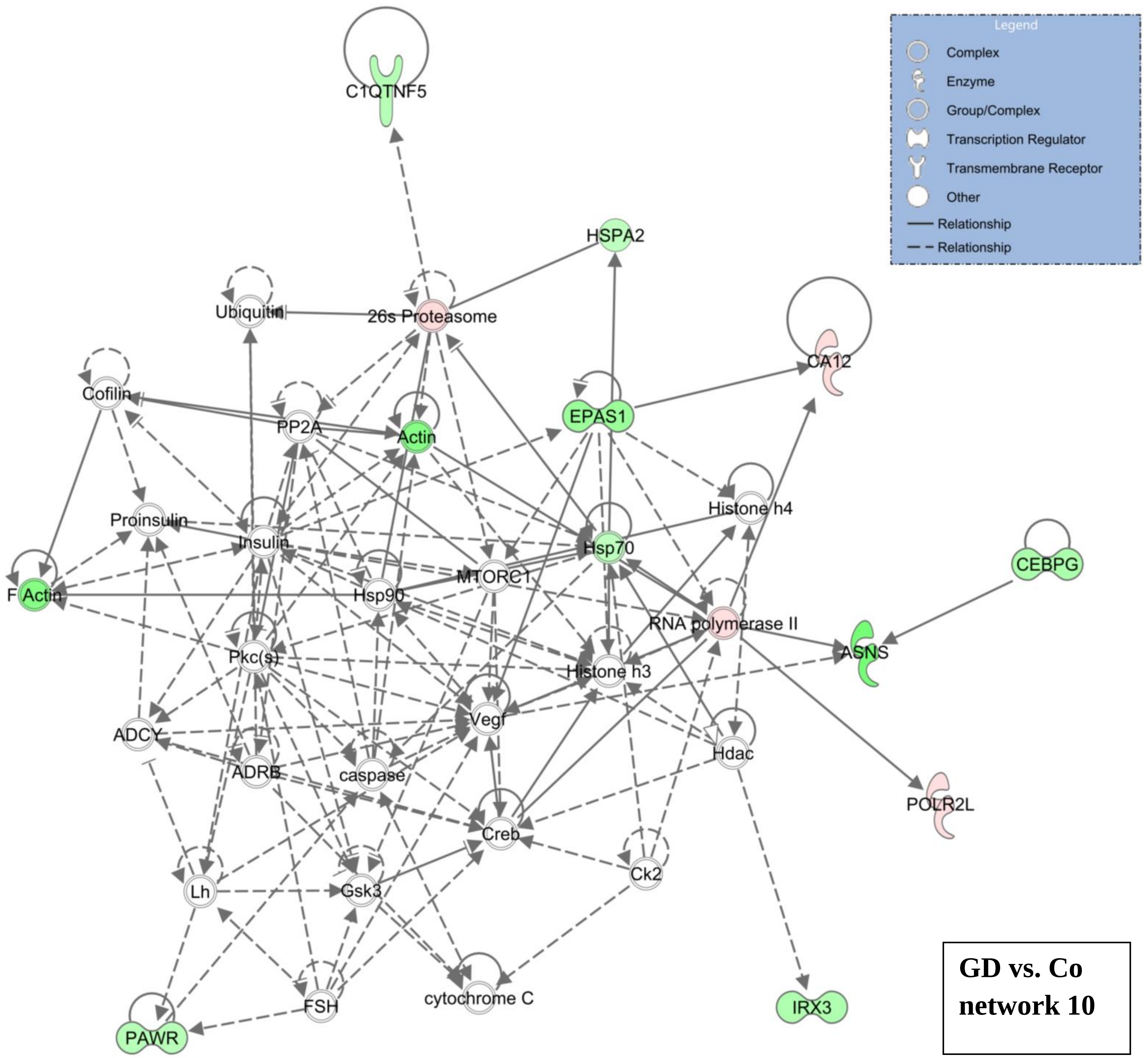

GD vs. Co network 10

GD vs. Co
network 11

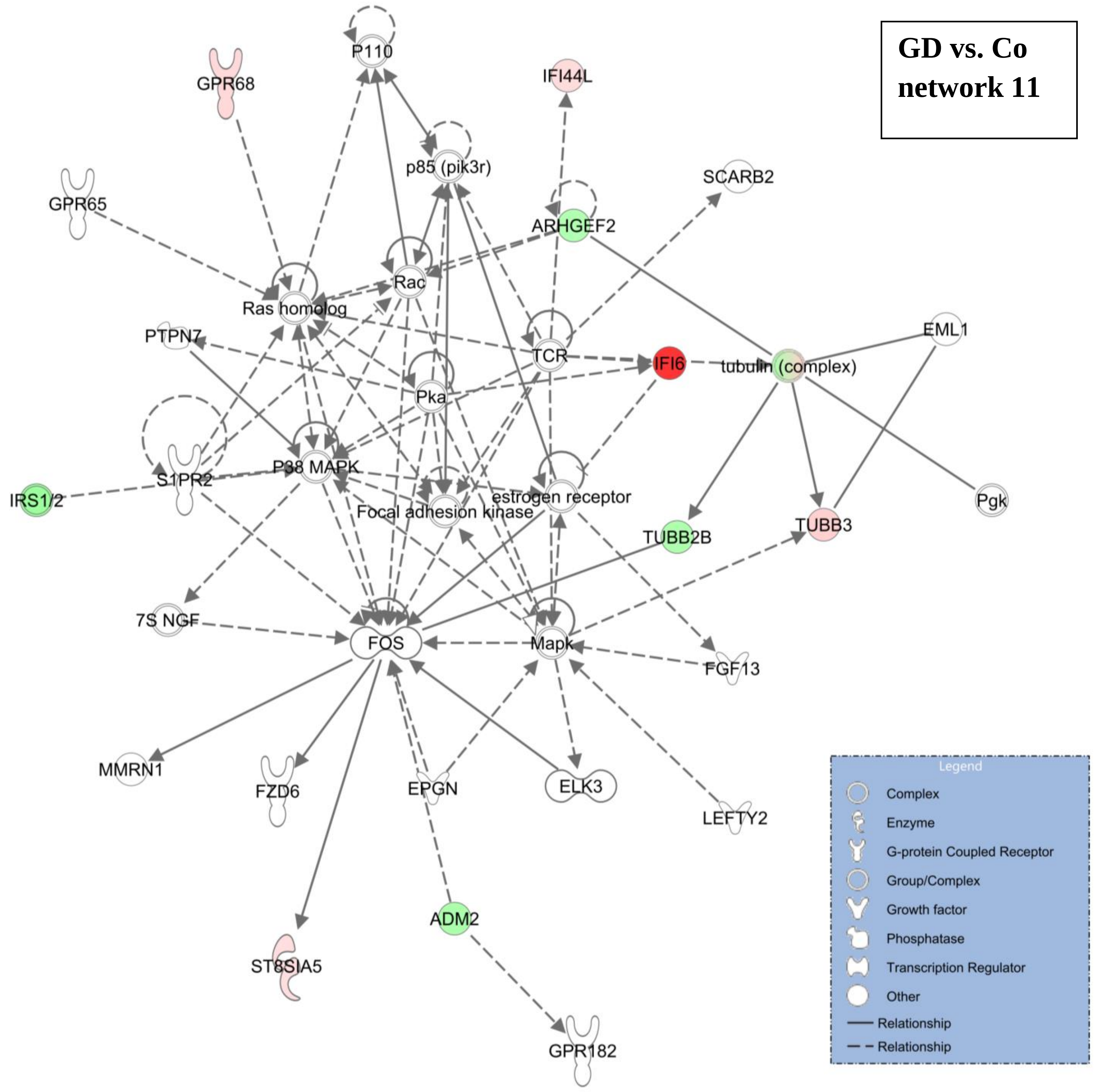

Supplementary Table S6. Networks of molecules in interactions for the differentially expressed genes by using IPA platform for the comparison: GD patients vs. Controls.

ID	Molecules in Network	Top Diseases and Functions	IPA Score
1	ARHGAP21, B4GALT1, BST2, CHMP5, DUSP5, FOXC1, FOXF1, FOXF2, HERC5, **IFI6***, Ifn, IFN Beta, IRF, ISG15, LAP3, LGMN, LY96, NFkB (complex), Oas, OAS3, OASL, peptidase, Plk, PRDX3, PRSS3, RIPK4, SAA, SERPINB2, ST3GAL1, TFPI, Thioredoxin reductase, TNFAIP2, TRIM22, UBA6, UBE2L6	Infectious Diseases, Cell Signaling, Cell Morphology	48
2	AOX1, CAMK2N1, Cg, CLDN11, DNAJA3, DUSP6, ELOVL6, ERK, FJX1, FKBP1A, FSH,FSTL3, GK, GPC1, ID3, ITPR, KCNJ2, LAMTOR3, LATS2,Lh, MAP1LC3B, MAP2K1/2, Mek,N-cor, PFKFB3, PHLDA1, RASSF2, Ryr, SEMA3A, SLC20A1, Smad, SMAD9, SPRY2, TCF, **TMEM158**	Nervous System Development and Function, Developmental Disorder, Endocrine System Disorders	42
3	B2M, C1q, CLCF1, CRLF1, ERK1/2, hemoglobin, IFI44, IFIH1, IFIT2, IFIT3, IFN alpha/beta, IFN type 1, Ifnar, Ige, IgG, IgG1, Igg3, IgG2a, IgG2b, **IL13RA2,** Interferon-α Induced, IRF9, ISGF3, KLF13, LITAF, MHC Class I (complex), OAS1, OAS2, PARP9, RASGRP3, RND3, RSAD2, Sphk, WNK4, ZMPSTE24	Antimicrobial Response, Inflammatory Response, Neurological Disease	31
4	Akt, ANAPC13, calpain, CCNG1, CD24, CD47, COL4A5, collagen, Collagen Alpha1, Collagen type I, Collagen type IV, Collagen(s), CSRP2, Fgfr, Fibrin, Fibrinogen, HEY1, IDH1, IGFBP5, Integrin, Integrin alpha 4 beta 1, ITGA2, Laminin, LGALS3, PDHX, **PLAU**, PRKG2, Ptk, PTK7, SERPINH1, SIX5, Talin, THBS2, WLS, Wnt	Cardiovascular System Development and Function, Organismal Injury and Abnormalities, Cancer	31
5	Ap1, CAV1, CNN1, creatine kinase, CSRP1, CYTH2,e strogen receptor, Fcer1, GLUD1, Growth hormone, HMGA1, HPCAL1, **IFIT1**, IGF2BP3, KCNMA1, LDL, LY6E, MGST1, NADPH oxidase, NPLOC4, NPTX1, NT5E, p85 (pik3r), Pdgf (complex),P DGF BB, PI3K (complex), PI3K (family), Ras, SGCA, Shc, Sos, SPHK1, SSPN, STAT5a/b, trypsin	Skeletal and Muscular System Development and Function, Organismal Injury and Abnormalities, Respiratory Disease	29
6	ACAT1, ALDOC, Alp, CD3, CILP, Ck2, Creb, CTSL, DNAJB4, Gsk3, Hdac, HERC6, Histone h3, Histone h4, Hsp70, Hsp90, IFI16, IL1, Interferon alpha, KLF4, MT1G, MYLIP, Nfat (family), Nr1h, OSR1, PBX3, phosphatase, Pkc(s), PRNP, RNA polymerase II, SCO1, STAT1, TAP1, TBX2, UBLCP1	Antigen Presentation, Protein Synthesis, Endocrine System Disorders	29
7	20s proteasome, 26s Proteasome, AMPK, BCL2L1, BCR (complex), CASP1, caspase, Caspase 3/7, Cyclin A, Cyclin D, Cyclin E, cytochrome C, DLC1, DUB, E2f, HISTONE, IDH2, IFI44L, Ifn gamma, Jnk, LPIN1, MAP1LC3, PARP, PARP4, PARP12, PSME1, Rb, RGMB, TIPARP, TMEM126B, TXNIP, Ubiquitin, UCHL1, UCHL3, XAF1	Hereditary Disorder, Neurological Disease, Organismal Injury and Abnormalities	24

8	C1R, DMPK, DNER, ETNK2, FOS, GNG11, HNF4A, HSP90AA1, LZTS1, MOCOS, MRPL15, MRPL33, MRPL34, MRPL50, MRPL51, NAV3, NPTX2, NR3C1, OPTN, PARP4, PERP, POGK, RASD2, RPS6KC1, RTCA, SGK1, TCF19, TMEM109, TOR2A, TP73, TSHZ2, TTC4, TXNDC9, UBE2I, WNT10A	Cell Death and Survival, Respiratory Disease, Cancer	24
9	ARTN, C1QTNF5, CLDN8, CRIPAK, CRX, CSRP1, DUSP5, EED, EGF, EIF3A, ESR1, FAM98A, FKHR, GAREM1, GLIPR2, HCP5, **IFI6**, IFI44, Igf, IL20RA, IMP3, MAPK1, NT5DC2, PRSS12, RAB7, RAX2, RHBDF1, SAMD11, Serpina3g (includes others), STAT, TJP3, TRAF2, VPS29, VPS35, ZNF516	Cell-To-Cell Signaling and Interaction, Cell Cycle, Renal and Urological System Development and Function	24
10	ADO, AGFG2, ANKS6, APP, C6orf106, DENND1B, EIF5A, ELAVL1, FAM160B1, FYCO1, GLOD4, GLRX5, GMIP, GTPase, HERC6, IQSEC2, ITGB1BP1, LARP6, MEX3D, MSANTD4, NOL7, PRL, PROSER2, SAMD9, SAMD9L, SLC35F5, SNX21, SPIN1, SPIN4, THOC6, TMA7, TRUB1, XPO1, YWHAE, ZFYVE1	Molecular Transport, RNA Trafficking, Embryonic Development	20
11	7S NGF, ALDH9A1, Alpha catenin, CCDC85B, CNPY3, CTSF, DDIT4L, DNAJC13, ERVW-1, FBXO42, GOLGA8A/ GOLGA8B, GPD1L, HERC5, HSPA5, HSPB1, IARS2, IER5L, IL6, **ISLR**, LILRA3, LPIN2, LRRC40, miR-19b-3p (and other miRNAs w/seed GUGCAAA), MXRA8, NARS, POU5F1, PPP1CA, PTPRU, SERPINC1, SLC39A14, SLC7A14, THBS2, TP53, TWIST1, UBL5	Cellular Compromise, Cell Cycle, Cellular Development	20
12	ABLIM3, ADGRG2, ADGRL4, ADRA2C, ADRB2, AHR, **ATOH8**, Calmodulin, CYSLTR2, DLG4, Focal adhesion kinase, Gpcr, GPR35, GPR55, GPR146, GPR160, GPR171, GPR176, GPR180, HTR2B, HTR5A, ITM2C, Mitochondrial complex 1, Mmp,MT-ND4L, NDUFAB1, NDUFAF2, NNMT, Pka, PLC, RGS17, S1PR3, TGFB1, TMX1, ZSWIM4	Psychological Disorders, Cardiac Thrombosis, Cardiovascular Disease	13
13	Actin, ADRB, ARHGEF17, Cofilin, **CRISPLD2**, DNER, ERCC5, ETV5, HIGD1A, Iga, Igm, IKK (complex), IL12 (complex), IL12 (family), Immunoglobulin, Insulin, MAP3K8, Mapk, MTORC1, Notch, P38 MAPK, PP2A, Pro-inflammatory Cytokine, Proinsulin, Rac, Ras homolog, Secretase gamma, SRC (family), TCR, Tgf beta, Tlr, Tnf (family), TXNDC17, Vegf, YPEL3	Cellular Movement, Cellular Development, Cellular Growth and Proliferation	11
14	ADRA1B, ANP32B, APC (complex), AZGP1, BAG1, C1QBP, CCND1, CDH1, CPNE3, DGCR8, DNAJB4, EDN3, EPCAM, ETV5, Fgf, GBAS, GPI, histone deacetylase, KLF8, KLK3, LYPLAL1, MRPS10, MST1R, PTPRF, PTPRU, PTTG1, RAP1GAP, RND3, SFRP4, SS18L2, ST6GALNAC1, TPBG, WNT4, ZNF503, ZNF510	Cellular Movement, Cancer, Organismal Injury and Abnormalities	10
15	KLHL40, LMOD3	Developmental Disorder, Hereditary Disorder, Organismal Injury and Abnormalities	2

*genes coding for molecules which are shaded and underlined were analyzed by microarray and qRT-PCR studies

Supplementary Table S7. Networks of molecules in interactions for the differentially expressed genes by using IPA platform for the comparison: GD patients vs. NPC patients.

ID	Molecules in Network	Top Diseases and Functions	IPA Score
1	Alpha catenin, ANGPTL2, CHN1, COL4A5, collagen,Collagen type I, Collagen type IV, Collagen(s), DLC1,EEF1E1, EIF2B2, EPRS, estrogen receptor, FAM21A/FAM21C, FBLN2,FOXQ1, Growth hormone, GTPase, IARS, IGF2BP3, Laminin, LARS, LDL-cholesterol, LY6E, MAFB, MARS, MMP2, MTHFD2, PI3K (complex), **PLAU***, PLOD2, RAB3IL1, RGS2, TFAP2C, VPS29	Connective Tissue Disorders, Skeletal and Muscular Disorders, Cancer	39
2	AKR1B1, Alp, APC/APC2, BMP, BZW2, Cbp/p300, Collagen Alpha1, CXXC5, ERK1/2, FOXF1, FOXF2, HEY1, histone deacetylase, ID1, ID3, Importin beta, KLF13, LITAF, LMO4, **MN1**, PFKFB4, PROS1, SH2B3, SLC7A8, Smad, SMAD6, SMAD7, SMAD9, Smad1/5/8, TCF21, Tgf beta, TGFBR, THOC6, UBA6, WNT5A	Gene Expression, Cardiovascular System Development and Function, Embryonic Development	37
3	BST2, ERK, GBP1, GK, IFI44L, **IFIT1**, IFIT2, IFIT3, Ifn, IFN alpha/beta, IFN Beta, Ifn gamma, IFN type 1, Ifnar, Immunoproteasome Pa28/20s, Interferon-α Induced, ISG15, ISGF3, LAP3, **MX1**, Oas, OAS1, OAS2, OAS3, OASL, PSMA, PSMA6, PSMC2, PSMD14, PSME2, PSMG1, RSAD2, STAT1, WARS, ZC3H15	Antimicrobial Response, Inflammatory Response, Cell Signaling	37
4	ADAM19, Akt, AMPK, CCDC50, CLDN1, Cyclin D, cytochrome C, ETV5, FAM129B, Fgfr, GFPT1, glutathione peroxidase, KLF6, LDH (family), LDHA, LDHB, MTORC2, MXRA8, N-Cadherin, OLFML3, OXSR1, PARP, PARP12, PMAIP1, PRKAA, PRRX2, PTPase, Sod, STK39, THBS2, TWIST1, Ubiquitin, UCHL1, UCHL3, XAF1	Cardiovascular System Development and Function, Embryonic Development, Organ Development	33
5	Calcineurin A, CAV2, CDCP1, Cg, Cofilin, DUSP5, DUSP6, DUSP10, EPS8, F Actin, Hsp27, Jnk, KLHL21, KPNB1, LDL, Mek, MGST1, Nos, OLR1, Pdgf (complex), PDGF BB, PFN2, phosphatase, PPP4R1, PTS, RHOB, RND3, Rock, SACM1L, SEMA3A, Sos, SRXN1, **TMEM158,** VEGFC, ZZZ3	Cell Morphology, Cellular Assembly and Organization, Cell Death and Survival	33
6	ABCE1, AHCY, AK2, ATP synthase, ATP5C1, ATPase, BTG2, CTPS1, CTSA, E2f, Fgf, FOS, GSPT1, Hdac, Histone h4, HLA-C, MAPRE1, MHC Class I (complex), N-cor, NAV3, NCKAP1, ODC1, P4HA1, PRMT3, Rb, RNA polymerase I, Rxr, SLC20A1, SPRY2, STRAP, Tap, TAP1, TCF, thymidine kinase, USO1	Post-Translational Modification, Molecular Transport, Protein Trafficking	33
7	Ck2, CRIP1, DACT3, DDX10, EIF5A, Focal adhesion kinase, FSH, GTF2H2C, HISTONE, HPRT1, IFI16, IGDCC3, KPNA4, Lh, MTORC1, MYC, MYPN, NARS, NOC3L, NXN, PAICS, PCDHAC2, PELO, PERP, PLC, POLR2H, PRDX3, Rac, Ras homolog, RNA polymerase II, RPF2, SCYL2, TPI1, XPO1, ZNF83	Cardiovascular Disease, Hereditary Disorder, Organismal Injury and Abnormalities	33

8	20s proteasome, B4GALT1, BCR (complex), CITED2, DOCK10, Fcer1, GLRX, HINT1, Iga, Ige, Ikb, Ikk (family), MAP3K8, ME1, MHC CLASS I (family), Nfat (family), NFKB1, NFkB (complex), peptidase, PI3K (family), Plk, PSMA4, PTGIS, RAP1GDS1, RASD2, RBCK1, RIPK4, SERCA, SLC16A3, SLC2A1, SPATS2L, SYK/ZAP, T3-TR-RXR, TFRC, TNFAIP2	Lipid Metabolism, Molecular Transport, Small Molecule Biochemistry	28
9	Actin, ACTR3, Alpha tubulin, Ap1, Beta Tubulin, BRIX1, CCT2, CCT8, CD3, CKAP4, DNER, EBNA1BP2, HIGD1A, Histone h3, IL1, Insulin, Mapk, MT1G, NME1, Notch, p85 (pik3r), PGAM1, POLE3, PPA1, PRDX6, PRPF4, SCO1, Secretase gamma, Shc, SRC (family), STAT5a/b, TCR, TXNDC9, VBP1, YARS	Cellular Assembly and Organization, Cell-To-Cell Signaling and Interaction, Reproductive System Development and Function	28
10	ADH6, APP, BCL2, CCDC80, CCND1, CINP, CPNE3, CRACR2B, CTNNAL1, EOGT, FAM212B, GHITM, GLRX5, GPT2, HERC6, IARS2, IFI6, KRAS, LAP3, miR-451a (and other miRNAs w/seed AACCGUU), MTHFR, NDUFB1, NOL7, OAS2, PGM1, PRL, RDH14, SAMD9, SAMD9L, SEC11C, SLC35B1, SP2, TMEM171, UQCRHL, ZHX2	Cellular Development, Cellular Growth and Proliferation, Endocrine System Development and Function	26
11	26s Proteasome, APPL2, ASS1, C1QBP, C1QTNF5, Calcineurin protein(s), calpain, caspase, CDR2, Cyclin A, Cyclin E, DDX47, DNAJA3, DNAJB4, Fibrinogen, GOT, HDL, HSP, Hsp70, Hsp90, HSP90B1, HSPA9, HSPB7, IL12 (complex), Integrin, MAP2K1/2, PDHX, Pkc(s), PP2A, PRNP, Pro-inflammatory Cytokine, SGCA, STK4, Tlr, TSH	Embryonic Development, Organismal Development, Tissue Morphology	20
12	A4GALT, Adaptor protein 2, ADRB, BCL2L13, BNIP3, CaMKII, CHMP5, Creb, CRLF1, ETFA, Gsk3, GTF3A, IgG, IgG1, Igm, IKK (complex), IL12 (family), Immunoglobulin, Interferon alpha, Mitochondrial complex 1, Mmp, NDUFA8, NDUFAB1, NDUFAF2, P38 MAPK, Pka, PRSS3, RAB32, Ras, trypsin, Vegf, WLS, Wnt, YPEL3, ZMPSTE24	Connective Tissue Disorders, Dermatological Diseases and Conditions, Developmental Disorder	20
13	BLZF1, CAB39L, CCDC74A, CDC37L1, EED, FBXO45, FKBP5, GARS, GSK3B, HERC4, HERC6, HGH1, HS1BP3, HSP90AA1, IMP3, KDM1A, LRRC40, Macf1, MORF4L2, OSTC, PRKAG2, PRKG2, SIX5, STRADB, TARS, TIMM10, TMED3, TMOD4, TP73, TSHZ2, TTC4, UAP1L1, UNC45B, VDAC1, ZCCHC14	Metabolic Disease, Neurological Disease, Psychological Disorders	20
14	ANAPC13, ARHGAP22, CDH1, Cg, CYP17A1, DCBLD1, ENPP3, ESM1, FJX1, GBP6, LRIG1, MAN1A2, mir-210, MRPL14, MRPL15, MRPL17, MRPL21, MRPL32, MRPL34, MRPL36, MRPL50, MRPS6, MTERF3, NADH dehydrogenase, NGRN, RPAIN, SCARA3, SLC12A6, SLC39A6, TBC1D8, TNF, TPBG, TST, ZC3H3, ZNF503	Cellular Development, Hematopoiesis, Cellular Assembly and Organization	17
15	ACAN, ADAMTS6, ADAMTS7, **ATOH8**, ATP13A3, B4GALNT1, C9orf3, CCDC85B, COL16A1, COMP, DCUN1D5, FETUB, GBP1, HDDC2, IL4,IL26, KIRREL3, LILRA4, MAPK14, Metalloprotease, miR-146a-5p (and other miRNAs w/seed GAGAACU), MLLT11, MT1A, OLFML2B, OSM, RNF152, SLC15A1, SPON2, STAT, TGFB1, TNF, TRIM21, UBE2E1, VWCE, ZNF330	Cellular Movement, Hematological System Development and Function, Immune Cell Trafficking	13

16	ADO, ANXA3, BMP7, CASP8AP2, DNER, ESR1, FAM167A, GLIPR1, GPSM2, HCP5, HRAS, ICA1, IFITM2, INHBC, INHBE, **ISLR,** JUNB, KNDC1, LRMP, MSANTD4, Ncoa6, NR3C1, PCYOX1, PLEKHF1, Prl2c2 (includes others), Proinsulin, PTP4A2, SAMD11, SERP1, SLC25A20, SYTL2, TCF7L2, TMEM2, TMF1, YWHAE	Cell Cycle, Connective Tissue Development and Function, Cancer	13
17	EIF1B, GAN, **MXRA5**	Hereditary Disorder, Neurological Disease, Organismal Injury and Abnormalities	2

* genes coding for molecules which are shaded and underlined were analyzed by microarray and qRT-PCR studies

Supplementary Figure S8. Networks of the differentially expressed genes by using IPA platform for the comparison: GD patients vs. NPC patients.

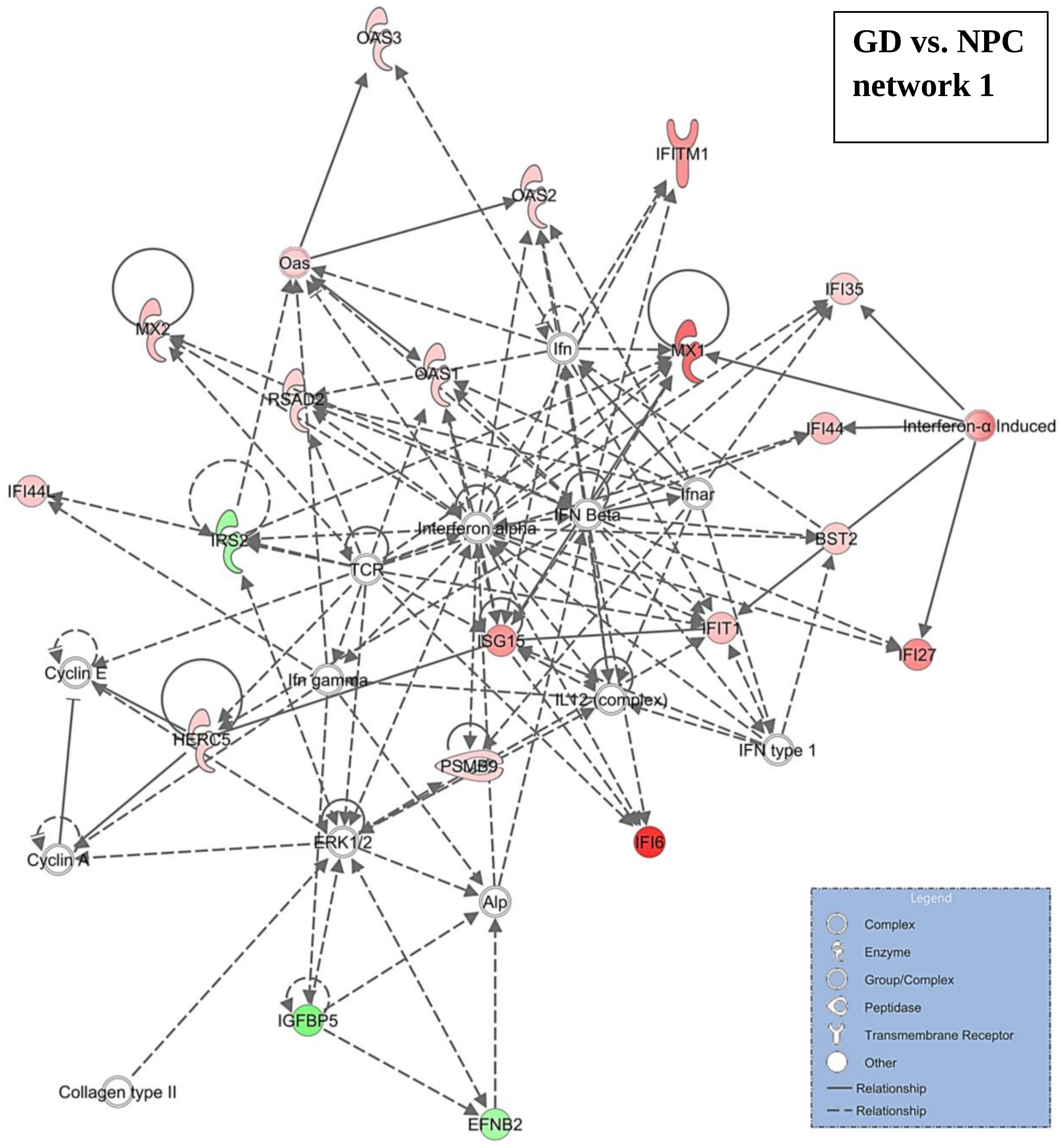

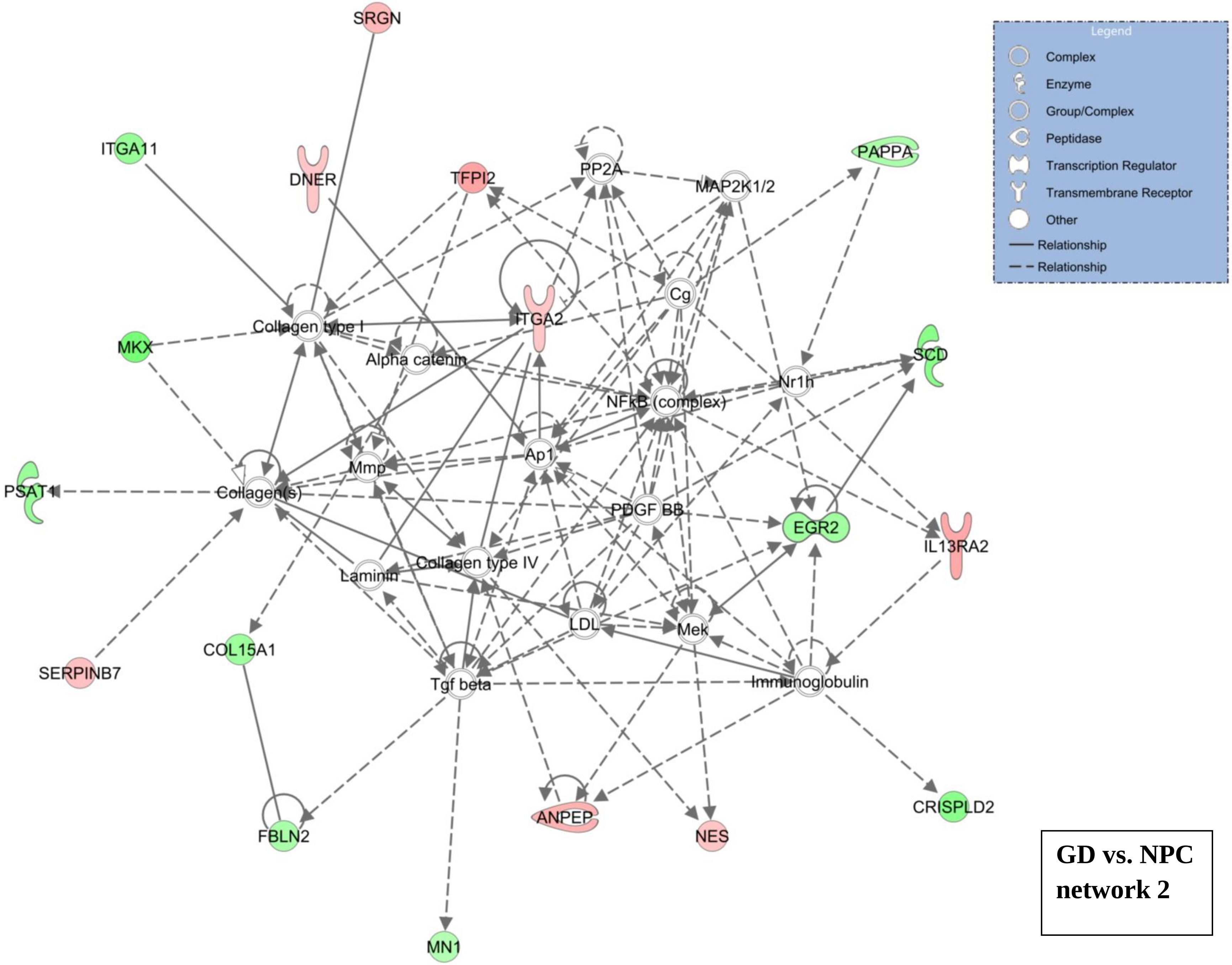

GD vs. NPC network 2

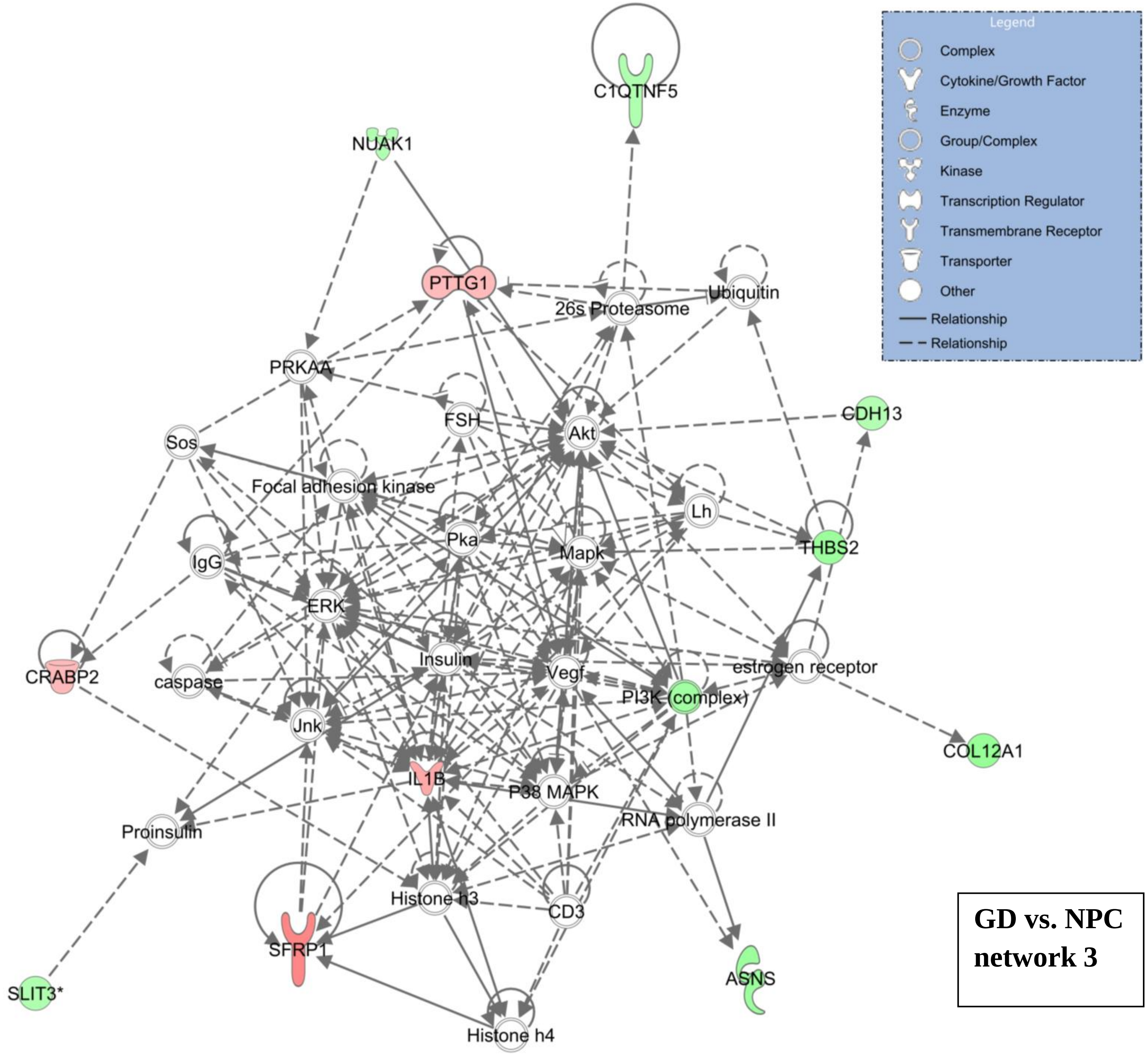

GD vs. NPC network 3

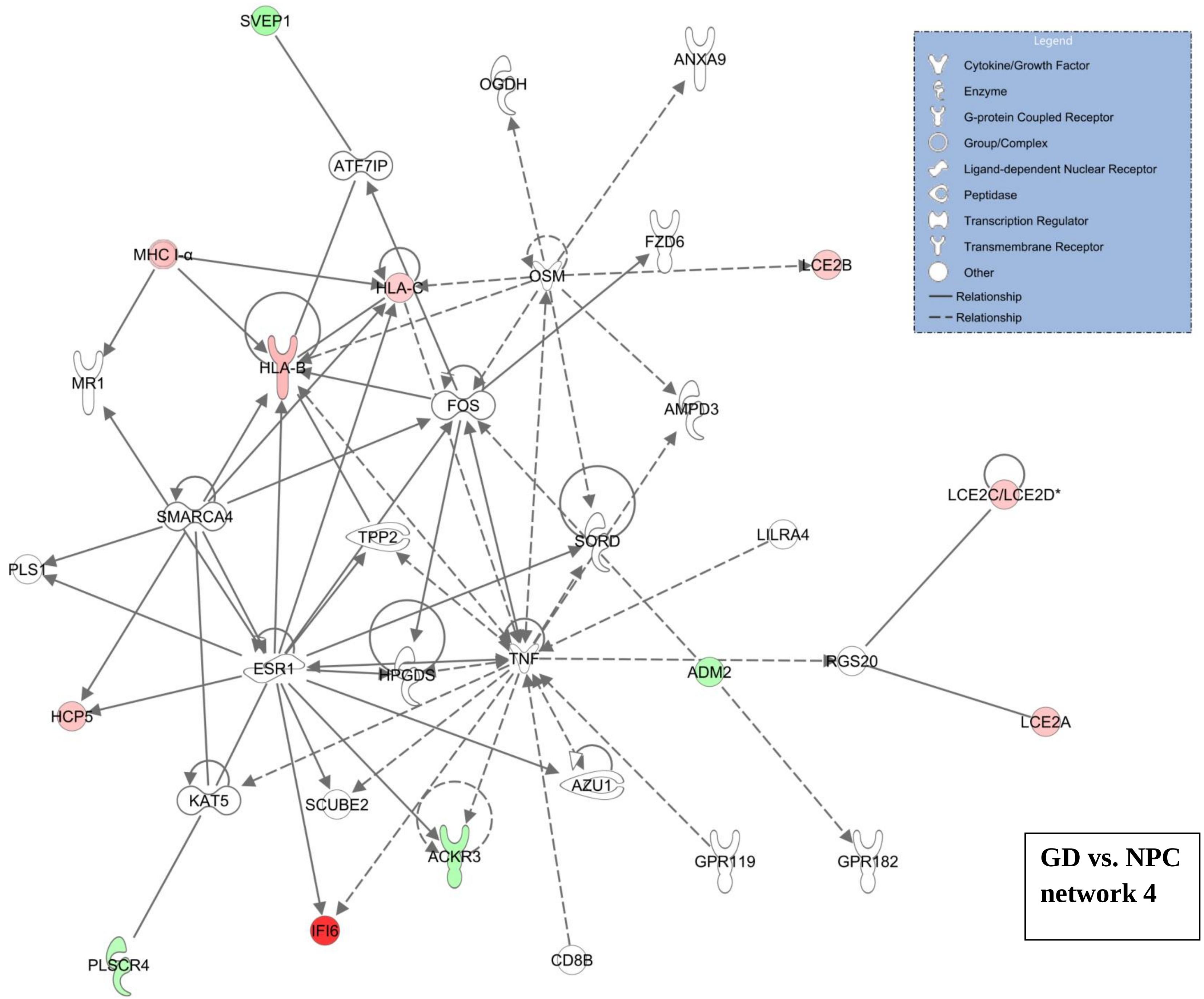

GD vs. NPC network 4

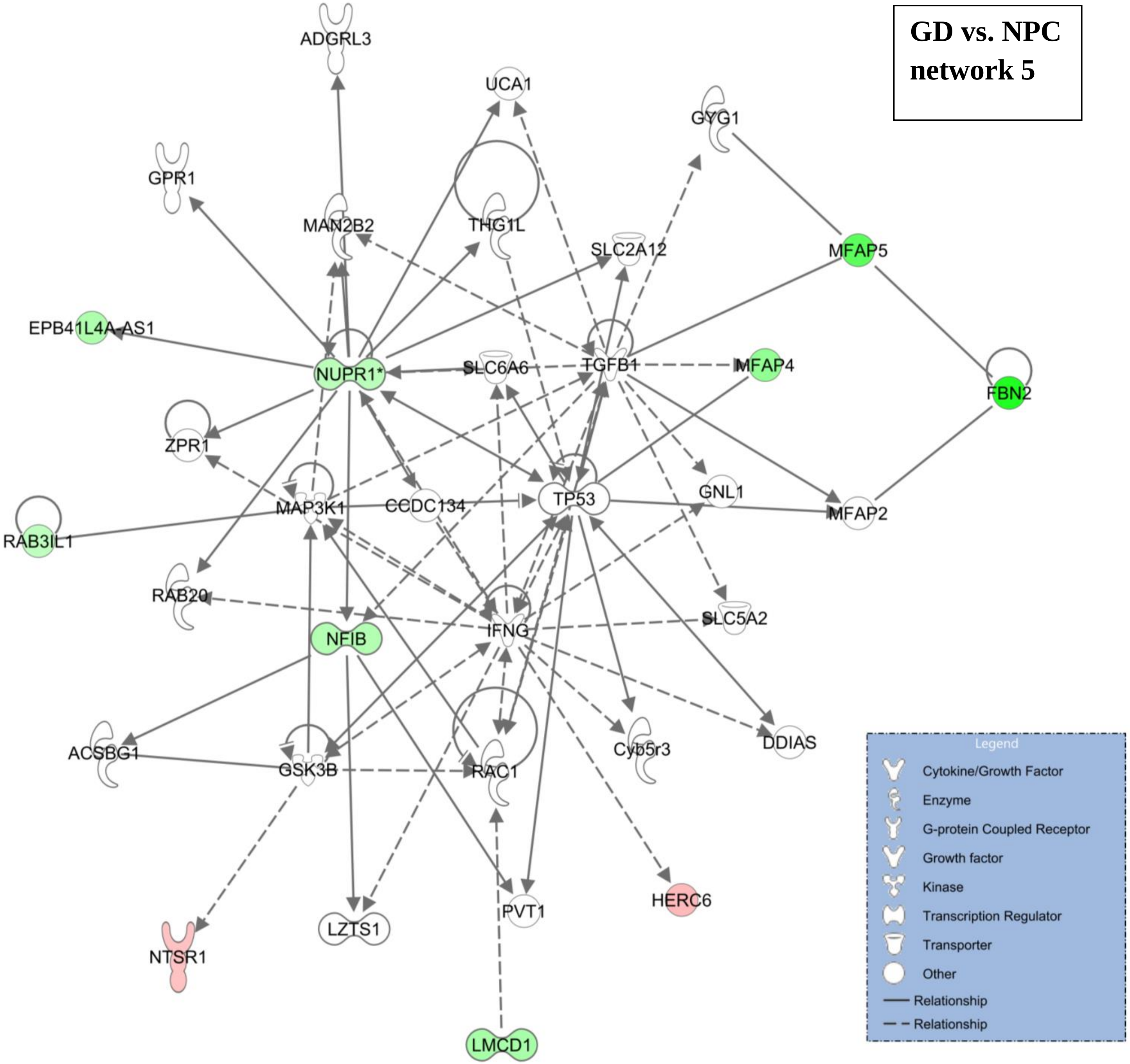
GD vs. NPC
network 5
ADGRL3
UCA1
GYG1
GPR1
MAN2B2
THG1L
SLC2A12
MFAP5
EPB41L4A-AS1
NUPR1*
SLC6A6
TGFB1
MFAP4
FBN2
ZPR1
GNL1
MAP3K1
CCDC134
TP53
MFAP2
RAB3IL1
RAB20
SLC5A2
NFIB
IFNG
ACSBG1
GSK3B
RAC1
Cyb5r3
DDIAS
HERC6
NTSR1
LZTS1
PVT1
LMCD1
Legend
Cytokine/Growth Factor
Enzyme
G-protein Coupled Receptor
Growth factor
Kinase
Transcription Regulator
Transporter
Other
Relationship
Relationship

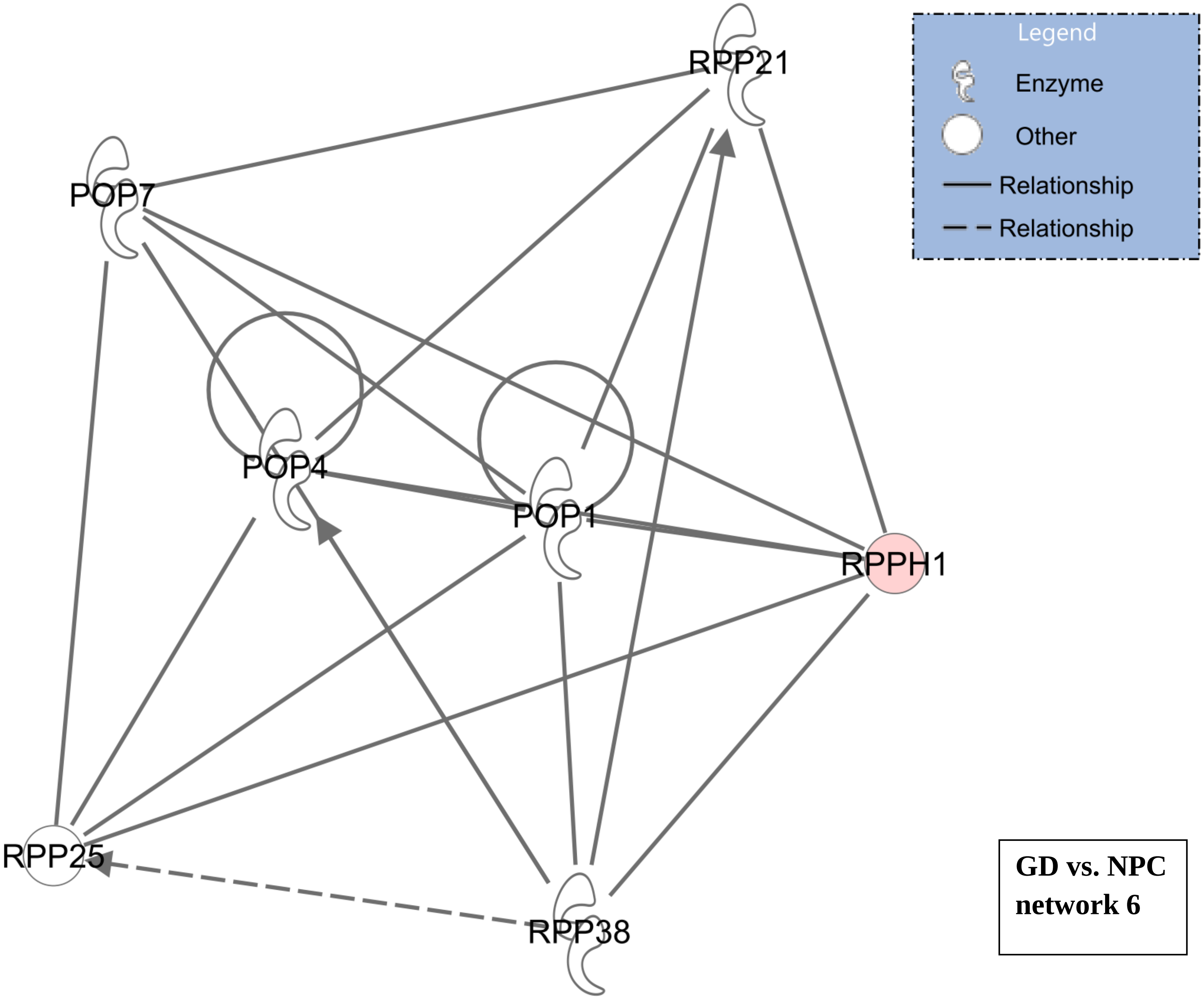

GD vs. NPC network 6

Supplementary Table S9. Genes studied and probes used in qRT-PCR analyses.

Gene	Life Technologies Probes
**Analysis 'Gaucher vs. Control'**	
SERPINB2	Hs01010736_m1
IL13RA2	Hs00152924_m1
PLAU	Hs01547054_m1
IFI6	Hs00242571_m1
TXNIP	Hs01006900_g1
IGFBP5	Hs00181213_m1
CRISPLD2	Hs00230322_m1
THBS2	Hs01568063_m1
ATOH8	Hs01031629_m1
NNMT	Hs00196287_m1
**Analaysis 'Gaucher vs. NPC'**	
MX1	Hs00895608_m1
TMEM158	Hs00374916_s1
UCHL1	Hs00985157_m1
ISG15	Hs01921425_s1
PLAU	Hs01547054_m1
PTGIS	Hs00919949_m1
ATOH8	Hs01031629_m1
MXRA5	Hs01019147_m1
MN1	Hs00159202_m1
FOXQ1	Hs00536425_s1
**Reference genes**	
PUM1	Hs00472881
SDHA	Hs00417200

Supplementary Table S10. Statistically significant differences in the ddCt values - analysis of results obtained in GD patients and Controls.

	ddCt	ddCt		
Gene	GD patients	Controls	P* Mann-Whitney test	P t-Student test**
	n=5	n=5		
SERPINB2	1.7955 [0.8378-3.4472]	0.6045 [0.1857-0.9903]	0.151	0.077
IL13RA2	0.9655 [0.4475-1.0890]	0.0597 [0.0110-0.1594]	0.008	0.007
PLAU	1.7297 [1.1790-1.9834]	1.2501 [0.3067-5.4708]	1.000	0.791
IFI6	1.3648 [0.0655-2.2643]	0.1173 [0.0502-0.1436]	0.222	0.168
TXNIP	0.6617 [0.3090-0.8480]	0.5780 [0.3216-1,1399]	1.000	0.775
IGFBP5	0.0686 [0.0279-0.0805]	0.1814 [0.1585-0.1931]	0.421	0.532
CRISPLD2	0.0562 [0.0319-0.1478]	1.7016 [0.1076-1.7016]	0.095	0.057
THBS2	0.2370 [0.0618-0.3675]	2.9173 [1.0189-2.9173]	0.008	0.002
ATOH8	0.0025 [0.0016-0.0487]	0.1098 [0.0393-0.2956]	0.063	0.091
NNMT	0.0536 [0.0290-0.0824]	0.7258 [0.5411-0.7258]	0.016	0.002

Data are presented as medians and interquartile ranges from first to third quartile.
*A result is statistically significant when P < 0.05
** Variables were logarithmically transformed in statistical analysis.

Supplementary Table S11. Statistically significant differences in the ddCt values - analysis of results obtained in GD patients and NPC patients.

Gene	ddCt GD patients n=5	ddCt NPC patients n=5	P* Mann-Whitney test	P t-Student test**
MX1	0.1530 [0.0067-0.2171]	0.0229 [0.0213-0.0333]	0.421	0.345
TMEM158	0.7064 [0.4861-2.0786]	0.5789 [0.3727-1.3480]	1.000	0.988
UCHL1	3.7895 [1.4849-4.6394]	10.0139 [9.1342-10.4285]	0.008	0.009
ISG15	0.5225 [0.3093-0.5590	0.6090 [0.4881-0.6703]	0.548	0.699
PLAU	1.7297 [1.1790-1.9834]	0.6592 [0.5987-0.9978]	0.222	0.143
PTGIS	0.4643 [0.0166-0.9778]	1.5476 [1.0997-1.6428]	0.310	0.250
ATOH8	0.0025 [0.0016-0.0487]	1.2724 [0.8737-1.3571]	0.016	0.011
MXRA5	0.3414 [0.3003-0.4857]	2.4863 [1.5311-3.2034]	0.095	0.037
MN1	0.2538 [0.1325-0.2612]	1.9104 [1.3138-1.9294]	0.008	0.023
FOXQ1	0.0349 [0.0187-0.0578]	0.1565 [0.0772-0.1897]	0.032	0.020

Data are presented as medians and interquartile ranges from first to third quartile.
*A result is statistically significant when $P < 0.05$
** Variables were logarithmically transformed in statistical analysis.
